# The role of emerging elites in the formation and development of communities after the fall of the Roman Empire

**DOI:** 10.1073/pnas.2317868121

**Published:** 2024-08-19

**Authors:** Yijie Tian, István Koncz, Sarah Defant, Caterina Giostra, Deven N. Vyas, Arkadiusz Sołtysiak, Luisella Pejrani Baricco, Rafał Fetner, Cosimo Posth, Guido Brandt, Elena Bedini, Alessandra Modi, Martina Lari, Stefania Vai, Paolo Francalacci, Ricardo Fernandes, Axel Steinhof, Walter Pohl, David Caramelli, Johannes Krause, Adam Izdebski, Patrick J. Geary, Krishna R. Veeramah

**Affiliations:** ^a^Department of Ecology and Evolution, Stony Brook University, Stony Brook, NY 11794; ^b^Department of Humanities, Institute of Archaeological Sciences, Eötvös Loránd University, Budapest 1088, Hungary; ^c^Department of History and Cultural Studies, Institute of Prehistoric Archaeology, Freie Universität Berlin, Berlin 14195, Germany; ^d^Max Planck Institute of Geoanthropology, Jena 07743, Germany; ^e^Department of Philosophy and Humanities, Institute of Greek and Latin Languages and Literatures, Freie Universität Berlin, Berlin 14195, Germany; ^f^Department of History, Archaeology and Art History, Catholic University Milan, Milan 20103, Italy; ^g^Department of Bioarchaeology, Faculty of Archaeology, University of Warsaw, Warszawa 00-927, Poland; ^h^Soprintendenza Archeologia, Belle Arti e Paesaggio per la città metropolitana di Torino, Torino TO 10122, Italy; ^i^Archaeo- and Palaeogenetics, Institute for Archaeological Sciences, Department of Geosciences, University of Tübingen, Tübingen 72074, Germany; ^j^Department of Archaeogenetics, Max Planck Institute for Evolutionary Anthropology, Leipzig 04103, Germany; ^k^Department of Biology, University of Florence, Firenze 12-50122, Italy; ^l^Dipartimento di Scienze della Vita e dell'Ambiente, Università di Cagliari, Cagliari 09126, Italy; ^m^Arne Faculty of Arts, Masaryk University, Brno-střed 602 00, Czech Republic; ^n^Climate Change and History Research Initiative, Princeton University, Princeton, NJ 08542; ^o^Max Planck Institute for Biogeochemistry, Jena 07745, Germany; ^p^Institute for Medieval Research, Austrian Academy of Sciences; Institute for Austrian Historical Research, University of Vienna, Vienna 1020, Austria; ^q^Institute of History, Jagiellonian University in Krakow, Kraków 31-007, Poland; ^r^School of Historical Studies, Institute for Advanced Study, Princeton, NJ 08540

**Keywords:** paleogenomics, burial archaeology, isotope, mobility, late antiquity

## Abstract

Elites played a pivotal role in the formation of post-Roman Europe on both macro- and microlevels during the Early Medieval period. History and archaeology have long focused on their description and identification based on written sources or through their archaeological record. We provide a different perspective on this topic by integrating paleogenomic, archaeological, and isotopic data to gain insights into the role of one such elite group in a Langobard period community near Collegno, Italy dated to the 6-8th centuries CE. Our analysis of 28 newly sequenced genomes together with 24 previously published ones combined with isotope (Sr, C, N) measurements revealed that this community was established by and organized around a network of biologically and socially related individuals likely composed of multiple elite families that over time developed into a single extended pedigree. The community also included individuals with diverse genetic ancestries, maintaining its diversity by integrating newcomers and groups in later stages of its existence. This study highlights how shifts in political power and migration impacted the formation and development of a small rural community within a key region of the former Western Roman Empire after its dissolution and the emergence of a new kingdom. Furthermore, it suggests that Early Medieval elites had the capacity to incorporate individuals from varied backgrounds and that these elites were the result of (political) agency rather than belonging to biologically homogeneous groups.

## Significance

Elites played a pivotal role in the formation of post-Roman Europe on both macro- and microlevels during the Early Medieval period. Our approach combines history and archaeology with paleogenomic and isotopic data to explore the role of elite groups in the development of a 6 to 8th-century community at Collegno, Italy. Analyzing 28 new genomes with 24 previous ones revealed that the site was formed around biologically and socially connected high-ranking groups. The community also integrated newcomers and embraced individuals with diverse genetic ancestries. This study highlights how power shifts and migration after the fall of the Roman Empire influenced community formation in the rural areas in one of the core territories of the former Western Roman Empire.

The dissolution of existing communities and the establishment of new ones are frequent consequences of political shifts or social changes, especially if these are also connected to the relocation of certain groups and migration of people. It has been theorized that in situations like these the importance of various elites—whether intellectual, religious, military, etc.,—are likely to increase (1). To date, most research on elites present during historical time periods has relied on textual and mortuary data to identify and describe them (2–4). However, combining these well-explored approaches with new paleogenomic and isotopic data has the potential to provide a more comprehensive description of how these elite groups were formed and organized, how status was achieved or inherited, and what their relationship was with the rest of the population. Such information is vital for understanding what role elites play in maintaining or recreating the cohesion of groups and whether or not they could be understood as the driving force behind the formation of new communities.

Across Western and Central Europe during the so-called Migration Period (ca. 4th to 9th century CE) new political systems, *regna*, or kingdoms, created by newly formed (warrior) elites, came to dominate the political and social hierarchies on the territory of the former Western Roman Empire (5, 6). One well-known example is when the Langobard King Alboin migrated with his polyethnic followers from Pannonia in 568 and founded his kingdom in Italy. By then, the peninsula had experienced decades of brutal war which had caused massive social, economic, and institutional destruction due to the decades-long attempt by Roman (Byzantine) armies to reconquer Italy. As such, the emergence of these “barbarian” kingdoms meant more than just political change, it also marked social and cultural transition (7).

**Fig. 1.**
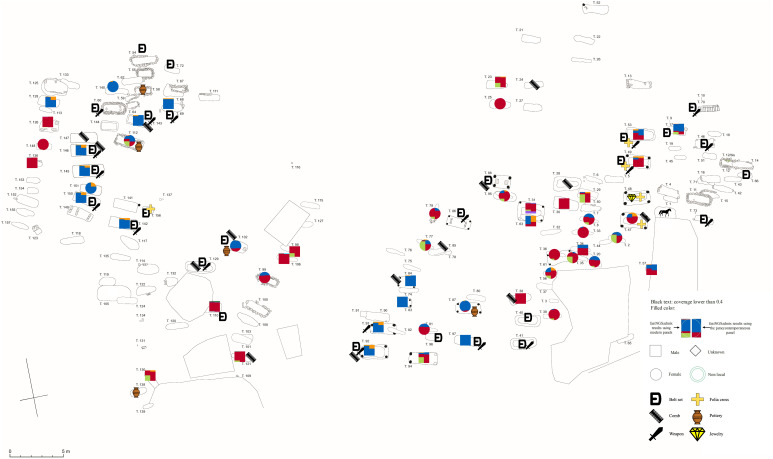
The Langobard-period cemetery of Collegno. Individuals with available genetic data were marked with either a circle (female) or a square (male), representing their estimated genetic ancestry of two model-based clustering analyses as described below. Additionally, artifacts such as weapons, belt sets, and gold folia crosses were also marked on the map.

The new arrivals formed a minority compared to the local population of Italy. The highest elites, kings, dukes, and other officers occupied the important urban centers, such as Verona, Milan, Pavia, and Turin, to maintain preexisting administrative focuses as a town-based regnum (8, 9). They exercised their power in the rural areas through the occupation of strategically important locations by newly emerging warrior elites that replaced or assimilated local landowners and founded new communities (10). The cemetery at Collegno, located near Turin, Northwest Italy (Fig. 1 and *SI Appendix*, Fig. S1) belonged to such a community, established during the early phase of the Langobard occupation, at the end of the 6th century and remained in use for at least a century (11, 12).

A previous study of Collegno analyzed 24 individuals and revealed large patrilineal biological kindreds in the earliest phase, with varying degrees of Central and Northern European genetic ancestry and nonlocal provenance (13). In this study, we complemented the earlier sampling with an additional 28 individuals that represent the cemetery’s whole span of existence, as well as additional Sr, C, and N isotopic analyses. We first used these data in an integrative framework in order to describe the formation and development of the community buried at Collegno in a greater detail than was previously possible. Then, building upon this internal chronological framework, we investigated the role biological relatedness played at the site and explored the relationship between an extensive, multigenerational pedigree and the rest of the community.

## Results

### Collegno—Description of the Site and Sampling.

Collegno is located 7 km west of Turin, Northwest Italy, in Piedmont, near a crossing of the river Dora. Between 2002 and 2006 a complex archaeological site was excavated along the important road leading to Val di Susa and the Alpine passes that connected Italy and Gaul. Settlement remains and burials forming two chronologically different cemeteries came to light: a smaller cemetery, consisting of 8 burials from the Gothic period (early/mid-6th century) and a large cemetery dated to the Langobard period. Archaeological evidence suggests that the settlement area was used by communities throughout the two periods, but there was no continuity between them (11, 12).

Altogether, 157 graves can be dated to the Langobard period (Fig. 1) that contained 149 human individuals (with a higher ratio of males among adults: 65 males compared to 40 females, but with similar age-at-death distribution) and a horse, while 7 graves were without any observable osteological remains (11, 14) (Supplementary section S1). The graves are all west-east oriented and form more or less regular north–south rows. Graves are more densely organized in the north-western and eastern parts of the cemetery, forming two distinguishable but not clearly separable burial groups. Archaeological dating based on artifact types, changes in burial customs, and numerous superpositions between graves suggest that use of the site spanned at least a century divided into 3 phases (11, 13, 14). The site was founded in the late 6th century, during the early phase of the Langobard occupation in Italy. The early burials have distinctive constructions in the form of posthole structures and contain a relatively large number of artifacts, such as jewelry, weapons, elaborate belt sets, and tools, which show strong cultural connections to Merovingian Western Europe, but also to 6th-century Middle Danube region (11, 12). Due to the general disappearance of grave goods during the 7th century (15), the abandonment of the site is much harder to date archaeologically, but the high number of simple pit graves without any artifacts, typical for later seventh and eighth-century burials, suggests that the site remained in use throughout the 7th and at least the beginning of the 8th century (11, 13).

We generated genomic data from 28 new samples (Dataset S1, Supplementary section S2) from Collegno. Illumina sequencing of DNA extracts from petrous bone and auditory ossicles was conducted, for which there was high endogenous content, high library complexity, and patterns of postmortem damage (PMD) characteristic of ancient DNA. 13 samples belonged to males and 15 to females. Analysis of X chromosome–mapped reads in males and mtDNA in both sexes revealed low levels of estimated contamination in all individuals (mean ~1%). Genomic libraries for the 28 individuals underwent partial UDG treatment and then underwent an in-solution capture targeting ~1.2 M single nucleotide polymorphisms (SNPs) (henceforth 1,240 K capture). The average coverage at these SNPs was ~1.39×. In our downstream analysis, we used all 28 individuals and combined them with 24 previously published individuals, resulting in a total of 52 individuals equally representing all burial groups and phases of the site and restricted only by the availability and preservation of the bone material (14).

### Genetic Diversity and Pedigrees.

We investigated the genetic diversity of all 52 individuals using principal component analysis (PCA), which was based on modern Eurasian populations from Affymetrix Human Origins array data found in the Allen Ancient DNA Resource v50.0 (16–22) (Dataset S2), as well as a dataset obtained from the European POPRES database (23) (Supplementary Section S3). We found that our ancient individuals had genetic ancestry that overlapped substantially with modern Europeans (but no other continental-scale non-European regions) and demonstrated a diverse distribution, widely spreading along a Northern to Southern European axis (*SI Appendix*, Fig. S4 and S5).

**Fig. 2.**
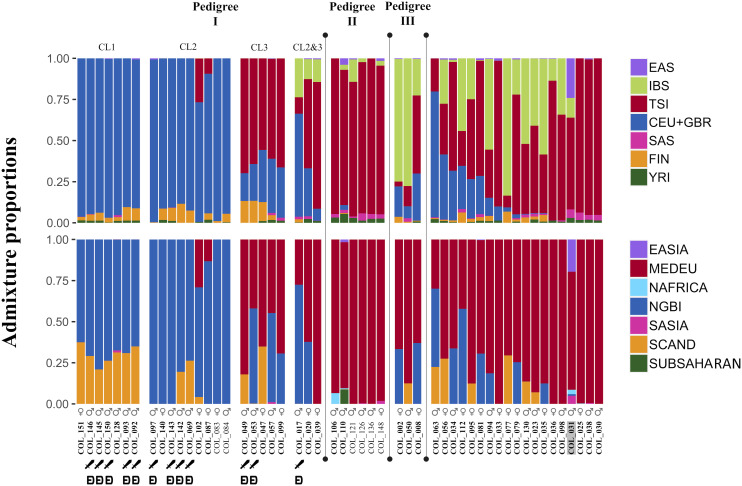
Supervised ancestry proportions from individuals from Collegno.The ♂ and ♀ symbols identify genetic males and genetic females, respectively, while the unbolded texts identify nonadults. The presence of weapons and/or belt sets is also marked. In the upper figure proportions were estimated using 1000 Genomes populations as references (CEU+GBR: Northern Europeans from Utah and British in England and Scotland; FIN: Finnish in Finland; IBS: Iberian populations in Spain; TSI: Tuscans from Italy; EAS: the East Asian superpopulation; SAS: the South Asian superpopulation; and YRI: Yoruba in Ibadan, Nigeria). In the bottom figure, proportions were estimated using penecontemporaneous populations. (MEDEU: Italy and Iberia (Mediterranean Europe); NGBI: what are now northern Germany and Britain; SCAND: what are now Scandinavia/Estonia; EASIA: what is now Hanben, Taiwan; NAFRICA: what is now Sudan;SASIA: Roopkund Lake in what is now India; SUBSAHARAN: multiple sites from sub-Saharan Africa). Individuals are sorted based on biological relatedness and by decreasing NGBI for unrelated individuals.

To further explore the genetic structure of the Collegno community, we used a model-based clustering method (fastNGSadmix) (24) to characterize the genomic ancestry of each Collegno individual using seven 1000 Genomes Project (1000G) (25) populations as references (13). In addition, we also used a penecontemporaneous reference panel (spanning from the 4 to 8th centuries) previously described in Vyas and Koncz et al. (26) that has a similar geographic distribution to the modern reference panel, so as to provide a perspective on how the individuals were clustered without any modern sampling bias (Fig. 2). We did not differentiate individuals from the Iberian Peninsula and those from the Mediterranean region in the penecontemporaneous panel, as the limited coverages of the ancient Iberian individuals can not support constructing a separated population [see Vyas and Koncz et al. (26) for analysis]. We note that the unsupervised ADMIXTURE analysis on the penecontemporaneous reference samples showed similar regional and subregional power to discriminate populations as the 1000G data (25), despite the lower sequence quality of the former (refer to Supplementary Section S4 and *SI Appendix*, Fig. S7 and S8).

The predominant modern genetic components in Collegno as estimated by the supervised fastNGSadmix analysis are Tuscan (TSI, 42%) followed by Central European and Great Britain (CEU+GBR, 39%). We also observed a significant influence of the Iberian component (IBS, 14%). The penecontemporaneous panel exhibited a similar pattern, which is consistent with previous work demonstrating that European population structure has remained largely stable since the Iron Age (27). In the penecontemporaneous panel the Mediterranean European (MEDEU, 55%) and the northern Germany and British (NGBI, 35%) components are predominant (the distinct TSI and IBS components observed in the modern panel are subsumed into the broader MEDEU component). However, we do observe there are some minor differences; for instance, a higher proportion of the Scandinavian/Estonian (SCAND) in the penecontemporaneous panel compared with Finnish (FIN) in the modern panel.

To validate our fastNGSadmix analysis we further employed individual-based qpAdm modeling to assess the genetic ancestry of the individuals in Collegno (Supplementary Section S5, Datasets S3 and S5) and compared the predicted ancestry proportions derived from qpAdm models with those obtained through model-based clustering analysis utilizing the penecontemporaneous panel (*SI Appendix*, Fig. S9). We obtained highly congruent ancestry proportions between the two methods, with a Pearson's product-moment correlation of 0.96 for the Northern European (NGBI plus SCAND) genetic components and of 0.95 for the Southern European (MEDEU) component (*P* < 0.001). The clear concordance can also be observed visually in *SI Appendix*, Fig. S9. Moreover, the findings from qpAdm reinforce our PCA, indicating that the individuals in Collegno exclusively cluster with European populations (except for individual COL_110 which has the lowest coverage and COL_031 which was highly contaminated).

**Fig. 3.**
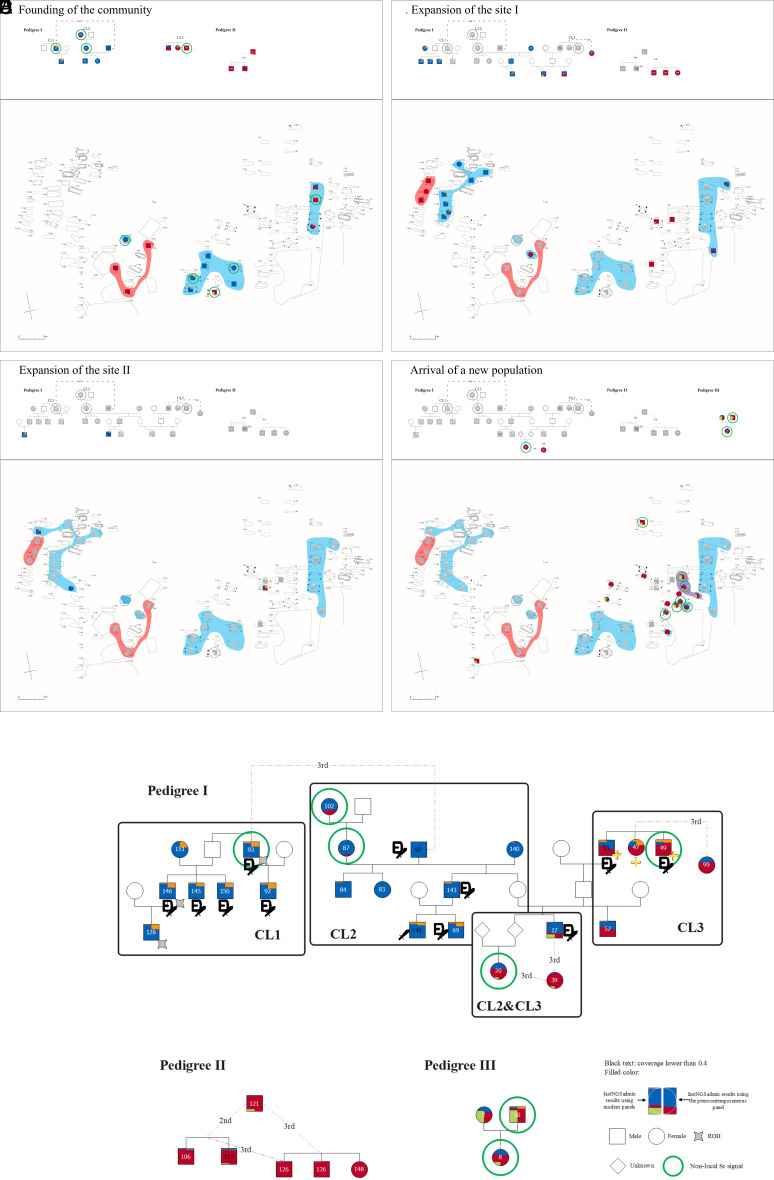
The foundation and development phases and the pedigrees found in the entire community. The phases are represented by figures divided into two subplots (*A*–*D*). The upper subplot depicts the evolution of the pedigrees according to the phases, while the lower subplot marks the location of the connected burials on the cemetery map. Previous phases are shown in gray, while newly appearing individuals are colored based on the results of clustering analyses. The different contours represent the three pedigrees on the cemetery map: Blue: Pedigree I; Red: Pedigree II; Purple: Pedigree III. The cemetery developed from multiple cores in the center and eastern parts (*A*), after the abandonment of its central core an additional core was established to the west and the site expanded to multiple directions (*B*–*C*). In the last phase, substantial reoccupation of the center is observable with new burials on top of the earlier ones (*D*). The pedigrees found in the community are illustrated in the bottom figure (*E*).

We used lcMLkin (28) to assess biological relatedness among individuals in our study and validated the results with READ (29), KIN (30), and ancIBD (31) (Supplementary section S6 and *SI Appendix*, Fig. S10 for details about how the pedigrees were constructed). Based on the 28 newly sequenced individuals and previously published 24 individuals, our analysis combined two previously known biological kindreds into a single expanded pedigree (Pedigree I). Additionally, we extended another pedigree (Pedigree II) and successfully identified a third distinct pedigree (Pedigree III) within the cemetery (Datasets S4, S6, S7, S8, and S9 and Fig. 3*E*).

Pedigree I was particularly extensive, spanning at least five generations and comprising 24 individuals. We further divided Pedigree I into three close-knit groups of biologically related individuals, which we have labeled as CL1, CL2, and CL3. These groups represent direct lineages formed around a male with two reproductive partners (CL2) or sets of siblings (CL1 and CL3) with more distant biological relatedness between them. CL1 and CL2 are related through an unspecified third-degree connection, while CL3 is connected to CL2 through COL_017, whose father belonged to CL2 and mother to CL3. Pedigree II consisted of two groups of siblings showing third-degree relatedness, as well as an additional individual who is related to both sets of siblings. Finally, we identified a small Pedigree III consisting of a parent–offspring trio.

The colors of individuals in the pedigrees (Fig. 3*E* and *SI Appendix*, Fig. S11) indicate fastNGSadmix results generated by both modern (left part) and penecontemporaneous (right part) panels. For Pedigree I CL1 and CL2, individuals have predominantly modern Central European and Great Britain (CEU+GBR) plus Finnish (FIN) genetic ancestry, and contemporary northern Germany and British (NGBI) plus Scandinavian/Estonian (SCAND) genetic ancestry. However, while CL1 and CL2 have similar ancestry proportions based on the modern panel, the penecontemporaneous panel differentiates them, with all individuals in CL1 displaying notable SCAND components (20% to 37%), whereas this is only apparent in the two later-generation individuals (COL_142 and COL_069) in CL2. We also conducted Runs of Homozygosity (ROH) analysis (32) for all 52 individuals (Dataset S10) and identified three individuals (COL_093, COL_146, and COL_128) in CL1 who have ROH segments longer than 4 CM. In contrast, no individuals in other kindreds in Pedigree I or other pedigrees were found to have segments longer than 4 CM.

Individuals in CL3, on the other hand, have mixed genetic components in both the modern (TSI plus CEU+GBR plus FIN) and penecontemporaneous (MEDEU plus NGBI plus SCAND) panels. The three offspring of CL2 and CL3 also show mixed patterns of genetic ancestry.

In Pedigree II, the predominant genetic component is Tuscan (TSI) (82% to 95%) or Mediterranean European (MEDEU) (89% to 100%). In the modern panel, Pedigree III shows predominantly IBS ancestry, and using the penecontemporaneous panel, it shows more Mediterranean European (MEDEU) ancestry. Within the three pedigrees, the IBS component is only present in individuals of Pedigree III and individuals of the later generation of Pedigree I.

It is noteworthy that Pedigree I, particularly CL1 and CL2, display higher proportions of central and northern European ancestry (CEU+GBR plus FIN for the modern panel and NGBI plus SCAND for the penecontemporaneous panel) compared to other individuals in the community (95% and 96% vs. 42% and 44%) and that this ancestry remained mostly unchanged through four generations. These observations were statistically significant using both panels (*P* < 0.0001) based on a permutation test. We also noticed that compared to the individuals in pedigrees, the unrelated individuals at Collegno show greater genetic diversity based on more mixed ancestry profiles (Fig. 2).

We note some minor inconsistencies in estimates of genetic ancestry within pedigrees, in particular lower estimated IBS ancestry in the offspring of Pedigree III compared to their parents, as well as an observation of minor contemporary North African ancestry in COL_106 but sub-Saharan ancestry in their sibling COL_110. It is important to appreciate that all clustering methods such as fastNGSadmix have associated errors in ancestry estimation that will be a function of variables such as data quality (in particular coverage, of which all our samples would be considered low compared to modern DNA) and how closely related the tested source populations are, as well as multimodality during maximum likelihood convergence (which we attempt to mitigate via multiple runs). A simple block bootstrapping procedure using 10 Mb blocks was performed to estimate CI (CIs) for admixture coefficients (Supplementary section S7 and *SI Appendix* Fig. S12 and S13 and *SI Appendix*, Table S1 and S2). In general, the non-European ancestries have very narrow CIs, with the mean SD across samples and ancestries being 0.42% and 0.25% for the penecontemporaneous and modern panels respectively. For European ancestries, the CIs are much larger (mean SD of 6.6% and 8.7% for penecontemporaneous and modern panels respectively). We hypothesized that this was due to switching between ancestries with particularly low genetic differentiation in the panels, as these demonstrated the largest CIs, i.e. IBS (SD = 9.8%) vs TSI (SD = 9.3%) and NGBI (SD = 11.9%) v SCAND (SD = 8.1%). Consistent with this, combining the ancestries for these three pairs of populations results in much narrower CIs (4.4% for IBS_TSI (*SI Appendix*, Fig. S12*c*) versus 5.8% for NGBI_SCAND (*SI Appendix*, Fig. S13*c*)), validating our hypothesis that the similar ancestries are likely prone to ancestry switching across runs. Both cases mentioned above are described in detail in Supplementary section S7).

Compared to two contemporary or slightly earlier sites in Northwest Italy, namely Bardonecchia and Torino-Lavazza (26) (Supplementary Section S8), individuals in Collegno exhibit a higher degree of CEU+GBR plus FIN genetic ancestry, particularly in Pedigree I (Fig. 2 and *SI Appendix*, Fig. S14). This is also validated by the PCA plot where most individuals in Pedigree I clustered with modern northern European populations (*SI Appendix*, Fig. S6). Notably, this pattern is entirely absent in the two penecontemporaneous sites. However, when comparing Collegno to Szólád (13), a penecontemporary site in Hungary associated with an earlier stage of the Langobard migration, it becomes evident that the northern European ancestry is more pronounced in the latter.

### Identity-By-Descent (IBD) Analysis.

We used ancIBD (31) to identify Identity-by-descent (IBD) segments among individuals with greater than 1x coverage from Collegno (n = 29) and available penecontemporaneous samples from the region, Bardonecchia (n = 7) and Torino-Lavazza (n = 4), and the 5th to 6th-century sites of Fonyód (n = 9), Hács (n = 9), Balatonszemes (n = 8) (26) and Szólád (n = 24) (13) from today’s Western Hungary (Datasets S4 and S9). These sites are located in regions that based on the written sources and archaeological record could have been possible sources of the Collegno community. Connections to other penecontemporaneous North Italian sites could indicate local roots, while connections to Western Hungary, the territory of the former Pannonian provinces, might demonstrate the impact of the Langobard migration from Pannonia to Italy in the second half of the 6th century.

**Fig. 4.**
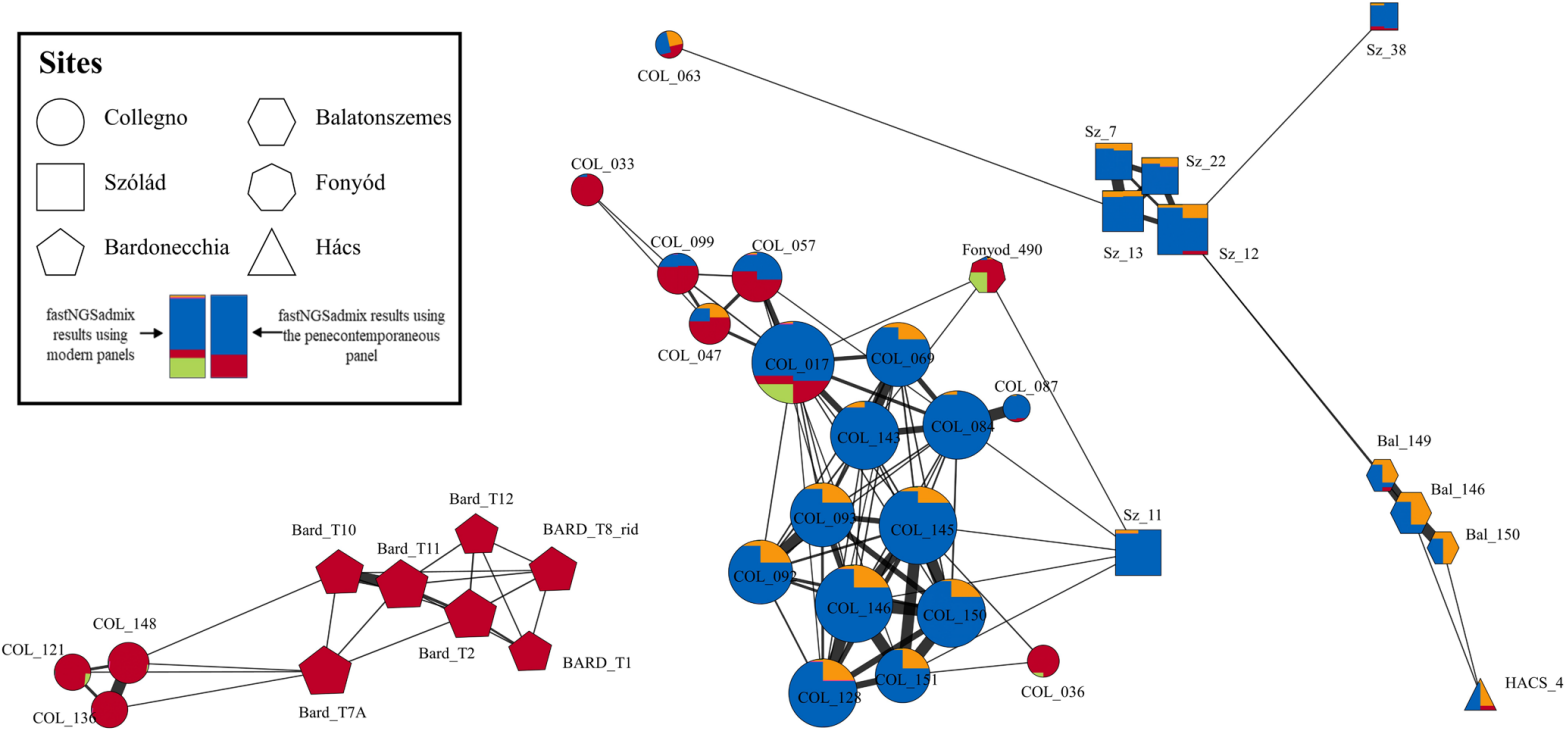
IBD network based on segments longer than 12 cM. Each node in the network represents an individual while each edge represents an IBD connection between two individuals. The edges were weighted by the adjusted Pi-hat value, while the size of the nodes indicates the number of connected edges (degree). The colors of each node indicate the fastNGSadmix results of both the modern and penecontemporaneous panels.

We then constructed a network based on the pairwise IBD sharing (Fig. 4 and *SI Appendix*, Fig. S15). The results verified the pedigrees we constructed. Pedigree I formed a large cluster, and Pedigree II formed another separate cluster. Pedigree III was not included due to the limited coverage. Besides individuals from Pedigree I, the largest cluster also comprised COL_036 and COL_033 (not included in the pedigree as they were not within 3rd degree related to individuals in Pedigree I). This major cluster showed connections to Sz_11 and Fonyod_490. The other cluster consisted of three individuals from Pedigree II linked with two individuals from Bardonecchia. In addition to these clusters, we identified one individual, COL_063, connected to Sz_13, albeit they only shared a single 12 cM segment.

### Radiocarbon Dating.

We produced 14 radiocarbon dates for Collegno (Dataset S11). Sampled individuals were chosen to complement the aDNA analysis. We dated 6 individuals from Pedigree I, including COL_102 and COL_020, the earliest and latest member of CL2, 4 individuals from Pedigree II (COL_110, COL_126, COL_136, COL_148), and 2 individuals from Pedigree III (COL_008, COL_050) to define the relative and absolute chronological position of the pedigrees. The results confirmed the identified biological relatedness and the archaeological dating range of the site with the earliest individual (COL_102) dated between 435 and 591 (95.4% probability) and multiple late individuals dated to the end of the 7th or 8th centuries. We included two additional individuals with relatively high IBS components (COL_035, COL_130) to further date its appearance at the site.

### Strontium isotope (^87^Sr/^86^Sr) Analysis (Supplementary Section S9).

We analyzed the newly collected 28 human samples (Dataset S1) together with the 33 human and 13 environmental samples published in Amorim et al. (13), the latter allowing us to determine the biologically available ^87^Sr/^86^Sr values in the area. We identified a more conservative (wider) and a more strict (narrower) local Sr range (**“**local center**”**), with the former based on environmental samples (this also coincides with the double SD of all human samples) and the latter based on the double SD of the nonadult values, again supported by the available environmental samples. While all nonadult and most adult individuals showed ^87^Sr/^86^Sr values that are consistent with “local center,” we were able to identify four individuals (all females: COL_008, COL_020, COL_035, and COL_147) with values outside of both local ranges and eleven individuals (4 females and 7 males) within the wider range, but outside “local center.” Multiple first- or second-generation individuals of Pedigree I (COL_049, COL_087, COL_093, COL_102) belong to the latter category indicating founding members of the community who were probably nonlocal to the Collegno settlement area, but might have grown up in the wider geographical region. We were also able to identify individuals from later phases with nonlocal signatures (COL_008, COL_020, COL_034, COL_050) indicating that individual and group mobility played an important role in the development of the community even after the initial settlement.

### Carbon (δ^13^C) and Nitrogen (δ^15^N) Isotope Analysis (Supplementary Section S10).

We successfully extracted collagen from 27 of the 28 newly collected human samples (Dataset S1). They were analyzed together with 31 earlier samples published in Amorim et al. (13). Members of the Collegno community consumed both C_3_ and C_4_ plants, as well as animal protein.

Most individuals relied primarily on C_3_ plants and consumed only a small amount of C_4_ plants. However, approximately 22% of the individuals showed elevated δ^13^C values indicative of a more substantial consumption of C_4_ plants, which in Early Medieval Italy would have most likely been millet.

All individuals expressed δ^15^N values indicative of an omnivorous diet. In some cases, high δ^15^N values coincided with high δ^13^C values. This could be explained by the consumption of animals fed with some quantities of C_4_ plants. No significant differences were found between biological sexes. Due to the small sample size, differences between age-at-death categories could not be explored statistically. However, when comparing mean δ^13^C values based on genetic ancestries, the Iberian (IBS) component stood out, as it coincided with significantly lower δ^13^C values (*P* = 0.003). Additionally, members of Pedigree I exhibited significantly higher δ^13^C values compared to the rest of the community (*P* = 0.031). Lastly, we observed similarities in dietary preferences among closely related individuals (first- and second-degree) compared to those who are more distant or unrelated.

## Discussion

### Community Formation and Development at Collegno.

The combination of archaeology, paleogenomics, and isotope analyses described above gave us deep insight into the formation and development of the community that used the Collegno cemetery as a burial site (Fig. 3*A*–*D* and Movie S1). In particular, we were able to identify three main stages: i) foundation of the community, ii) expansion of the site, and iii) arrival of a new population.

i) *Founding of the community*. The earliest members of this funerary community were buried in the central part of the site at the end of the 6th century CE, as suggested by archaeological and radiocarbon dating. This early core formed around two groups of individuals with similar Central-Northern European genetic ancestry who were already related to each other even before their arrival in Collegno (CL1 and CL2) as shown by the biological relatedness identified between COL_093 (CL1) with nonlocal Sr and COL_097 (CL2) (Fig. 3*A*). The high frequency of individuals with nonlocal Sr values, together with their relative early dating in the case of these first-generation members of CL1 and CL2, suggest that these individuals established the cemetery and probably also played a role in the formation of the community using it. Similarities in terms of burial customs—presence of elaborate belt sets and weapons, posthole structure of the graves, etc.,—between CL1 and CL2 also suggest social cohesion, most probably familial relationships between the members of these groups. An unrelated adult male (COL_094), buried in a similarly elaborate grave in this area also shows borderline nonlocal Sr values suggesting that even in this early stage the group might have incorporated individuals without blood relations. IBD connections of Pedigree I (COL_017, COL_0143, COL_057, COL_145, COL_146, COL_151) to predating 5th- and 6th-century Lake Balaton sites in Hungary (Fig. 4) could indicate the foreign origin of this group in the region and fit the historical sources that describe the migration of Langobards from Pannonia to Italy in the late 6th century (6, 8, 33, 34). While biologically not related to Pedigree I, three members of Pedigree II were buried together with them as well. This and their similar radiocarbon dating suggest that they were also members of this early community. Their burials – simple, shallow pit graves, lack of grave goods – are easily distinguishable from the burials of Pedigree I; and their genetic ancestry is also markedly different. Pedigree II also shows multiple IBD connections to individuals from the nearby Bardonecchia site that suggest a differing origin than in case of Pedigree I (Fig. 4). While the community probably formed around Pedigree I, it attracted a wide variety of individuals with different genetic, social, and cultural backgrounds and formed through the coexistence of heterogeneous groups.

**Fig. 5.**
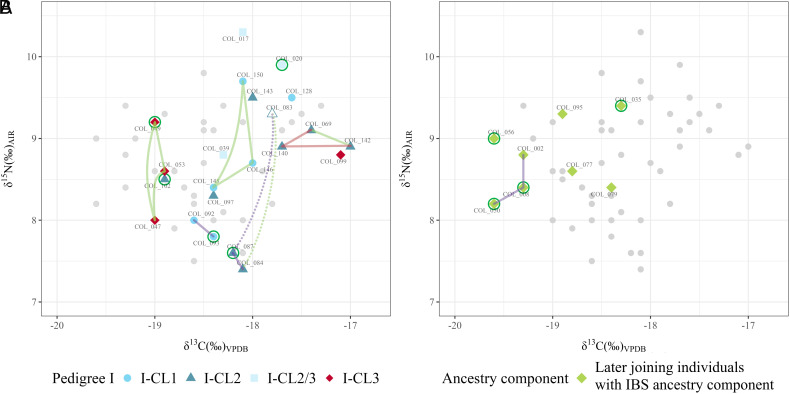
Dietary patterns of individuals at Collegno. The lines connect closely related individuals with similar dietary values (green:siblings, purple:parent–offspring, red:grandparent–grandchildren). Potential nonlocal individuals are marked with dark green circles. (*A*) Symbols represent the clusters of Pedigree I, while gray dots represent the rest of the community. Note: elevated δ^15^N and δ^13^C values of COL_083 (triangle without fill) compared to COL_087 (mother) can potentially be explained by breastfeeding. (*B*) Green symbols represent the late-arriving individuals with a high proportion of the Iberian (IBS) component in the Admixture, while gray dots represent the rest of the community.

Around the same time a second core formed in the eastern part of the site around a north–south row of three siblings (COL_053, COL_049, COL_047) from CL3. Those individuals possess significantly greater proportions of the TSI or MEDEU component compared to individuals in CL1 and CL2. Despite exhibiting notable differences in genetic ancestry, their burials show clear similarities to those of CL1 and CL2 buried with elaborate belt sets and weapons in graves with posthole structures, but some difference is also observable. All siblings were buried with gold folia crosses, an artifact type known in other Langobard-era burials in Italy that is absent in the burials of CL1 and CL2. Their spatial separation also suggests that at the time of the founding of the site, members of CL3 might have maintained a distinct identity. This notion finds further support from the divergent diet of these individuals, as evidenced by their δ^13^C values indicating a primary reliance on C_3_ plants, which contrasts with the more C_4_-based diet observed in CL1 and CL2 (Fig. 5*A*). While COL_049 shows nonlocal Sr value similar to that of COL_093 from CL1, it is impossible to tell whether members of CL3 arrived together with other members of Pedigree I. CL3’s connection to Pedigree I does not predate the formation of the site. It is established during the second generation, when a female member of CL2 and a male member of CL3—probably as the result of intermarriage between the two branches—produced multiple offspring (COL_017 and the unidentified parent of COL_020). Except for the distantly related COL_099, who also has dietary values closer to CL1 and CL2, all identified members of CL3, including the ones also related to CL2, were buried in the same burial group that also expanded to multiple directions in later decades.

ii) *Expansion of the site*. While members of CL3 were buried in the same general area throughout the use of the cemetery, other branches of Pedigree I show a different pattern. The central core was abandoned in the later phases of the site: one branch of CL2 either died out (its latest members are both young children) or left the community, while members of its other branch and CL1 relocated and formed a third core in the western part of the cemetery (Fig. 3 *B*–*C*). The establishment of separate burial groups suggests that the community valued these smaller, close-knit social groups over the extended pedigree, at least in the burial rites, a characteristic observed in some other Italian sites (35). Similarities in dietary patterns of closely related individuals also point to the importance of these close-knit groups. Members of Pedigree II followed the members of CL1, as they continued to bury their dead next to each other, again suggesting a strong social connection between the two groups regardless of their different genetic and archaeological character. Later generation individuals of Pedigree I and II exhibit Sr values that are biologically available in the region and also fall inside the local range indicated by the children. This suggests a stable community; however, the relatively low number of females in Pedigree I (14 adult males and 7 adult females compared to the balanced 16 males and 16 females among genetically sexed adults outside of Pedigree I) and the fact that there is only one single female (COL_087) who has both an identified parent and offspring at the site implies that adult females might have left the community, possibly as a result of exogamy or other social practices.

iii) *Arrival of a new population*. The later phases of the site also include the appearance of individuals with nonlocal Sr values, whose dating is confirmed by their place in the pedigrees (COL_020), radiocarbon dating (COL_050, COL_008), or stratigraphy, i.e. superposition of burials (COL_050, COL_035). The parents-offspring trio of COL_002, COL_050, and COL_008 (Pedigree III) exhibit a significant IBS component, which is not present in the early members of the pedigrees that primarily carry the CEU+GBR and TSI components. This IBS component was present in only one early individual (COL_094). However, it became more prevalent in the last members of Pedigree I (COL_017, COL_020, COL_039). Burials of Pedigree III members (CL_002, CL_008, CL_050) and other unrelated individuals with similar genetic ancestry with a prominent IBS component (COL_034, COL_035, COL_036, COL_056, COL_077, COL_079, COL_095) also exhibit markedly different archaeological character, as they were buried in small, irregular-shaped simple pits without any artifacts in the central part of the site with their graves overlapping with earlier burials on multiple occasions, indicating a later date (also confirmed by the C14 dating of COL_035 and COL_050). Additionally, members of this group exhibit a seemingly different diet that is characterized by significantly lower δ^13^C values indicative of a larger proportion of C_3_ plant consumption when compared to the diet of the established community (Fig. 5*B*). Therefore, they were likely members of a newly arriving group with distinct genetic ancestry that joined the community during the later stages of its existence (Fig. 3*D*) and are contemporaneous with the final members of Pedigree I (COL_020 and COL_039) and its more distant unspecified relatives (beyond 6th degree related) identified only by ancIBD (COL_033 and COL_036). Interestingly this high-impact group-level mobility happens around the final abandonment of the site; both events might have been connected to the community-level realignment that affected the (wider) region at the beginning of the 8th century (36).

### The Role of Elites in Community Formation and Development.

As described previously, we identified a large, biologically related group (Pedigree I) that spans from the founding of the site through at least 5 generations until its abandonment and altogether contains 24 out of the analyzed 52 individuals. Despite differences in genetic ancestry and in dietary preferences between CL1, CL2, and CL3, burials of its members show general similarities in their burial customs but with slight variations. The presence of weapons and elaborate belt sets showed significant correlations with Pedigree I (Cramer's v = 0.86 and 0.79 respectively) (Fig. 2). Different types of weaponry, such as double-edged swords (spatha), single-edged swords (sax), spears and shields, and elaborate multipart belt sets decorated with Animal Style II are generally considered symbols of social and economic power in male burials. These artifact types appear in Italy in the last third of the 6th century, after the Langobard conquest as a clear sign of a new type of elite (6, 8, 9). Most adult males in Pedigree I were buried with these items regardless of their genetic ancestry (12 out of 14 with weapons and belt sets), while these were absent (0 out of 12 adult males) in the burials of analyzed individuals outside of Pedigree I (Fig. 2). Due to the low number of female individuals in Pedigree I, a similar statistical comparison between biological relatedness and burial customs was not possible.

Certain phenomena are only characteristic to specific branches of the pedigree: Gold folia crosses were only found in the burials of the three siblings from CL3 (COL_047, COL_049, COL_053) suggesting a tradition connected to a more closely knit group (Fig. 3*E*). This is further supported by the fact that dietary values of closely related individuals (e.g. the siblings of CL3 or the parent–offspring duo COL_087 and COL_084) tend to cluster closely together, while showing noticeable deviations from those of more distant relatives or biologically unrelated individuals. The line- and branch-specific customs together with the establishment of separate burial groups and nutritional patterns suggest that these branches held meaningful social values, and could probably be interpreted as families or households, the core units of Early Medieval European societies (37). While these units formed around biologically related groups, they probably also incorporated unrelated individuals on legal, spatial, or economic grounds (37, 38). The similar spatial development and dietary patterns of Pedigree I and II may be evidence of this.

The similarities in burial practices among the different branches of Pedigree I, regardless of their genetic ancestries (particularly between CL1 to CL2 and CL3), suggest that burials were not only used to maintain social cohesion within the group, but also to exclude unrelated members and distinguish between "us and them." The paleodietary analysis provided further support to this theory, as it revealed statistically significant differences in mean δ^13^C values between members of Pedigree I and the rest of the community (*P* = 0.031) (Fig. 5*A*). This suggests that those individuals did not share meals with other members of the community on a regular basis and might have actively chosen to incorporate different dietary resources compared to the rest of the community. The absence of posthole structures, weapons, and complex belt sets outside of Pedigree I further underscores the exclusive nature of these burials. Interestingly, these similarities predate the connection between CL2 and CL3. The presence of prestigious artifacts suggests that the “founding families” held elevated economic and/or social status within the community (8, 39), a status that their descendants retained throughout multiple generations, as evidenced by the continuous burial of males with these artifacts until the very end of the pedigree (Fig. 3*E*) (40), although certain differences are observable due to changes over time. It points toward a stable hierarchy where social status was inherited, rather than prestige-based. The lower number or lack of grave goods in the burials of the latest members is probably not connected to a change of their economical and social standings, but is the result of a general trend: grave goods started to disappear from burials by the middle of the 7th century in whole Western Europe (15). The site’s abandonment in the early decades of the 8th century, not long after the last burial of a Pedigree I member (COL_020, who showed nonlocal Sr), might have been the result of either the relocation or extinction of these leading families or change of the location of the burial site in favor of a nearby churchyard as part of a general trend from the 8th century onward (41, 42).

There are burials and settlement structures at or near the site of Collegno clearly dated to the first half/middle of the 6th century, to the period of the Ostrogothic Kingdom. Members of Pedigree I appear to represent an emerging elite that founded a new community after the Langobard occupation of Northern Italy in the second half of the 6th century that replaced the earlier one. The genetic results show that blood relations could play an important role in the formation of social kinship among these newly emerging elites identified by their burial customs during the early stages of the Langobard Kingdom, but they cannot be necessarily understood as genetically homogeneous groups. Members of CL1 and CL2 had predominantly central-northern European genetic ancestry that is absent in available predating and penecontemporaneous sites from the region (Bardonecchia and Torino-Lavazza, *SI Appendix*, Fig. S6) and might be the result of a possible migration. This possibility is reinforced by the IBD connections to Pannonia, a territory that according to the historical sources, served as the starting point of the Langobard’s migration to Italy. On the other hand, CL3 shows genetic ancestry that is similar to the other penecontemporaneous sites in close geographical proximity and lacks long-distance IBD connections (Fig. 4). However, these interpretations are heavily limited by the low resolution of the dataset; additional samples from penecontemporaneous sites from the neighboring regions would be required to understand the real importance of these connections. At this point, it is impossible to tell whether this genetic heterogeneity was already established before the arrival to Italy, fitting to the long list of ethnic names connected to Alboin’s migration in the written sources; or is the result of regional processes through the assimilation of local elites that embraced the customs of the newcomers as suggested by the archaeological record. Nevertheless, the elites of these communities, by occupying strategically important locations, played a very important role in exercising power and maintaining political control in the rural areas in an otherwise town-based regnum (8, 9).

### Conclusions.

Our results shed light on the complexity of a new community that formed during a transitional period, when many Roman structures were dissolving, and a new post-Roman polity, the Langobard Kingdom, was consolidating in Italy during the late 6th-early 7th century CE. The Collegno community was established by and organized around a network of biologically and socially related individuals, most probably multiple elite families through which the Langobard Kingdom exercised and maintained its power in rural regions after the conquest of Italy (6, 8, 10). These families showed similarities in both their burial customs and dietary patterns suggesting a similar, if not identical social standing, but the pedigree included individuals of vastly different genetic ancestries, with the majority of them having Central-Northern European genetic components which are predominantly found in significant proportions within this group. Multiple members of this pedigree showed distant genetic connections to individuals from predating sites in Western Hungary, the starting point of the migration of the Langobards to Italy (33, 34). Over time these groups developed into a single extended pedigree, while simultaneously maintaining some form of separation in their burial customs and lifestyle from the rest of the community. The presence and exclusivity of weapons and elaborate belt sets in their burials as well as a different diet imply that they possessed an elevated social status and bequeathed it to their descendants, suggesting a society where status inheritance was established as described in the written sources (9, 43). Similarly to the elite, the community also included individuals of various genetic ancestries and maintained its heterogeneity through the integration of newly arriving individuals and groups, as mobility played an important role in community development at various stages. The results of this Collegno case study show how shifts in political power and migration impacted the formation and development of a small rural community in one of the core territories of the former Roman Empire after its dissolution and during the emergence of a new kingdom. Moreover, it shows that Early Medieval elites were able to integrate individuals from heterogeneous backgrounds and that these elites were the result of (political) agency rather than belonging to biologically homogeneous groups. This image accords well with Paulus Diaconus’s depiction of the heterogeneity of King Alboin’s migrating following.

## Methods

### Data Generation and Bioinformatic Processing.

Petrous bone samples were collected for all the individuals with the exception of COL_142, COL_017, COL_150, and COL_069 for which auditory ossicles samples were collected (44). All the samples were then powdered in ancient DNA clean rooms at the Department of Biology, Laboratory of Molecular Anthropology and Paleogenetics, University of Florence. For the library preparation, 30 uL of extract was used, employing partial UDG treatment (45). DNA extraction was carried out using a silica-based protocol (46) and then underwent an in-solution capture targeting ~1.24 M SNPs at the Max Planck Institute for the Science of Human History (MPI-SHH) in Jena, Germany. For more details, refer to Supplementary Section S2.

The raw sequences underwent processing following a published pipeline (47). This involved trimming and merging reads, which were then mapped to GRCh37 using samtools (48). Duplicate reads were marked using Picard Tools (49), and reads shorter than 30 bp were filtered out. Untrimmed reads were excluded from further processing and analysis, as they were more likely to contain contaminant sequences. To assess postmortem DNA damage patterns, we employed mapDamage 0.3.3 (50).

For the newly sampled 28 individuals, BAM files were called using an indent caller that disregarded the first and last five bases of each read, accomplished using in-house scripts (www.github.com/kveeramah). The output VCF files contained diploid genotypes and genotype likelihoods. Coverage for the autosomal 1240 K SNPs, as well as the mitochondrial genome, was calculated for all individuals using gatk v4.2 DepthOfCoverage (49). The number of autosomal 1240 K SNPs covered by at least one read with mapping and base quality scores greater than or equal to 30 was calculated for each BAM file using samtools depth (48).

To determine the genetic sex of individuals, the Sex.DetERRmine pipeline was employed, which evaluated relative X and Y chromosome coverage (https://github.com/TCLamnidis/Sex.DetERRmine). Angsd was subsequently used to estimate nuclear contamination rates in genetic males based on the hemizygous X chromosome (51). Additionally, mitochondrial contamination rates for all individuals were estimated using Schmutzi (52) (Dataset S1).

### Mitochondrial and Y Chromosome Analyses.

To analyze the mitochondrial DNA, the BAM files of the 28 new individuals were filtered to retain only the reads aligning to the mitochondrial genome, which were then converted to fastq files using samtools (48). Mitochondrial genomes from individuals with more than 1,500 mitochondrial reads were assembled using the Mapping Iterative Assembler (53), a specialized tool for ancient mitochondrial genome assembly. Subsequently, haplotype assignment was performed using MitoTool 1.1.2 (54) to determine the mitochondrial haplogroups.

Y chromosome analysis was conducted using the 1,240 K Y chromosome SNPs. The vcf files were aligned in a Microsoft Excel spreadsheet and the phylogenetic position of each Y chromosome SNP was determined using information from published databases (https://isogg.org/, accessed on 08/31/2023) (25, 55–57), which allowed for the identification of NRY haplogroups, named according to the updated ISOGG (International Society of Genetic Genealogy) nomenclature. Previously unidentified SNPs were assigned to their respective haplogroup following a cladistic approach, considering the nonrecombining nature of NRY. The singletons, unless already present in the above public databases, were not considered.

### Pseudohaploid PCA Using Two Modern Reference Datasets.

We conducted principal component analysis (PCA) by converting diploid 1,240 K VCF files from Collegno, Bardonecchia, and Torino-Lavazza to pseudohaploid plink datasets using a custom, in-house script. Two datasets, Affymetrix Human Origins array data from the Allen Ancient DNA Resource v50.0 (16–22) (Dataset S2) and imputed POPRES (23) datasets were used as references. We used smartPCA (58, 59) to perform PCA on the ancient individuals with the two reference datasets via a procrustes transformation (https://github.com/ShyamieG/, Supplementary section S3 for detailed information).

### Modeling Genetic Ancestry of Collegno Using qpAdm.

We employed qpAdm (60) to investigate the genetic ancestry of 52 individuals and compare the results obtained through model-based clustering analysis. A random read-indent caller that excluded the first and last eight bases of each read (https://github.com/kveeramah/) was used in the analysis for all the target, source, and reference individuals. For reference populations (right populations) used in the analysis, we utilized 72 prehistoric individuals of Anatolian_Neolithic (EEF, n = 26), Steppe_Eneolithic (SA, n = 18), Western Hunter-Gatherer (WHG, n = 15), Iran_Neolithic (Iran_N, n = 9), and Morocco_Iberomaurusian (MIM, n = 4) origin (Dataset S3) (16, 17, 60–69). The source populations were chosen from our contemporaneous reference populations, as indicated in the fastNGSadmix panels (Dataset S3). The one-source model was initially implemented, followed by a stepwise inclusion of additional source populations. The process concluded when the tail probability exceeded 0.05. (Supplementary section S5 for more detailed information and Dataset S5 for results)

### Biological Relatedness Assessment.

To detect close biological relatedness among our new set of 28 individuals as well as 24 previously published individuals, we employed the lcMLkin software package (28). All individuals were included in the analysis, irrespective of their level of coverage. A customized version of lcMLkin was utilized, which incorporated external allele frequency data, following a similar approach as Amorim et al. (13).

The analysis was conducted using genotype likelihood data from 1,076,758 autosomal 1240 K sites as input. To enhance the accuracy of the results, allele frequency data from the 1000 Genomes CEU and TSI populations were used, along with a merged dataset of the two populations, CEU_TSI. Relationships between low-coverage individuals with minimal common SNP coverage (i.e., less than 10,000 shared SNPs in an lcMLkin analysis) were disregarded, as such relationships were likely to be spurious and not indicative of genuine biological relatedness.

In order to validate the lcMLkin results, we also conducted analysis using READ (29), KIN (30), and ancIBD (31). The READ analysis was conducted on 164,127 transversions with minor allele frequencies above 0.05 in the combined CEU+TSI population. Pseudohaploid genotypes were employed for this purpose. The KIN analysis was conducted following the pipeline in https://github.com/DivyaratanPopli/Kinship_Inference. We used the default parameters and the -cnt parameter was set to 0 in this analysis. A detailed description of how results generated by KIN and READ were compared with lcMLkin can be found in Supplementary section S6, and Datasets S6, S7 and S8.

### Model-Based Clustering Analyses.

We conducted model-based (supervised) clustering analyses using fastNGSadmix (24). We used two reference panels. The first one was a seven-reference panel based on imputed ancient penecontemporaneous individuals representing Mediterranean Europe (MEDEU), Northern Germany/Britain (NGBI), Scandinavia/Estonia (SCAND), East Asia (EASIA), South Asia (SASIA), North Africa (NAFRICA), and sub-Saharan Africa (SUBSAHARAN) (26). We also conducted analyses using seven modern 1000 Genomes Project populations, namely CEU+GBR, FIN, IBS, TSI, YRI, EAS, and SAS. We merged the CEU and GBR populations into a single population, as the two populations are not properly distinguishable by these types of analyses (13).

Beagle PL files containing phred-scaled genotype likelihoods were generated using vcftools (70) for all individuals at 1,076,939 autosomal sites for use by fastNGSadmix. The analysis was run separately for each individual using default parameters 50 times with random seeds, and the run with the highest likelihood chosen as the final result. The admixture plots shown in Fig. 2 utilize the point estimates given by the best run.

To estimate CI for individual admixture coefficients, we implemented a simple (i.e. blocks are nonoverlapping) block bootstrapping procedure (71), resampling 10 Mb chromosome blocks with replacement (Supplementary section S7 and *SI Appendix*, Fig. S12 and S13 and *SI Appendix*, TableS1 and S2).

### Identity-By-Descent (IBD) Analysis Using ancIBD.

Analysis with ancIBD was restricted to all individuals with > = 1× coverage from Collegno (n = 29), Szólád (n = 24) Fonyód (n = 9), Balatonszemes (n = 8), Hács (n = 9), Bardonecchia (n = 7), and Torino-Lavazza (n = 4). All individuals were called for genotypes from the whole genome, imputed for all diploid, dinucleotide sites within the 1000 Genomes Project Phase 3 v5a VCF files using GLIMPSE v1.1(72) (using the 1000 Genomes Project data as the imputation reference) (25), and filtered down to the 1,240 K positions. We then followed the steps described in https://github.com/hringbauer/ancIBD to convert VCF files to HDF5 format, and called the IBD segments using the default parameters (except for the “p _ co”l which we used “variants/AF_ALL” that encodes the allele frequencies calculated from the HDF5 instead of “variants/RAF” in the default setting). We only retained segments that were longer than 12 cM and the Pi-hat value was calculated by dividing the sum_IBD by the total CM of the genome. As ancIBD cannot differentiate IBD2 with IBD1, we adjusted the Pi-hat value for known siblings to 0.5.

We used the ancIBD results to construct an undirected network using the ForcedAtlas2 algorithm (73) with Gephi v.0.9.7 (74). Each node in the network represents an individual while each edge represents an IBD connection between two individuals. The edges were weighted by the adjusted Pi-hat value. Due to the large relative differences between the adjusted PI-hat values (they ranged between 0.001838 to 0.5) the edge weights were adjusted to a 0 to 1 scale. During the creation of the network, individuals without any IBD connection (degree = 0) were filtered out. The final network contained 43 nodes and 107 edges with an average degree of (number of connected edges per node) 5.08.

### Radiocarbon Dating (^14^C analysis).

Bone samples (Dataset S11) were physically and chemically pretreated at the Radiocarbon Laboratory of the Max Planck Institute of Geoanthropology. Collagen was extracted from approximately 0.5 to 1 g of bone material using an acid–base–acid protocol followed by filtration and freeze-drying adapted from the Longin method (75). Collagen samples were combusted and graphitized at the ^14^C-Analysis Laboratory of the Max Planck Institute of Biogeochemistry, following established procedure (76). Measurements were carried out using a MICADAS AMS system from Ionplus (Switzerland) (77). Data analysis was as described in Steinhof (78).

### Strontium (^87^Sr/^86^Sr) Isotope Analysis.

The strontium isotope analyses were conducted in the Isotope Laboratory of the Adam Mickiewicz University at Poznań, Poland. Before analysis, the mechanically isolated enamel was cleaned in an ultrasonic bath in ultrapure water to remove the sediment particles. Afterward, about 10 mg of powdered enamel was treated sequentially with 0.1 ultrapure acetic acid (5 times) to eliminate the diagenetic Sr contamination, according to the procedure described by Dufour et al. (79). Subsequently, the powdered samples were dissolved overnight on a hot plate at temperature c. 100 °C in closed PFA vials using a mixture of concentrated hydrofluoric and nitric acid (4:1). Strontium was separated using a miniaturized chromatographic technique described in Pin et al. (80), with modification by Dopieralska (81). Next, it was loaded with tantalum pentachloride activator on a single Re filament and analyzed in dynamic collection mode on a Finnigan MAT 261 thermal ionization mass spectrometer.

Samples were measured along with the NBS 987 Sr standard (mean ^87^Sr/^86^Sr value of ten analyses was 0.710238, with 2σ of 0.000010). The measurements were corrected to ^86^Sr/^88^Sr = 0.1194, and the results were normalized to certified values of NIST-987 = 0.710340.

### δ^13^C & δ^15^N Isotope Analysis.

Collagen was extracted from human bones following the standard protocol of the Department of Bioarchaeology, University of Warsaw, based on the method detailed by Longin (75) and modified by Brown et al. (82). Bone pieces weighing 400 to 600 mg were manually abraded, demineralized in 0.3 M aq. HCl at room temperature, and then gelatinized in hydrochloric acid solution (pH 3) at 70 °C for 48 h. After filtration using Ezee Filter separators and freezing, the samples were freeze-dried.

The ^13^C/^12^C and ^15^N/^14^N ratios of the samples were measured at the Environmental Isotope Lab, Geosciences, University of Arizona, using a continuous-flow gas-ratio mass spectrometer (Finnigan Delta Plus XP) coupled with an elemental analyzer (Costech) (Supplementary section S10).

For further analysis, only samples with carbon concentration greater than 13%, nitrogen concentration greater than 4.8%, and atomic C/N ratio between 2.9-3.6 were accepted (83–85).

### Runs Of Homozygosity (ROH) Analysis.

We ran hapROH (32) to estimate the ROH of all the 52 individuals in Collegno. Convertf (https://github.com/argriffing/eigensoft/tree/master/CONVERTF) was used to convert Collegno pseudohaploid plink files into eigenstrat files. For the reference dataset, we used the default reference data from the global 1000 Genome data (n = 5008 haplotypes from 2504 individuals), filtered down to biallelic 1240 K SNPs. We used the setting “min_cm = [4, 8, 12, 20], snp_cm = 50, gap = 0.5, min_len1 = 1.0, min_len2 = 4.0” and counted all the ROH blocks longer than 4 cM in Dataset S10.

## Supplementary Material

Appendix 01 (PDF)

Dataset S01 (XLSX)

Dataset S02 (XLSX)

Dataset S03 (XLSX)

Dataset S04 (XLSX)

Dataset S05 (XLSX)

Dataset S06 (XLSX)

Dataset S07 (XLSX)

Dataset S08 (XLSX)

Dataset S09 (XLSX)

Dataset S10 (XLSX)

Dataset S11 (XLSX)

Movie S1. An animation showing the development of Collegno over time. The use of the site was divided into four temporal phases using a combination of archaeological chronology, radiocarbon dating, osteological and genetic information. Newly appearing individuals are colored based on the results of genetic clustering analyses, while earlier phases are shown in gray. The different colors represent the three pedigrees on the cemetery map: Blue: Pedigree I; Red: Pedigree II; Purple: Pedigree III. The cemetery developed from multiple cores in the center and eastern sections (a). After the abandonment of its central core an additional core was established to the west and the site expanded to multiple directions (b–c). In the last phase substantial reoccupation of the center is observable with new burials on top of the earlier ones (d)

## Data Availability

Our newly generated sequence data from 28 individuals are available from the NCBI Sequence Read Archive (SRA) database under accession PRJNA1023512 (86). We accessed the POPRES (Population Reference Sample) dataset collected and published by Nelson et al. (23) from dbGaP (accession phs000145.v4.p2).
