# Supplementary material for: The role of emerging elites in the formation and development of communities after the fall of the Roman Empire
Source: Proc Natl Acad Sci U S A. 2024 Aug 19;121(36):e2317868121. doi: 10.1073/pnas.2317868121 (PMC11388374; doi:10.1073/pnas.2317868121)
Supplement: Supplementary file 1 — Appendix 01 (PDF) [file pnas.2317868121.sapp.pdf]

Supporting Information

The role of emerging elites in the formation and development of communities after the fall of the Roman Empire

Yijie Tian[a] *, István Koncz[b] *, Sarah Defant[c d e] *, Caterina Giostra[f], Deven N. Vyas[a] , Arkadiusz Sołtysiak[g], Luisella Pejrani Baricco[h], Rafał Fetner[g], Cosimo Posth[i], Guido Brandt[j], Elena Bedini†[f], Alessandra Modi[k], Martina Lari[k], Stefania Vai[k], Paolo Francalacci[l], Ricardo Fernandes[d g m n], Axel Steinhof[o], Walter Pohl[p], David Caramelli[k], Johannes Krause[j], Adam Izdebski[d q], Patrick J. Geary[r +] and Krishna R. Veeramah[a +]

[a] Department of Ecology and Evolution, Stony Brook University, Stony Brook, 11794, NY, USA
[b] Institute of Archaeological Sciences, ELTE - Eötvös Loránd University, Budapest, 1088, Hungary
[c] Institute of Prehistoric Archaeology, Freie Universität Berlin, Fabeckstr. 23/25, 14195 Berlin, Germany
[d] Max Planck Institute of Geoanthropology, Kahlaische Straße 10, 07743 Jena, Germany
[e] Institute of Greek and Latin Languages and Literatures, Freie Universität Berlin, Habelschwerdter Allee 45, 14195 Berlin, Germany
[f] Department of History, Archaeology and Art History, Catholic University Milan, Italy
[g] Department of Bioarchaeology, Faculty of Archaeology, University of Warsaw, ul. Krakowskie Przedmieście 26/28, 00-927 Warszawa, Poland
[h] Soprintendenza Archeologia, Belle Arti e Paesaggio per la città metropolitana di Torino, Piazza San Giovanni, 2, 10122 Torino TO, Italy
[i] Archaeo- and Palaeogenetics, Institute for Archaeological Sciences, Department of Geosciences, University of Tübingen, Tübingen 72074, Germany
[j] Department of Archaeogenetics, Max Planck Institute for Evolutionary Anthropology, Leipzig, 04103, Germany
[k] Department of Biology, University of Florence, Via del Proconsolo 12-50122 Firenze, Italy
[l] Dipartimento di Scienze della Vita e dell'Ambiente, Università di Cagliari, Cagliari, 09126, Italy
[m] Arne Faculty of Arts, Masaryk University, Nováka 1, 602 00, Brno-střed, Czech Republic
[n] Climate Change and History Research Initiative, Princeton University, Princeton, USA
[o] Max Planck Institute for Biogeochemistry, Hans-Knöll-Straße 10, 07745 Jena, Germany
[p] Institute for Medieval Research, Austrian Academy of Sciences; Institute for Austrian Historical Research, University of Vienna, Hollandstrasse 11-13/3, 1020 Vienna, Austria
[q] Institute of History, Jagiellonian University in Krakow, ul. Gołębia 24, 31-007 Kraków, Poland
[r] School of Historical Studies, Institute for Advanced Study, 1 Einstein Drive, Princeton, New Jersey, 08540 USA

*These authors contributed equally to this work
+Corresponding authors (geary@ias.edu, krishna.veeramah@stonybrook.edu)

This PDF file includes:

Supporting text
Figures S1 to S24
Tables S1 to S9
SI References

Other supporting materials for this manuscript include the following:

Movie S1
Datasets S1 to S11

Supporting Information Text

S1. Demographic information of the Collegno cemetery

István Koncz

The osteological age and sex determination was conducted by Elena Bedni and Francesca Bertoldi.(1, 2) The various analyses in the paper are based on the osteological age determination and partially the osteological sex determination, in case of genetically analyzed individuals we used the genetic sex estimation. Age categories were given as follows: infans1 (0-6 years), infans2 (7-14), juvenis (15-19), adultus1 (20-29), adultus2 (30-39), maturus1 (40-49), maturus2 (50-59), senilis (60-). In certain cases due to bad preservation or insufficient data broader categories were also used. During the analyses genetic sex was disregarded in case of nonadult individuals (infans1-2) and we compared three main groups: adult females, adult males and nonadults.

The 157 graves that can be dated to the Langobard period at Collegno contained 149 human individuals and a horse, while 7 graves were without any observable osteological remains. Among the 149 human remains there were 65 adult males and 40 adult females with an additional 14 adult individuals where sex determination was impossible. We observed discrepancy between osteological and genetic sex estimation in seven cases among adults, six of them were genetic females described as osteological males. These differences could be the result of the generally gracile stature of the individuals (2). 30 children were buried in the cemetery.

Life expectancy at birth in the Collegno cemetery shows slight differences between males and females. At young adult age (ca. 20-30 years) the life expectancy of males is higher, but after young adult age (30-) the females tend to live slightly longer (Fig. S2). Life expectancy at age 30 for males: 20.875 years; for females: 21.494. Mortality curves show that this difference is the result of more females dying as young adults (probably connected to child birth): 11 (27.5% of all adult females) in juvenis and adultus1 age categories compared to the 8 males (12.3% of all adult males) (Fig. S3).

S2. Collegno sample collection, library preparation and sequencing

Alessandra Modi and Guido Brandt

Double-stranded (3) libraries from Collegno were sequenced in two separate batches in January, 2020 (n=20) and November, 2020 (n=8). All sequencing was performed at Max-Planck-Institute for Evolutionary Anthropology (MPI-EVA), Leipzig, Germany. All 28 libraries underwent 1240K capture sequencing for 1.24 million SNPs. Libraries with ≥ 0.1% percentage of ancient human DNA in shotgun screening data were enriched in-solution for 1,237,207 targeted single nucleotide polymorphisms (‘1240k capture’) across the human genome (4) . A single round of enrichment was performed. Enriched libraries were pooled equimolarity and sequenced on Illumina Hiseq4000 NGS sequencer using 75bp single-end reads.

S3. Pseudohaploid PCA using two modern reference datasets

Yijie Tian

We conducted principal component analysis (PCA) comparing the individuals from Collegno against reference populations. For this analysis, we converted our diploid 1240K VCF files to pseudohaploid Plink datasets using a custom, in-house script. Heterozygous genotype calls were converted to calls for the allele with greater allele depth; when both alleles had the same depth, an allele was chosen at random.

Two different modern genotype datasets were used as references. The first one used modern Eurasian populations from Affymetrix Human Origins array data found in the Allen Ancient DNA Resource v50.0 (5–12), Another one used modern European populations from the POPRES dataset (13) in its imputed form from Veeramah et al.(14). All analyzed Affymetrix Human Origins array data as well as POPRES genotypes were also made pseudohaploid by randomly choosing one allele for all heterozygous genotypes. We used the overlapping SNPs to plot the data (537,207 SNPs and 328,687 SNPs respectively).

We used an automated script to use smartPCA (15, 16) to perform PCA on the two reference datasets including one ancient individual at a time (https://github.com/ShyamieG/). We then used an in-house script to conduct a Procrustes transformation, merging all 52 ancient individuals (with more than 10,000 SNPs) onto a single principal component analysis (Fig.S4, S5). In our PCA plots of PC1 and PC2 for these analyses, we divide Affymetrix Human Origins populations into nine regions (i.e., Europe, Caucasus, Middle East, Central Asia, Siberia, Siberia (Far East), South Asia, East Asia and Southeast Asia) and divide POPRES populations into regions (i.e., CE, EE, NE, NEE, NWE, SE, SEE, and WE) as demarcated in Veeramah et al (14).

To compare the genetic diversity between Collegno and two penecontemporaneous sites (Bardonecchia and Torino-Lavazza), we also performed another PCA using all the ancient samples from the three sites against POPRES dataset (Fig. S6). The individuals in Bardonecchia and Torino-Lavazza underwent the identical processing procedure as those in Collegno. We utilized all 328,687 overlapping SNPs to generate the data visualization.

S4. Unsupervised clustering analysis of penecontemporaneous samples

Deven N. Vyas, Yijie Tian

To validate the penecontemporaneous reference panel, we conducted unsupervised ADMIXTURE analyses (17). For these analyses, we used the exact same imputed genotypes from Vyas, Koncz, et al. (18) from the 181 reference individuals. We initially conducted an analysis using K=7 to match the number of reference populations (Fig. S7). We found that the four non-European reference panels (EASIA, SASIA, NAFRICA, and SUBSAHARAN) were each dominated by one component. Among the three European panels (MEDEU, NGBI, and SCAND), SCAND was dominated by one component (orange) and MEDEU by another (red), with NGBI be intermediate between the two (though more orange than red). MEDEU and NGBI also contain a third purple component found at lower proportions. We suspect that this component reflects more noise than signal (i.e., too many components given the population structure of the data)

We subsequently performed an analysis using K=6 (Fig. S8). The result revealed more clear patterns within European populations, with SCAND predominantly characterized by an orange component, MEDEU by an red, and NGBI appeared to exhibit an intermediate

composition between the two. Although we still observed subtle influences of the light and dark blue components on MEDEU, these did not account for significant proportions compared to the purple component when K=7. Consequently, we contend that the model with K=6 is likely the most suitable fit, considering the resolution of our data quality.

S5. Modeling genetic ancestry of Collegno using qpAdm

Yijie Tian

To validate both our PCA and model-based clustering analyses, we employed qpAdm (19) analysis integrated within AdmixTool (9). This approach allowed us to offer a different perspective on how individuals from Collegno can be modeled in relation to penecontemporaneous populations. Pseudohaploid genotype data were utilized for this purpose. Given the sensitivity of qpAdm to coverage differences, our genotype calling strategy differed from that used in the PCA. For PCA preparation, we prioritized the allele with higher allele depth in heterozygous loci. However, for qpAdm analysis we employed a random read-indent caller that excluded the first and last eight bases of each read (https://github.com/kveeramah/). In addition, genotypes were randomly called at heterozygous loci, ignoring the associated depths, following the method outlined at (https://github.com/DReichLab/adna-workflow).

For reference populations (right populations) for our analysis, we utilized 72 prehistoric individuals of Anatolian_Neolithic (EEF, n=26), Steppe_Eneolithic (SA, n=18), Western Hunter-Gatherer (WHG, n=15), Iran_Neolithic (Iran_N, n=9), and Morocco_Iberomaurusian (MIM, n=4) origin (see Dataset S3)(5, 6, 19–28). These individuals were selected from the collective sets used in previous prehistoric analyses by Amorim et al (29). and Antonio et al (30). The BAM files of these individuals were sourced from published data, and we conducted genotyping for the autosomal 1240K SNPs, aligning with the methods utilized for other individuals in our study.

Source populations were chosen from our contemporaneous reference populations, as indicated in the fastNGSadmix panels (see Dataset S3). The qpAdm modeling requires the independence of source populations and the lower coverage Spanish samples may introduce significant noise into the MEDEU panel, so we excluded them (n=14) and utilized only the Italian samples (n=26). To maintain balanced sample sizes across the other two large source populations, we randomly excluded 14 individuals from each of the NGBI and SCAND populations. Consequently, the final sample sizes for source populations were as follows: n=24 for MEDEU, NGBI, and SCAND; n=16 for EASIA; n=17 for SASIA; n=20 for NAFRICA; and n=7 for SUBSAHARAN (see Dataset S3).

In the testing process, we started with the one-source model, evaluating each of the seven source populations independently for 52 Collegno individuals. In each test, if the tail probability exceeded 0.05, we deemed the model acceptable, concluding that the target individual cannot be distinguished from the source population. In such cases, the assigned ancestry proportion for the source population was set to 1. If the one-source model proved inadequate, we gradually added other source populations and stopped when the tail probability surpassed 0.05. If the model didn't generate a tail probability higher than 0.05 even after testing various combinations of source populations, we then retained the specific model that yielded the highest tail probability.

The results (refer to Dataset S5) revealed that 37 individuals could be effectively modeled using either one-source or two-sources models, as indicated by a tail probability greater than

0.01 . This modeling included the incorporation of NGBI and/or MEDEU populations. However, for the remaining 15 individuals, seven still exhibited relatively high tail probabilities (> 0.001) when using MEDEU, NGBI, and NAFRICA as source populations. The remaining eight individuals, which could not be well-fitted displayed high southern European ancestry in the model-based clustering analysis, ranging from 0.67 to 0.99 MEDEU ancestry. Notably, four of these individuals belong to Pedigree II. We attribute this challenge to the absence of a suitable proxy for some samples with southern ancestry, highlighting a limitation in the available data produced to date from the historical era.

Nevertheless, the individual COL_126 in Pedigree II can be effectively modeled using a one-source model with the MEDEU population ($p = 0.03$). This observation suggests a significant influence of MEDEU ancestry within this pedigree, which corresponds well with the results of the model-based clustering analysis (Fig. 2).

Additionally, we observed limitations in the qpAdm modeling's ability to effectively differentiate between the NGBI and SCAND populations. This discrepancy could stem from a lack of distinguishing power within the reference (right) populations for these specific groups. Given that qpAdm operates on the hypothesis that source populations can be distinguished by reference populations, its efficacy in our analysis appears to be compromised. Alternatively, the use of fastNGSadmix with these source populations might offer enhanced capabilities for inferring ancestries in such datasets. In our analysis, we opted for NGBI when the model proved effective for both NGBI and SCAND populations.

Nevertheless, our conclusion that individuals in Collegno cluster with European populations are reliable considering highly congruent ancestry proportions of model-based clustering analysis and qpAdm analysis. The results from these two methods are visually presented together for each individual in Fig. S9. In cases where individuals could not be effectively modeled, we selected the model with the highest tail probability (see Dataset S5). The combined results of the two methods are graphically presented side by side, with each individual separated by a blank column. This visual depiction highlights a strong concordance between all the qpAdm models and model-based clustering analyses, with a notable Pearson's product-moment correlation of 0.96 for the Northern European (NGBI plus SCAND) genetic components and of 0.95 for the Southern European (MEDEU) component.

S6. Construction of Collegno pedigrees

Yijie Tian

In order to examine biological kinship at Collengo, we used the pairwise relatedness results generated by lcMLkin (31) (using external CEU+GBR allele frequencies) as the initial source of information, and validated these with KIN (32), READ (33) and (where possible given a coverage limit of 1x) ancIBD (34) in order to reconstruct and refine the eventual pedigrees (Dataset S6, S7, S8 and S9). We also integrated information from mtDNA, Y chromosome haplogroups, stylistic elements of the grave good (Dataset S1) as well as C14 dating (Dataset S11) to further validate the results. We also used PRIMUS (35) to help solve the pairwise relationships for certain complex scenarios.Our approach involved initially identifying first-degree, second-degree, and third-degree relatedness solely based on lcMLkin results (referenced as Dataset S6, Column R). Subsequently, we compared these findings with outputs from KIN, READ, and ancIBD. For pairs exhibiting ambiguous or unclear relatedness patterns that diverged from theoretical coefficients, we adjusted the relationships accordingly (referenced as Dataset S6, Column S). These adjustments were

made following comprehensive comparative analyses involving KIN, READ, ancIBD, and guided by the structural framework of intermediate pedigrees outlined in subsequent paragraphs.

S6.1 Pedigrees based on first-degree relatedness

The first step was to identify all the first-degree related pairs of individuals, which included parent-offspring relationships and full siblings. We identified 24 first-degree related pairs directly from lcMLkin results (Dataset S6, Column R), including 11 sibling pairs (theoretical pattern: k0 = 0.25, k1 = 0.5, k2 = 0.25) and 13 parent-offspring pairs (theoretical pattern: k0 = 0, k1 = 1, k2 = 0). All the 24 first-degree related pairs were validated in READ (Dataset S7, Column E). In KIN, all the 24 first-degree pairs were also validated (Dataset S8, Column M). We further looked into details about KIN's first guess and found all the parent-offspring and sibling pairs identified in lcMLkin were also identified as parent-offspring pairs and siblings in KIN except for the pairs between COL_053 - COL_049 (k2 = 0.12) and COL_057 - COL_049 (k2 = 0.19). In lcMLKin, they were identified as siblings since the k2 value of the second pair is 0.19, which is very different from 0 and closer to 0.25, and COL_053 - COL_057 are siblings (k0 = 0.22, k1 = 0.57, k2 = 0.21), so we would consider both of the pairs to be siblings. In KIN they were identified as parent-offspring pairs, though the within degree second guess indicated they can also be siblings. We adapted the results given by lcMLkin in this case. We also noted that the pair COL_106 and COL_110 were identified as first-degree related in READ. Considering their coefficients (k0 = 0.37, k1 = 0.48 and k2 = 0.15) in lcMLkin, we then considered them as siblings.

We constructed the first intermediate pedigrees based on the first-degree relateded pairs. This gave 8 pedigrees (Fig. S10a). For Pedigree I, there were three siblings, and all of them were parent-offspring related with COL_150. Considering COL_128 only parent-offspring related with COL_146, we concluded that COL_151 was the grandmother while COL_128 was the granddaughter. For Pedigree II it is not possible to determine orientation between the parent-offspring pair just based on observed first degree relatedness. We decided the orientations of pedigree III and IV based on the following logic. For instance, in Pedigree III COL_083 and COL_084 were siblings and were parent-offspring related with COL_087, while COL_087 was also parent-offspring related with COL_102. This pattern is only consistent with COL_102 being grandmother and COL_084 and COL_083 as the grandchildren.The osteology age and C14 information also suggested that COL_102 was much older than COL_083 and COL_084 (Dataset S1). Pedigree V, VI and VIII both consisted of two or three siblings. Pedigree VIII consisted of parents-offspring trio.

S6.2 Pedigrees integrating second-degree relatednes

We then identified 22 second degree related pairs based on lcMLkin (theoretical pattern: k0 = 0.5, k1 = 0.5, k2 = 0). 19/22 pairs were also identified in READ. The three pairs (COL_017 - COL_140, COL_142 - COL_140, COL_069 - COL_140) that were not identified in READ were identified in KIN in the first guess. Similarly, there were three pairs that were not identified in KIN (COL_097 - COL_142, COL_097 - COL_069 and COL_097 - COL_017), but were identified in READ. We noted that COL_140 (0.91x) was the common individual in the first three pairs and COL_097 (0.15x) was the common individual in the second three pairs. Unfortunately, their coverages are both below 1x so we could not test the relationships using ancIBD. However, at least one method plus lcMLkin identified the second degree relatedness between these pairs.

By including second-degree pairs, we were then able to combine and extend our pedigrees (Fig. S10b). We first solved the position of COL_097. In lcMLkin, though it did not show perfect parent-offspring relatedness with COL_143 (k0 = 0.21, k1 = 0.79, k2 =0), COL_083 (k0 = 0.28, k1 = 0.63, k2 = 0.08) and COL_084 (k0 = 0.28, k1 = 0.6, k2=0.12), they were identified as first-degree related in READ. Also, both lcMLkin and READ suggested COL_097 was second-degree related with COL_142 and COL_069, while COL_083 and COL_084 were second-degree related with COL_143 (validated by lcMLKIN, READ and KIN). Combining all these findings, we concluded COL_097 was the father of COL_083, COL_084 and COL_143, though they had different mothers. This connected Pedigree III and IV in Fig S10a.

We also connected Pedigree I and II as COL_093 was second-degree related with the three siblings in Pedigree I (COL_146, COL_145 and COL_150). The individual COL_017 was second-degree related with COL_143, COL_097 and COL_140, and it carried the same mtDNA haplotype with COL_140 and COL_143, while also carrying the same Y chromosome haplotype with COL_143. Therefore we concluded that COL_017 was the grandson of COL_097 and COL_140, and his mother was the sister of COL_143. We also noticed that COL_017 was second-degree related with COL_053 and COL_057, and COL_057 was second-degree related with COL_053. Thus we connected Pedigree IV and V through COL_017. For Pedigree VI, we identified an individual (COL_121) that was second-degree related to COL_106. We further found COL_121 was also connected with COL_110 with kinship coefficients that close to that expected for second-degree relatedness (k0 = 0.65, k1 = 0.32 and k2 =0.03), so we conclude that COL_121 was equally connected with the two siblings.

The results of integrating second-degree relatedness combined previous pedigrees, resulting in a total of five pedigrees at this stage.

S6.3 Final Pedigrees

In the third step we examined pairs displaying third-degree relatedness (theoretical pattern: k0 = 0.75, k1 = 0.25, k2 = 0). We did not consider any relationship deeper than third-degree in the construction of the pedigrees based on the known resolution of lcMLkin, KIN and ad READ. In lcMLkin, we initially identified 24 pairs of third-degree related individuals. Among these pairs, 12 were also identified as third-degree related in the first guess by KIN, while an additional 11 pairs were identified as such in the second guess. One pair, comprising COL_097 and COL_020, initially categorized as unrelated, shifted to being identified as identical in the second guess. Remarkably, all pairs involving COL_097 were either predicted to be unrelated or identical to the other individual by KIN, suggesting a limitation in KIN's efficacy with this particular individual, likely due to its low coverage (0.15x).

For the newly identified third-degree relationships, 7/24 of them could be deducted from previously constructed pedigrees (Fig. S10b). These included pairs of COL_142 - COL_017, COL_069 - COL_017, COL_084 - COL_017, COL_150 - COL_092, COL_128 - COL_093, COL_146 - COL_092. The remaining third-degree pairs we identified then allowed us to solve the positions of COL_020, COL_017 and COL_057. The first one was second-degree related with COL_017, and third-degree related with COL_053, COL_143, COL_097 and COL_140. This is only consistent with a pedigree where COL_020 was the niece of

COL_017. For COL_017, it was second-degree related with COL_053 and COL_057, while third-degree related with COL_049 and COL_047, thus it can only be the grandson of COL_053. COL_057 was also second-degree related with COL_053 and third-degree related with COL_049 and COL_047, and it was second-degree related with COL_017, so the only solution for this pattern was that COL_053 was the half-sibling with COL_017.

We found COL_039 was third-degree related with COL_020, and by checking whether it was connected with others individuals by screening the lcMLkin results again, we noticed it was also related with COL_017 (k = 0.81, k1 = 0.19, and k2 = 0), and in KIN the second guess of this pair was third-degree related, so we adjusted this pair to be third-degree related. We also found that the individual COL_099 was third-degree related to the three siblings of COL_047, COL_049 and COL_053.

The individual COL_097 and COL_093 had coefficients k0 = 0.82, k1 = 0.17 and k2 = 0.01, which were similar to the coefficients found in the third-degree relationship, potentially connecting Pedigree I and II. However KINs first guess was unrelated and second guess identical, and COL_097 was a low coverage individual so could not be directly examined using ancIBD. Therefore we examined the ancIBD estimate (Dataset S9) between COL_093 and COL_143 (the son of COL_097), and found out that they have a pi-hat value of 0.059 (Dataset S9, column P) that corresponds to a fourth-degree relatedness. We also checked the pi-hat values between following pairs: COL_093 - COL_069 (0.03536), COL_143 - COL_092 (0.0290) and COL_143 - COL_145 (0.03136), which all showed fifth-degree relatedness. This validated the third-degree relatedness between COL_097 and COL_093 identified by lcMLkin. We also solved the orientation of COL_093 and COL_092 pairs by checking how they related to COL_143. The former must be the father as COL_092 only shared half of the genomes that were shared between COL_093 and COL_143. Therefore we connected Pedigree I and II after integrating the third-degree relatedness and ancIBD results.

For Pedigree III, we noticed that COL_121 was third-degree related with the three siblings (COL_136, COL_148 and COL_126), and COL_126 was third-degree related with COL_106 and COL_110. This result connected Pedigree III and IV. The final pedigrees were shown in Fig. S10c

S6.4 Compare third-degree relatedness identified in lcMLkin and KIN

We noted that out of the 24 instances of third-degree relatedness identified in lcMLkin, only 12 were confirmed in the first guess by KIN. To delve deeper into this, we examined the pi-hat values of all available third-degree related pairs (totaling 32, accounting for adjusted third-degree relatedness) provided by ancIBD. However, due to the prerequisite of a minimum coverage threshold (1x) suggested for ancIBD analysis, only 12 pairs were eligible for inclusion in the analysis. Notably, all 12 pairs exhibited connections, with pi-hat values ranging between 0.06277 and 0.1893 and a mean value of 0.09967, which did not deviate a lot from the theoretical pi-hat values for the third-degree relatedness (pi-hat = 0.125). However, for the 12 pairs identified in ancIBD, KIN only identified 6 out of 12 pairs as third-degree related in the first guess. For the remaining 6 pairs, KIN identified them as third-degree related in the second guess. These findings suggest that KIN tends to be more conservative in predicting third-degree relatedness compared to lcMLkin.

S7. Bootstrapping results of fastNGSadmix

Yijie Tian

In order to assess the reliability of our fastNGSadmix admixture coefficients, we implemented a simple block bootstrapping procedure (36) to estimate confidence intervals. A block bootstrap approach was chosen in order to incorporate the potentially inflated error arising from the correlation structure of neighboring SNPs being in linkage disequilibrium. Bootstrapping was performed over 10Mb non-overlapping blocks (as such the genome is divided into 300 discrete blocks across all autosomes), where 300 blocks are sampled randomly with replacement. We ran 100 bootstrap iterations for each individual and plotted the 90% confidence intervals (ranging from the 5th to the 95th quantile estimates) and the 50% confidence intervals (ranging from the 25th to the 75th quantile estimates) for each ancestry component across all 52 individuals (Table S1, S2, Fig. S12 and S13).

For the modern ancestry panel, the mean of the standard deviation (SD) in ancestry coefficient was between 0.3116 % (FIN) to 9.766% (IBS). The non-European ancestries have minimal SDs across samples and ancestries (0.42%) while the European ancestries have relatively larger SDs (6.6%). We observed COL_110 had the highest SD for the three non-European ancestries (YRI, SAS and EAS) as well as TSI (Table S1). For the four European ancestries, the highest SD (25.26%) was observed in COL_095 for the IBS component. We suspected that the larger error profiles for the European populations was due to ancestry switching between pairs of populations with very low genetic differentiation (i.e. TSI vs IBS and CEUGBR vs FIN). Indeed, when combining these two pairs of components together we observed much narrower confidence intervals, supporting our hypothesis of close ancestry switching. For example, when integrating IBS with TSI, the standard deviation (4.402%) decreased compared to using IBS (9.766%) or TSI (9.322%) independently (Fig S12a,c and d).

For the penecontemporaneous panel, the range of mean SD was between 0.1010% (SUBSAHARAN) to 11.89% (NGBI). We observed a similar pattern as in the modern panel where non-Europeans ancestries had much smaller SDs. Similarly, COL_110 had the highest SD across three non-European ancestries (EASIA, SUBSAHARAN and NAFRICA) (Table S2). The highest SD across the penecontemporaneous panel was 27.20% in the NGBI panel (individual COL_097). Again, this was clearly driven by ancestry switching between closely related European source populations (NGBI v SCAND). When combining NGBI_SCAND ancestries we again observed much smaller SD (5.793%) compared with using NGBI (11.89%) and SCAND (8.106%) ancestries independently (Fig. S13a,c and d).

We note some minor inconsistencies in estimates of genetic ancestry within pedigrees, in particular lower estimated IBS ancestry in the offspring of Pedigree III compared to their parents, as well as an observation of minor contemporary North African ancestry in COL_106 but sub-Saharan ancestry in their sibling COL_110. It is important to appreciate that all clustering methods such as fastNGSadmix have associated errors in ancestry estimation that will be a function of variables such as data quality (in particular coverage, of which all our samples would be considered low compared to modern DNA) and how closely

related the tested source populations, as well as multimodality during maximum likelihood convergence (which we attempt to mitigate via multiple runs).

For the case of COL_110, which in particular is one of our lowest coverage samples (0.03), we expect somewhat high uncertainty in its ancestry estimate. Indeed it has the highest SDs for four of the seven ancestry components across all samples for the modern panel and three for the penecontemporaneous panels, including both African ancestries.). Its 50% CI overlaps with COL_106 for North African ancestry, and its sub Saharan African ancestry CI includes a lower bound of 0%. Yet the analysis still captures the salient features of COL_106 of predominant southern ancestry and 5-10% African ancestry.

With regard to Pedigree III, all three samples are less than 1x coverage and the primary issue is ancestry estimated to be predominantly IBS in the two parents (COL_002 and COL_050) is estimated to be TSI ancestry in the child, COL_008. However the estimated 95% CIs for all three samples overlap for both TSI and IBS ancestries separately and the 50% CIs overlap for the combined TSI+IBS ancestry. COL_008 is noteworthy for having some of the largest CIs in the whole dataset across all ancestries. When examining their combined TSI+IBS ancestries, there is very little discrepancy in ancestry estimates across the pedigree.

S8. Bardonecchia and Torino-Lavazza

Caterina Giostra, Yijie Tian, István Koncz

Bardonecchia. In 2005, multiple graves were found near the high medieval castle of Tur d'Amun, Bardonecchia. The site is located in the Alps, west of Turin, Piedmont, in close proximity to the French-Italian border and together with another site excavated at Cesana Torinese. Both sites were located in strategically important positions to control and oversee the connections between the Gaul and Piedmont, Northwest Italy. After the establishment of the Frankish Kingdom by the Merovingians, this area soon became part of its (Franco-Burgundian) territories (37).

Altogether 16 graves were identified, 4 of which were empty; the pits were simple or stone-lined. The west-east oriented graves were arranged in north-south rows. The total number of individuals is 23 (some graves were reused multiple times) with a higher ratio of males. Similarly to Collegno, the dimorphism between men and women was low, with tall, robust women. Few individuals showed signs of trauma interpreted as injuries caused by weapons. The site is dated to the 6th and 7th centuries archaeologically based on the few graves that contained artefacts, including weapons (scramasax) or weapon belts. The site was interpreted as a cemetery of a 'Frankish-Burgundian' group that was tasked with controlling the valley (37). Previous mtDNA analysis had recognized a different mitochondrial composition from that of other Lombard-era cemeteries in Piedmont (38, 39).

Torino-Lavazza. An early Christian cemetery in Turin was unearthed that developed from a group of mausoleums. The site is found in the present-day industrial block of Lavazza located across the Dora River, near a late Roman necropolis north of the ancient city, A church was built on top of this cemetery: it can be dated to the second half of the 4th and early 5th centuries and can perhaps be identified with the basilica of Saint Secundus. Burials

were found both inside and outside of the church. Unfortunately, most graves were opened and the deceased were removed when the church was closed in the late Middle Ages. The types of graves are masonry chest or brick-covered; almost no grave goods were found. Two radiocarbon dates provide the following time ranges: 400-550 AD (95.4%) and 380-580 AD (95.4%). The site was interpreted as one of the cemeteries of the early Christian community in the late antique city of Turin (40, 41).

Genetic diversity and pedigrees. PCA was conducted on individuals from Bardonecchia (n=8) and Torino-Lavazza (n=7), using the POPRES dataset for comparison (Section S3, Fig 6). The results demonstrated that individuals from Bardonecchia formed a close-knit cluster with western and southern European populations. Conversely, individuals from Torino-Lavazza exhibited a broader distribution and were grouped alongside central and southern European populations.

Furthermore, a model-based clustering analysis using fastNGSadmix was performed on all individuals, utilizing the model panel derived from the 1000 Genome project (Fig. S14). The analysis revealed that the prevailing genetic component observed across all individuals was TSI (60.7%). This component was consistently present in each individual. Additionally, a significant proportion of individuals displayed IBS (19.9%) and CEU+GBR (15.9%) genetic components.

Collectively, the results of both the PCA and model-based analysis underscore the distinct genetic ancestries of individuals from Bardonecchia and Torino-Lavazza, particularly when compared to Collegno. Notably, the individuals from Pedigree I in Collegno exhibited marked differences, with most of them clustering with northern European populations and showing a substantial presence of CEU+GBR ancestry.

We also conducted biological relatedness analysis for all individuals from the two sites using lcMLkin. It detected a pair of parent-offspring (Bard-T10 and Bard-T11) and a pair of third degree related individuals (Bard-T11 and Bard-T2).

S9. Strontium isotope (87Sr/86Sr) analysis

István Koncz

In the Amorim et al. 2018 paper Sr results of 27 human samples were published together with 13 environmental samples from the wider geographical area. We complemented this sampling with an additional 26 human samples to have Sr values for all genetically analyzed individuals if preservation of the osteological material made it possible. For sampling we preferred late forming teeth (second and third molars), if they were available (Dataset S1)

The earlier study established local Sr range between 0.708 and 0.71 based on the environmental samples collected around the site and in the wider region and identified a series of individuals with non-local Sr values. With the additional samples we implemented additional methods to define local Sr range:

1) a baseline was calculated with two standard deviation of the human results (mean: 0.70934; standard deviation: 0.00064; double standard deviation: 0.00128) that gave us a Sr range between: 0.70806 - 0.71062 that is very similar to the earlier local Sr range.

2) We considered the distribution of the human dataset from the cemetery of Collegno, the deciduous and permanent teeth of nonadults (below the age of 15 years) in particular. We concentrated on nonadults' teeth because they show Sr values that were stored a few months or years prior to the death. The short time span between the incorporation of Sr and the death of the individuals, increases the probability of Sr being of local origin. All six subadult individuals showed very similar Sr values without any slopes or breaks with lowest value of 0.70926 and highest value of 0.70957. Several earlier series of Sr isotope data confirm significantly less variability of 87Sr/86Sr ratios among the teeth of children than among those of adult individuals suggesting that very similar values among subadult individuals might indicate the isotope composition of the locally Sr originating from the agriculturally exploited land within a limited radius around the site. A second baseline was calculated with two standard deviation of the nonadult results (mean: 0.7094; standard deviation: 0.000123; double standard deviation: 0.000246) that gave us a Sr range between: 0.709154 - 7.09646 that is narrower than the earlier local Sr range.

Based on the different approaches we identified a more conservative, wider and a more strict, narrower local Sr range ('local center'), the former based on environmental samples and double standard deviation of all human samples, the latter based on the nonadult values. The 'local center' range falls in the middle of the more conservative range and divides it to two zones: local Sr values above 'local center' range (named 'local high') and values below 'local center' range ('local low'). These ranges also correlate well to the visible slopes and brakes among the human values.

All nonadults (6 individuals), among adult females 8 out of 15 (53.3%) and among adult males 17 out of 29 (58.6%) fall inside the 'local center' range, while 12 out of 15 (80%) and 28 out of 29 (96.6%) fall within the more wider local range with multiple individuals sitting right at the borders of the respective ranges. Individuals showing Sr value within the 'local center' range suggest that they grew up locally or in a region with similar Sr value. Individuals falling outside of the wider local range (above of 'local high' or below of 'local low') spent the time when their sampled teeth were forming elsewhere, in a region with different locally available Sr value than the Collegno region. (Fig S16)

Based on the estimated local Sr ranges we were able to identify a set of individuals with non-local Sr values. Means of male and female values were very similar (0.70931 and 0.70936), with female values showing higher variance (7.6677E-07 and 2.9895E-07). We used Mann-Whitney (U: 241; z: 0.12639; p (same med.): 0.89942; Monte Carlo permutation: p (same med.): 0.9012) and Kolmogorov-Smirnov (D: 0.16599; p (same dist.): 0.89508; Monte Carlo permutation: p (same dist.): 0.828) tests to compare the datasets of males and females, differences were not significant (Mann-Whitney U : 241 z :0.12639; p (same med.): 0.89942; Monte Carlo permutation: p (same med.): 0.9012). (Fig S17) Four females (COL_008, COL_020 and COL_035, COL_147) fall outside of both local ranges. Three males (COL_034, COL_023, COL_094) are very close to the local environmental values and lie outside of the 'local center' range. Additional individuals (females: COL_102, COL_056, COL_087, COL_048 and males COL_093, COL_009, COL_049, COL_050) fall within the wider local range, but outside 'local center'. They might be individuals who are non-local to the Collegno settlement area, but grew up in the wider geographical region. Four additional males (COL_097, COL_086, COL_063, COL_112) sit on the line between 'local center' and 'local high' with slightly higher values than those of the subadults, their values might be part of the local range or again indicate individuals from the wider geographical region.

S10. Carbon (δ13C) and Nitrogen (δ15N) isotope analysis

Sarah Defant

S10.1 Background

Stable isotope analysis provides evidence on the lifestyle of buried individuals, independent of practices surrounding their death and burial (42).

The principles of palaeodietary reconstruction based on carbon ($\delta^{13}C$) and nitrogen ($\delta^{15}N$) stable isotope composition of bone collagen are well-established and are dealt with extensively in various publications (42–48). Therefore, only a brief summary will be given here.

Carbon isotope values differ between marine and terrestrial environments due to differences in the sources of inorganic carbon used during photosynthesis. In marine environments, inorganic carbon values are approximately 7‰ more positive than atmospheric carbon values (42). Further, variation in the terrestrial ecosystems is an effect of differences in photosynthetic pathways.

In C_3 plants, which are common in temperate environments, strong discrimination against the heavier isotope (^{13}C) during photosynthesis leads to lower $\delta^{13}C$ values (-26‰ on average) in almost all trees and temperate grasses, including wheat and most other Old World cereals (49–51). C_4 plants, such as sorghum, millets and maize, which are more common in tropical environments, have less negative $\delta^{13}C$ values (-13‰ on average). Since the $\delta^{13}C$ value ranges of these two plant groups do not overlap, we can estimate dietary contributions from C_3 and C_4 plants.

In animals, fractionation occurs between the consumed carbon (e.g., from a plant) and the fixed compound and tissue (e.g., bone collagen) of the consumer. The extent of fractionation varies significantly based on the consumer and the type of body tissue (muscle vs. bone collagen). The average bone collagen $\delta^{13}C$ values of herbivores are typically around 5‰ higher than the average $\delta^{13}C$ values of plant tissues, while the bone collagen $\delta^{13}C$ values of omnivores or carnivores feeding on those herbivores are only enriched by 1-2‰ (50, 52, 53).

Therefore, the $\delta^{13}C$ values reflect isotopic signals from consumed plants and animals, and can be used to infer whether dietary sources derived from a terrestrial or marine environment, and whether the consumed plants follow the dominant C_3 or less common C_4 pathway of photosynthesis (42, 46, 50, 54, 55).

Bioavailable nitrogen stable isotope values ($\delta^{15}N$) are similar between marine and terrestrial environments. The variation observed in terrestrial ecosystems results from the activity of nitrogen-fixing microbes, which is affected by temperature and moisture availability. Plants typically absorb all bioavailable nitrogen, and their δ15N values usually reflect the input from the nitrogen pool present in the soil. Furthermore, the nitrogen isotope values of plants can be influenced by artificial irrigation or manuring (56).

In animals, fractionation occurs between the consumed proteins (from a plant or animal) and the fixed compound and tissue (e.g., bone collagen) of the consumer. The trophic enrichment is associated with the amount of protein in the bulk food. In a low-protein diet (e.g. that of herbivores) the spacing between diet and collagen is lower than in a protein-rich diet (e.g. that of carnivores). The reported variation in $\delta^{15}N$ enrichment per trophic level varies between 2 and 6‰ (50, 57). Since nitrogen can be derived only from proteins, $\delta^{15}N$ values are widely adopted to study the trophic position of an organism.

In aquatic environments, the trophic relations are more complex, resulting in higher $\delta^{15}N$ values for top marine predators (58–60). Therefore, it can also be used to distinguish between terrestrial and marine diets.

One specific application of $\delta^{15}N$ analysis is the detection of breastfeeding patterns, as breastfed infants are one trophic level above their mothers (42, 50, 61–63).

The implementation of strontium isotope ratios ($^{87}Sr/^{86}Sr$), most commonly used in archaeological contexts for reconstructing past mobility and described in *S5 and Dataset S1*, can also be exploited to further differentiate between terrestrial and marine resource consumption. For this, the specific $^{87}Sr/^{86}Sr$ ratio of global sea water (0.70917), reflected also in marine resources and their consumers (64) can be utilized and compared to the human strontium isotopic values. If the human $^{87}Sr/^{86}Sr$ values display a clear shift towards this value, it can be used as an additional indicator for marine resource consumption.

S10.2 Material

Amorim et al. (29) published results of carbon and nitrogen stable isotope analyses of 31 humans from Collegno together with six faunal samples. Due to lack of zooarchaeological remains from Collegno dated to this time period, these faunal samples derived from an excavation in Piazza Castello Turin (1st - 3rd/4th century AD). Because these animal remains derive from the Roman period, with an economic context that differed from the Lombard one investigated in the present study, we decided not to include them here. In order to accurately create a representative faunal baseline, samples directly from the site, or from penecontemporaneous neighboring sites would be needed. However such samples are not currently available.

In order to still allow for a general placement of the human values from Collegno within their wider environmental context, we opted to use animal isotopic values from several contexts with comparable chronology, which are situated further to the West in the Province of Bergamo (65).

We undertook isotopic analyses on 28 additional human samples to complement the aDNA analysis reported in the present paper. Bones with a higher proportion of trabecular bone and therefore a faster turnover rate, such as ribs, were chosen as the preferred bone sample to produce insight into the diet approximately five years prior to the death of an individual (50, 66). If these were not available, fragments of long bones, and in one instance, a metacarpal were selected.

S10.3 Methods

S10.3.1 Stable Isotope Analysis

Collagen was extracted from human bones following the standard laboratory protocol of the Department of Bioarchaeology at the University of Warsaw, based on the method detailed by Longin (67) and modified by Brown et al. (68). The surfaces of the bone pieces, weighing between 400 mg and 600 mg, were manually abraded and subsequently demineralized in 0.3 M aq. HCl at room temperature until the mineral components were dissolved. Subsequently, the samples were rinsed with deionised water, gelatinized in hydrochloric acid solution (pH 3) at 70°C for 48 h, filtered using Ezee Filter separators, and then frozen. Finally, they were subjected to freeze-drying.

Isotopic values were measured in the Environmental Isotope Lab, Geosciences at the University of Arizona, USA, using continuous-flow gas-ratio mass spectrometer (Finnigan Delta Plus XP) coupled with an elemental analyzer (Costech). Measurement of elemental

concentration was standardized based on acetanilide. Samples were measured against internal (acetanilide) and international (USGS40 and USGS41a) standard materials. Measurement error, expressed as 1 sigma, was estimated based on the repeated measurements of internal standard materials (better than ± 0.1‰ for $\delta^{13}C$ and ± 0.2‰ for $\delta^{15}N$).

For further analysis, samples with carbon concentration greater than 13%, nitrogen concentration greater than 4.8% (43) and atomic C/N ratio between 2.9-3.6 were accepted (43, 69, 70) .

S10.3.2 Statistical Analysis

Penecontemporaneous archaeological records from various regions, including Italy (71–73), suggest that adolescents were more likely to be treated as adults rather than children. We therefore pooled adults and adolescents together and excluded all other non-adult individuals for analyses concerning the entire community. For analyses pertaining to the Pedigrees, only those under the age of three (COL_083) and individuals classified as *Infans 1/Young Child* without specific ages (COL_126, COL_136) were excluded (highlighted in Fig. S18b) due to the potential influence of breastfeeding on $\delta^{15}N$ values.

Statistical analyses were conducted to identify significant differences in stable isotope values between subsets of different biological sex.

Additionally, we wanted to explore temporal trends, differences between members of identified kinship groups and outsiders and further investigate the main Pedigree I. A final set of statistical analyses aimed to investigate significant differences between main identified ancestry components.

As the normal distribution cannot be expected in the dataset, only non-parametric statistical tests were performed, including the Mann-Whitney U-test for two groups and the Kruskal-Wallis test for three or more groups with subsequent post-hoc Dunn test.

Spearman's correlation values were calculated for $\delta^{13}C$ and $\delta^{15}N$ values in the entire adult population, and also for the absolute difference of human to marine $^{87}Sr/^{86}Sr$ values against $\delta^{13}C$ and $\delta^{15}N$ respectively, to investigate the contribution of marine resources.

Hierarchical Cluster Analysis was performed to investigate the distribution of stable isotope values in the two main clusters of Pedigree I (CL1 and CL2). For this, Euclidean distances between individual cases were calculated, which provided the base for the subsequent creation of hierarchical clusters employing Ward's method.

Additionally, a (Euclidean) distance matrix for all individuals with available aDNA data was created using $\delta^{13}C$ and $\delta^{15}N$ values. It was used to test for significant differences between pairwise distances in the subset of closely related (first- and second-degree) individuals and the subset of non-related individuals, employing the Mann-Whitney-U test.

Statistical testing was applied only when the size of all compared subsets was higher than 5 individuals.

All statistical analyses were performed using R Statistical Software (v4.3.0) with the *stats (74)* and the *dunn.test* (v1.3.5) (75) packages.

S10.4 Results

Out of 28 samples, 27 met the quality criteria. For further analysis, the newly obtained results were combined with those already published in 2018. Among these 58 samples,

aDNA data are available for 48, while both strontium and aDNA data are available for 41 samples.

Results of the stable isotope analysis of all human samples can be found in *Dataset S1*.

S10.4.1 Animal Baseline

For a general placement of the human values within their environmental and chronological context, published faunal data from six sites (Antegnate, Caravaggio, Casirate D'Adda/Treviglio and 3 sites within the area of Romano di Lombardia) in the province of Bergamo were used (Fig. S19).

The average values and ranges of these animals grouped by their taxa can be found in Tables S4 and S5. For a detailed description of the sites and their respective samples see (65).
It must be taken into consideration that these animal data derive from an area approximately 200 km east of Collegno. They may therefore be influenced by a variety of factors, such as different environmental conditions. However, they are all dated to the same period and therefore suitable for providing an idea of available resources in the larger region and the time period under study.

S10.4.2 General Observations

Individuals from Collegno exhibited $\delta^{13}C$ values ranging between -19.6‰ and -17.0‰ (mean -18.4 ± 0.6) and $\delta^{15}N$ values between 7.4‰ and 10.3‰ (mean 8.7 ± 0.7).

There was a significant, but weak, correlation between $\delta^{13}C$ and $\delta^{15}N$ values in adult individuals (r_s = 0.31, p = 0.03). Among other factors, this could be linked to the consumption of marine resources. We therefore opted to incorporate the $\delta^{13}C$ and $\delta^{15}N$ data along with the $^{87}Sr/^{86}Sr$ data detailed in *Dataset S1* to further explore the identified correlation.

S10.4.3 Implementing $^{87}Sr/^{86}Sr$ into the analysis of $\delta^{13}C$ / $\delta^{15}N$ isotopes

It was not possible to investigate the human values only for a shift towards the value of seawater (0.70917) (64) reflected also in marine resources and their consumers, as this value lies within the identified bioavailable local strontium range for individuals at Collegno (*S4*). However, we calculated absolute differences between adult human values and the value of seawater in the Mediterranean Sea, which thus allowed us to calculate the Spearman correlation coefficient between these absolute differences and $\delta^{13}C$ and $\delta^{15}N$ respectively. There was a small negative and non-significant correlation between these absolute differences and $\delta^{13}C$ values (r_s = -0.20, p = 0.18), and a small positive, non-significant correlation between the calculated differences and $\delta^{15}N$ values (r_s = 0.13, p = 0.40).

When removing the outlier individuals in respect to strontium isotope values (n=15) we found no correlation between the absolute differences in $^{87}Sr/^{86}Sr$ and $\delta^{13}C$ values (r_s = 0.04, p = 0.85) and a small positive, non-significant correlation between these differences and $\delta^{15}N$ values (r_s = 0.19, p = 0.31).

S10.4.4 Chronological Aspects

With the combination of archaeological dating and genetic relationships between individuals supported by radiocarbon dating we were able to identify general trends (organized into four theoretical phases for analytical purposes) in the development of the cemetery.

The Kruskal-Wallis test revealed a significant difference ($H(2) = 1.481$, $p = 0.003$) in $\delta^{13}C$ values among developmental phases (Foundation, Development I+II and Development III). Subsequent focused comparison of the mean ranks between groups showed a significant difference between the earlier (Foundation) and later members (Development I+II) of Pedigree I, both combined with their respective associated unrelated individuals ($p = 0.03$), as well as between the later members of the large Pedigree I combined with their associated individuals (Development I+II) and the late newcomers (Development III) ($p = 0.001$). No significant difference was found between the earliest and the latest members of the community.

No significant difference was detected between $\delta^{15}N$ values and developmental phases ($H(2) = 3.6942$, $p = 0.16$).

S10.4.5 Demographic Characteristics

Although female individuals had slightly lower average $\delta^{13}C$ values than male individuals (Fig. S18a and Table S6) and slightly higher $\delta^{15}N$ values (Fig. S18a and Table S7), the differences were not significant (Mann-Whitney U-test; $\delta^{13}C$: $p = 0.33$; $\delta^{15}N$: $p = 0.91$).

Statistical analysis could not be conducted to assess differences between adults and non-adults because there were only three post-weaning non-adults left in the dataset, after adults and adolescents were pooled together.

S10.4.6 Variation between Pedigrees

Summary statistics for $\delta^{13}C$ and $\delta^{15}N$ values of all individuals from the three identified Pedigrees (and their subdivisions), as well as the remaining population, can be found in Tables S8-9.

Due to the small sample sizes of Pedigrees II and III, no statistical analyses comparing the respective Pedigrees with each other could be carried out. However, the following observations were made based on descriptive statistics:

The $\delta^{15}N$ values appeared relatively homogeneous amongst the entire population.

Pedigree I-CL1 and I-CL2 and Pedigree II had similar values in both $\delta^{13}C$ and $\delta^{15}N$, while Pedigree III exhibited divergent, more depleted $\delta^{13}C$ values.

The unrelated individuals showed the largest range in $\delta^{13}C$ values, compared to members of Pedigrees and their range of $\delta^{15}N$ values was also amongst the largest.

Although there are minor differences among Pedigrees and their clusters, there is a significant degree of overlap in values, even among the most distinct group averages. To provide more definitive conclusions, additional extensive sampling would be required.

S10.4.7 Variations within Pedigree I

Further in-depth analyses could be undertaken focusing on Pedigree I (Fig. S20).

Members of Pedigree I exhibited less negative mean $\delta^{13}C$ values (mean = -18.1‰) than the rest of the community (mean = -18.6‰), and the difference was statistically significant (Mann-Whitney U test, $p = 0.031$*)*.

The $\delta^{15}N$ values, however, are similar for members of Pedigree I (mean = 8.76‰) and all other individuals (mean = 8.79*‰*), and the difference was not statistically significant (Mann-Whitney U test, $p = 0.90$*)*.

Within Pedigree I, CL-2 had the highest mean δ^{13}C values (-17.96‰), followed by CL2/3 (-18.03‰) and CL1 (-18.18‰). CL-3 has the lowest mean δ^{13}C values (-18.5‰), noting the presence of one outlier (COL_099) with a much higher δ^{13}C value.

Individuals from group CL2/3 have the highest mean δ^{15}N (9.67‰), compared to individuals from all other clusters of Pedigree I (between 8.5‰ and 8.7‰). Two of them also have the highest average δ^{15}N values of the entire population at Collegno.

During the initial analysis of Pedigree I we observed two clusters amongst the individuals of CL-1 and CL-2, one of which appeared more enriched in both δ^{13}C and δ^{15}N.

Subsequent Hierarchical Cluster Analysis (Fig. S21) supported these initial observations, separating the individuals from CL1 and CL2 together into two clusters.

While these two main clusters do not appear to be connected to sex, age or kinship group of Pedigree 1, they do coincide with developmental phases of the site.

The elevated δ^{13}C and δ^{15}N values are consistently found among individuals from the third and fourth generations, belonging to a later phase of the site (Development I and II in Fig. S22 a-b), as compared to members of the early arriving group.

Additionally, the hierarchical cluster analysis suggested similarities in diet among closely related individuals (e.g. COL_084 and COL_087, COL_092 and COL_093 and COL_069 and COL_140).

When comparing I-CL3 to I-CL1 and I-CL2 we were also able to observe that the three siblings of I-CL3 cluster together, albeit only in regard to their δ^{13}C values. We therefore also examined the remaining adult sibling groups (Siblings CL1: COL_145, COL_146 and COL_150; Siblings CL2_2: COL_069 and COL_142; Siblings CL3: COL_047, COL_049, COL_053) and found similar patterns for all of them (Fig. S23).

Each group of siblings showed comparable δ^{13}C values within their own set, but when comparing these sets to one another, their δ^{13}C values appeared to differ.

To further investigate this aspect, we calculated Euclidean distances between all members of the community and subsequently found a significant difference in mean distances between closely (first- and second-degree relatives) related individuals and more distantly or unrelated individuals (Mann-Whitney U test, $p = 0.0021$), giving further support to these initial observations.

S10.4.8 Ancestry components as differentiating factors

The final set of analyses aimed at investigating differences between individuals based on their admixture results.

To start with, all individuals with more than 50% Central European/Great British (CEU+GBR) ancestry in the modern panel ("North") and all individuals with more than 50% Tuscan (TSI) or more than 50% Tuscan (TSI) plus Iberian (IBS) ancestry ("South")pooled together respectively.

Individuals with predominantly Northern ancestry had higher average δ^{13}C values (mean = -18.1‰), than individuals with predominantly Southern ancestry (mean = -18.5‰). The difference was statistically significant (Mann-Whitney U test, $p = 0.03$).

Individuals with predominantly Northern ancestry had slightly higher δ^{15}N values (mean = 8.83‰) compared to individuals with predominantly Southern Ancestry (mean = 8.79‰). This difference, however, was not statistically significant (Mann-Whitney U test, $p = 0.86$).

Fig. S24 reveals a potential underlying cause for the statistically significant difference in $\delta^{13}C$ values. Individuals with a larger amount of Iberian ancestry, grouped initially into the Southern ancestry group, include several individuals with particularly low $\delta^{13}C$ values.

Comparisons between the genetic results and the archaeological record suggest that the majority of individuals with a larger proportion of Iberian ancestry (IBS) date to the last phase of the site. This finding likely accounts for the significant difference observed in the data. Consequently, we investigated how these individuals compared to the rest of the population, with particular focus on their comparison to individuals belonging to Pedigree I.

We found that these late arriving individuals with a larger proportion of the IBS component had indeed significantly lower mean $\delta^{13}C$ (mean = -19.02‰) values than the already established community (mean = -18.21‰) (Mann-Whitney U-test, $p = 0.003$).

Following the overall trend, there was again no significant difference in $\delta^{15}N$ values (mean = 8.76‰ for late arriving individuals with IBS component vs mean = 8.81‰ for the rest of the community)(Mann-Whitney U test, $p = 0.90$).

S10.5 Discussion

S10.5.1 Animal Baseline

$\delta^{13}C$ values of penecontemporaneous faunal data (Fig. S19) from the province of Bergamo (65) suggest that animals did not only have access to C_3 plants, but also to variable proportions of C_4 plants, most likely millets, through e.g. foraging or fodder. This is further substantiated by the presence of both broomcorn (*Panicum miliaceum*) and foxtail millet (*Setaria italica*) remains in the archaeological record, not only in neighboring sites in Bergamo but also in numerous penecontemporaneous sites throughout the broader region (76).

S10.5.2 General Observations

45 individuals have $\delta^{13}C$ values typical of a diet based on terrestrial C_3 plant consumption, however, 13 individuals (22%) exhibit higher $\delta^{13}C$ values (between -17‰ and -18‰), which suggests a higher proportion of C_4 plant consumption (77), either directly or indirectly through C_4-fed animals. Alternatively, this could also be associated with the inclusion of (imported) C_3 plants deriving from areas with different (lower) levels of precipitation or the incorporation of marine resources as hinted at by the correlation between $\delta^{13}C$ and $\delta^{15}N$ values.

As suggested in Amorim et al., 2018, the most likely consumed C_4 plant at Collegno would have been millet. Castiglioni et al.(78) were able to identify small amounts of charred broomcorn millet (*Panicum miliaceum*) at Collegno, and there are larger amounts of broomcorn and foxtail millet (*Setaria italica*) attested at other sites in Piedmont and adjacent regions (76). Similar patterns of increasing C_4 plant consumption in the 6th and 7th centuries have been observed in Tuscany (79), for example.

The contemporary animal data (65) includes a notable proportion of animals with less negative $\delta^{13}C$ values, which strengthens the argument that C_4 plants, likely millets, were available as a food source or fodder.

The identified positive correlation between $\delta^{13}C$ and $\delta^{15}N$ values observed in humans from Collegno, while there remains an absence of any significant shift in strontium values, suggests indirect consumption of C_4 plants through their consumption of these C_4-fed animals.

Additionally, we observed a subset of the population displaying elevated $\delta^{13}C$ and $\delta^{15}N$ values. The positive correlation between $\delta^{15}N$ and $\delta^{13}C$ values could be linked to a higher proportion of protein from sources enriched in $\delta^{13}C$, such as meat or dairy products from animals feeding on C_4 plants. Alternatively, it could also be associated with the consumption of marine protein. However, the absence of significant relationships between $^{87}Sr/^{86}Sr$ and $\delta^{13}C$ and $\delta^{15}N$ respectively, suggests that the increase in $\delta^{13}C$ and $\delta^{15}N$ values can more likely be attributed to the consumption of animals with a higher share of C_4 plants as fodder, than to the consumption of marine resources.

And lastly, there are several individuals exhibiting $\delta^{15}N$ values more than one trophic level above the average herbivore values in the wider region (4-5‰; (65)). This could suggest that at least a part of the population incorporated more omnivore meat (e.g. pork or poultry) into their diet, while others relied more on cattle derived protein. Zooarchaeological studies of other Northern Italian sites have shown that pigs tend to dominate over herbivores (sheep, goat, cattle) in lowland sites (80, 81), and isotopic studies have suggested an increase in pork consumption throughout the Early Middle Ages (79).

Many of these changes coincide with the different identified developmental phases of the site, as shown in Fig. S22 a-d and discussed in more detail below.

S10.5.3 Demographic Characteristics

No significant difference was found between females and males, which suggests that there was no sex-based discrimination/differentiation in diets. Female and male individuals had similar dietary patterns and therefore probably shared common food sources. The insufficient sample size of post-weaning non-adult individuals did not allow for any further investigation in the differences between adult and non-adult individuals.
However, we did observe that COL_083, who was estimated to be less than three years old at time of death, exhibited one of the highest $\delta^{15}N$ values (9.3‰). Although her $\delta^{15}N$ values did not appear elevated in comparison to the entire population, they showed a clear enrichment compared to her close relatives. The enrichment between mother (COL__087) and daughter (COL_083) suggests that COL_083 might have still been breastfed at or shortly before her death, while her brother (COL_084) had already been weaned. This might also be the case for COL_126. However, we have neither a precise age-at-death, nor a close relative available to investigate this further.

S10.5.4 Variations within Pedigree I

The significant difference identified in $\delta^{13}C$ values between members of Pedigree I and individuals outside of this kinship group, may be explained by dietary preference for C_4 plants, such as millet, over C_3 plants, different (drier) places of origin of consumed plants, or the potential inclusion of marine resources.

The absence of significant difference in $\delta^{15}N$ values suggests that access to protein was not necessarily tied to membership in a specific kingroup.

When investigating the various clusters of Pedigree I closer, it became evident that the individuals from I-CL1 and I-CL2 differ from I-CL3. Individuals from all three clusters formed their initial burial cores around the same time but were only connected later when one individual of I-CL2 married a member of I-CL3.

Based on their genetic make-up and their position within the burial ground (in a separate cluster, away from I-CL1 and I-CL2), I-CL3 appears different from the rest of Pedigree I.

Fig. S20 also showed that the three siblings from I-CL3 cluster together in terms of $\delta^{13}C$ values, which appear different compared to individuals from I-CL1, I-CL2 and their

third-degree relative COL_099. COL_099 might have followed a different diet, closer to I-CL1 and I-CL2, with a higher proportion of C_4 plants, while the rest of I-CL3 appear to actively go against this trend and preferred to adhere to a diet richer in C_3 plants. Notably, COL_099 was also buried in a location away from the rest of I-CL3.

The siblings of I-CL3 have equally elaborate grave goods as the individuals from I-CL1 and I-CL2. It therefore seems unlikely that the difference in diet is caused by a difference in social status. The data presented here is more consistent with a conscious choice made by members of I-CL3 and I-CL1+I-CL2 to reaffirm their own respective cultural or social identities through their dietary choices.

Individuals from Pedigree II, who were found buried in the same area and the same style as I-CL1 and I-CL2, also appear to follow the general dietary trend of Pedigree I, suggesting some sort of social cohesion between these groups too.

The Hierarchical Cluster Analysis grouped the individuals from Pedigree I (CL1 & CL2) into two clusters (Fig. S21), coinciding with different developmental stages of the site (Fig. S22 a-d).

The earlier individuals appear to have relied on a higher proportion of C_3 plants and C_3-foddered terrestrial animals, whilst the second cluster appears to have incorporated a larger amount of C_4 crops, which would explain the elevated $\delta^{13}C$ values, paired with a larger amount of protein, which would have resulted in elevated $\delta^{15}N$ values. Alternatively, they could have incorporated more C_4-fed animals. A contribution of marine resources was found to be unlikely.

It is also during these later phases of the site when the clusters of Pedigree I are connected through intermarriage and we see elevated $\delta^{15}N$ values - the highest $\delta^{15}N$ values in the dataset - amongst individuals of I-CL2/3. This could be associated with a higher intake of omnivore derived protein or potentially with the inclusion of freshwater fish. Their $\delta^{13}C$, however, appear to follow the trend of CL-1 and CL-2.

It is plausible that during the period when we observe these elevated values, the site had already established itself within its broader surroundings. As a result, individuals might have had access to resources not readily available in the immediate vicinity but could have been obtained through trade, such as grains from different regions (or different environmental contexts), marine fish or other distant food sources.

The observed dietary differences between earlier and later phases of Pedigree I could be attributed to shifting dietary preferences or changes in available resources. These variations may have been influenced by factors such as climatic conditions or alterations in agricultural and animal husbandry strategies during the different periods.

S10.5.5 Dietary Household Patterns?

When investigating the results of the Hierarchical Clustering Analyses further and comparing them with the genetic relatedness amongst individuals, we identified a potential connection between dietary patterns and family ties. The significantly shorter distances between all closely related individuals compared to more distant or unrelated individuals, further supported these initial observations that individuals who were closely related to each other appear to share a similar diet, both in terms of $\delta^{13}C$ and $\delta^{15}N$ values. This was, for example, the case for COL_087 and COL_084 or COL_093 and COL_092, both parent-offspring pairs, or COL_140 and COL_069, a grandparent-grandchild pair in Pedigree I. Furthermore, this was also observed beyond Pedigree I, in the later-arriving parent-offspring trio of Pedigree III - COL_ 002, COL_008, and COL_050.

We also noticed that adult siblings shared similar $\delta^{13}C$ values, but differed in their $\delta^{15}N$ values.

We interpret this as a potential indicator for siblings and close-knit (family) groups sharing similar dietary inclinations. An individual's dietary choice might therefore be shaped by their family's preference or access to dietary sources, especially in terms of the frequencies of consuming specific grains, such as millet.

S10.5.6 Ancestry Components as differentiating Factors

Our final set of analyses aimed to investigate whether and what role genetic ancestry could have had on an individual's dietary choice.

We found that individuals with predominantly Northern European ancestry had significantly higher $\delta^{13}C$ values than individuals with predominantly Southern European ancestry, when looking at the entire adult population together.

Since there was no apparent difference in $\delta^{15}N$ values, we suggest that the differentiating factor was the type of grain - e.g. wheat or barley vs. millet.

Individuals with predominantly Southern European ancestry appear to have followed a more C_3-based diet, whereas individuals with predominantly Northern European ancestry appear to have relied more on C_4 crops.

The $\delta^{13}C$ enriched diet is mostly associated with descendants of the first two clusters of the main Pedigree I, and especially with its later generations, while the majority of individuals with a $\delta^{13}C$ depleted diet either have no kinship relations within the site or belong to Pedigree III, a southern-dominated Pedigree and are mostly associated with later arriving individuals.

It was particularly striking to see that the individuals with the lowest $\delta^{13}C$ (-19.6‰) values have a high proportion of Iberian ancestry in the modern panel (up to 78%) and are amongst the late arriving members of the community.

Further in-depth analysis showed that these late arriving individuals significantly differed from the established population. It is conceivable that the site attracted new people, who in turn would have brought their own dietary practices and preferences with them.

S10.6 Conclusion

The paleodietary analysis of the individuals from Collegno paired with the genetic results revealed complex relationships between biological relatedness, ancestry and diet.

While the same resources might have been available to the people at the time, individuals appear to have made dietary choices that reflect their family and deeper genetic ancestry.

We were also able to identify shifts in dietary patterns. The first shift was characterized by the inclusion of more C_4 plants and dietary protein deriving from C_4-fed animals and can be associated with the later generations of Pedigree I. The second shift appeared to coincide with the arrival of individuals with a higher proportion of the Iberian (IBS) ancestry component and was marked by a notable resurgence in the consumption of larger proportions of C_3 plants. These changes seem to align with different developmental stages of the site.

S11. Statistical tests

Yijie Tian

We employed a permutation test to assess potential differences in central and northern genetic ancestry between Pedigree I and other groups. In each run of the permutation test, the proportions of genetic ancestry from the overall pool are randomly reassigned to individuals without replacement. This process is repeated 10,000 times. In each run, the difference in mean ancestry components between Pedigree I and the other groups was computed, and the p-value was determined by assessing the extent to which the observed difference in means deviates from those obtained in the 10,000 permutations

We utilized Cramér's V test (MASS package) to examine the association between the presence of weapons/elaborate belt sets and individuals in Pedigree I in R. One dimension of the matrix indicates the presence of weapons or belt sets (0 or 1), while the other dimension signifies whether the individual is in Pedigree I (0 or 1).

Two sample t-tests were conducted for other tests using the MASS package in R. For the one-sided test, the command: t.test(A, B, alternative="less") was used.

Supplementary figures

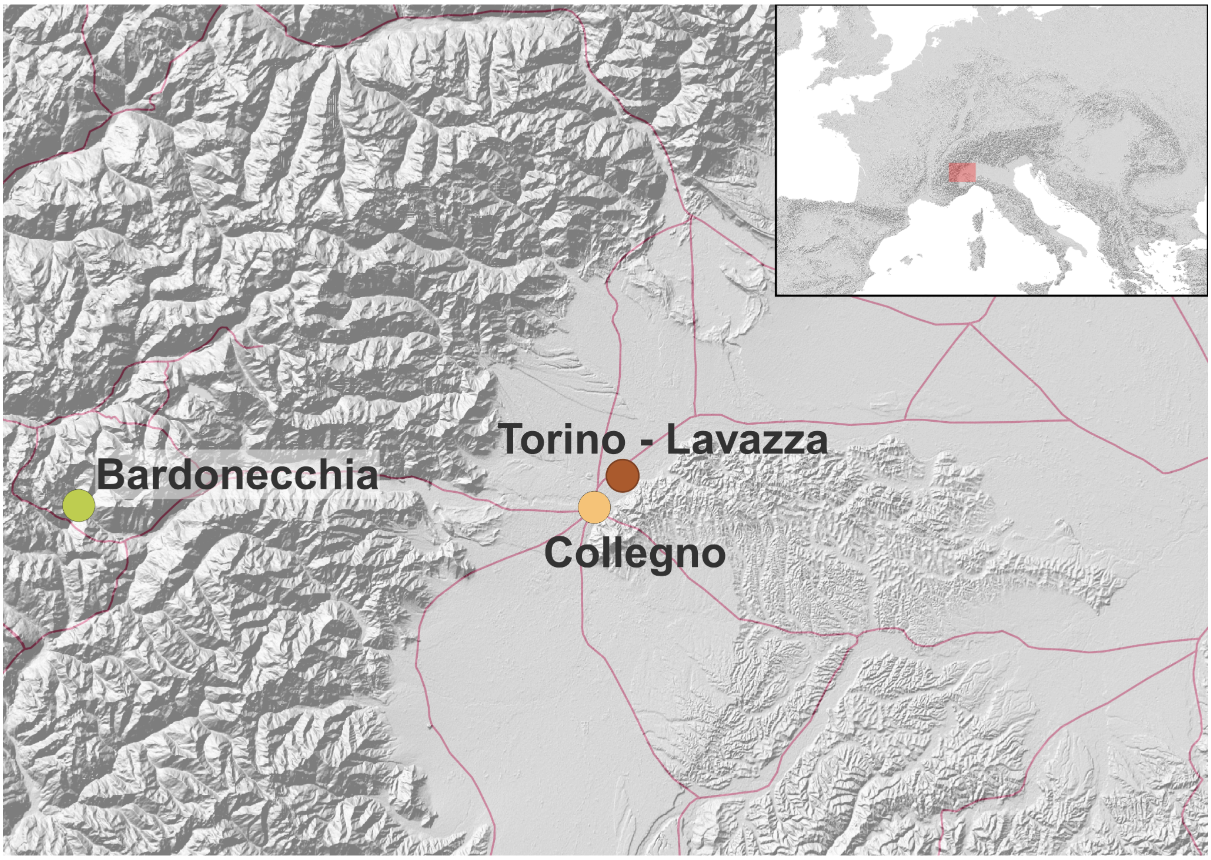

Fig. S1.The geographical locations of Collegno, Torino-Lavazza and Bardonecchia.

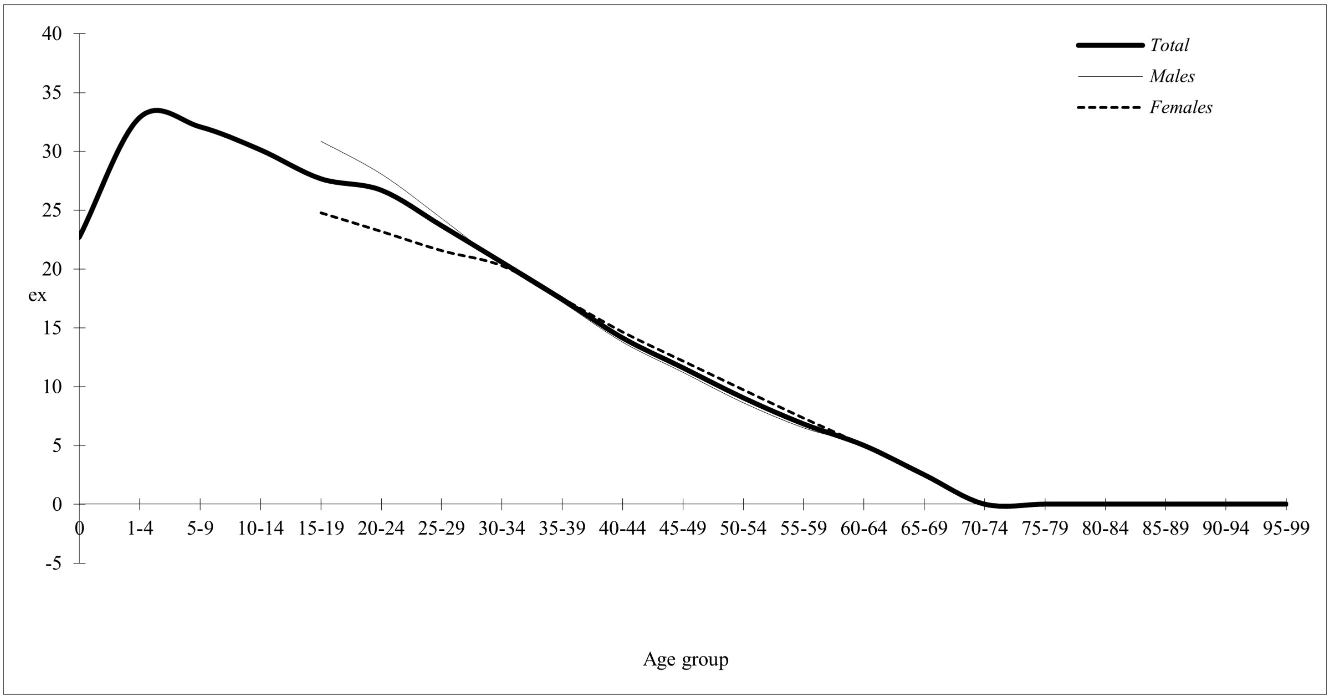

Fig. S2. Life expectancy (ex) at different ages at the Collegno cemetery. The only real difference between males and females shows in young adult (ca. 20-30 years) age.

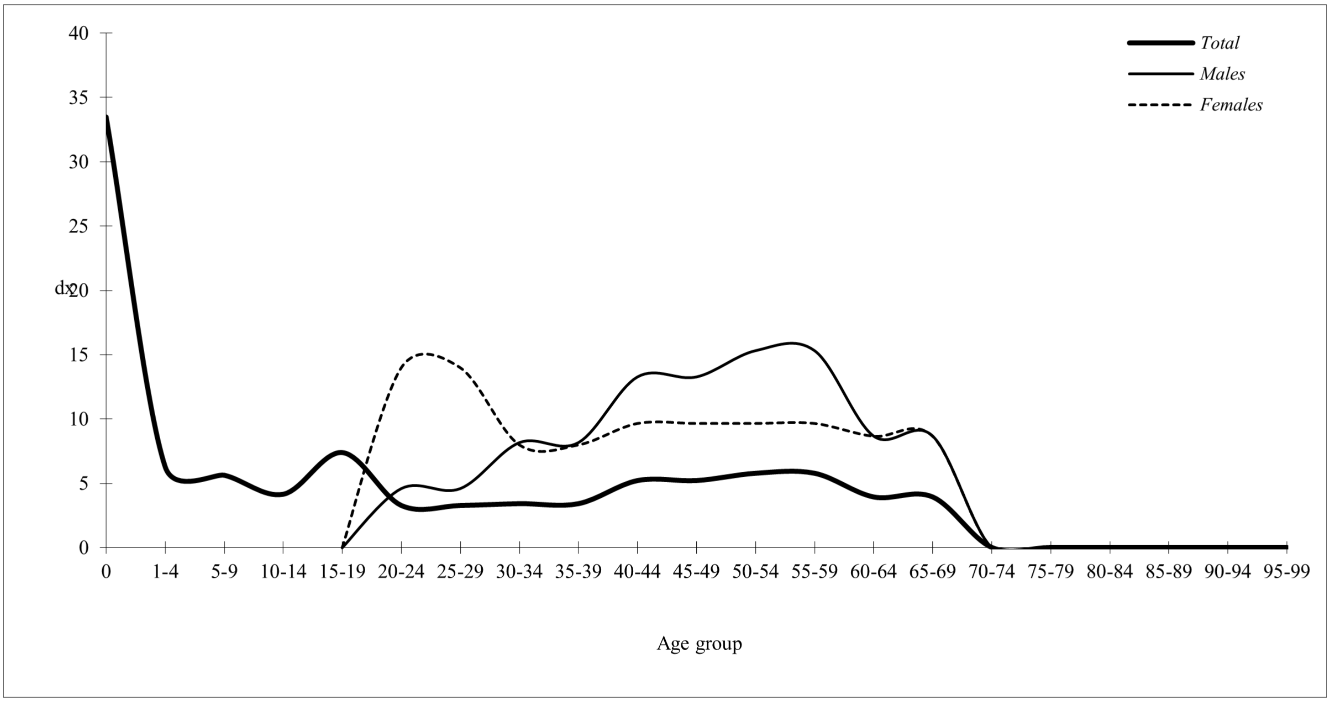

Fig. S3. Mortality curve of the Collegno community with a clear peak among young adults (ca. 20-25 years) females.

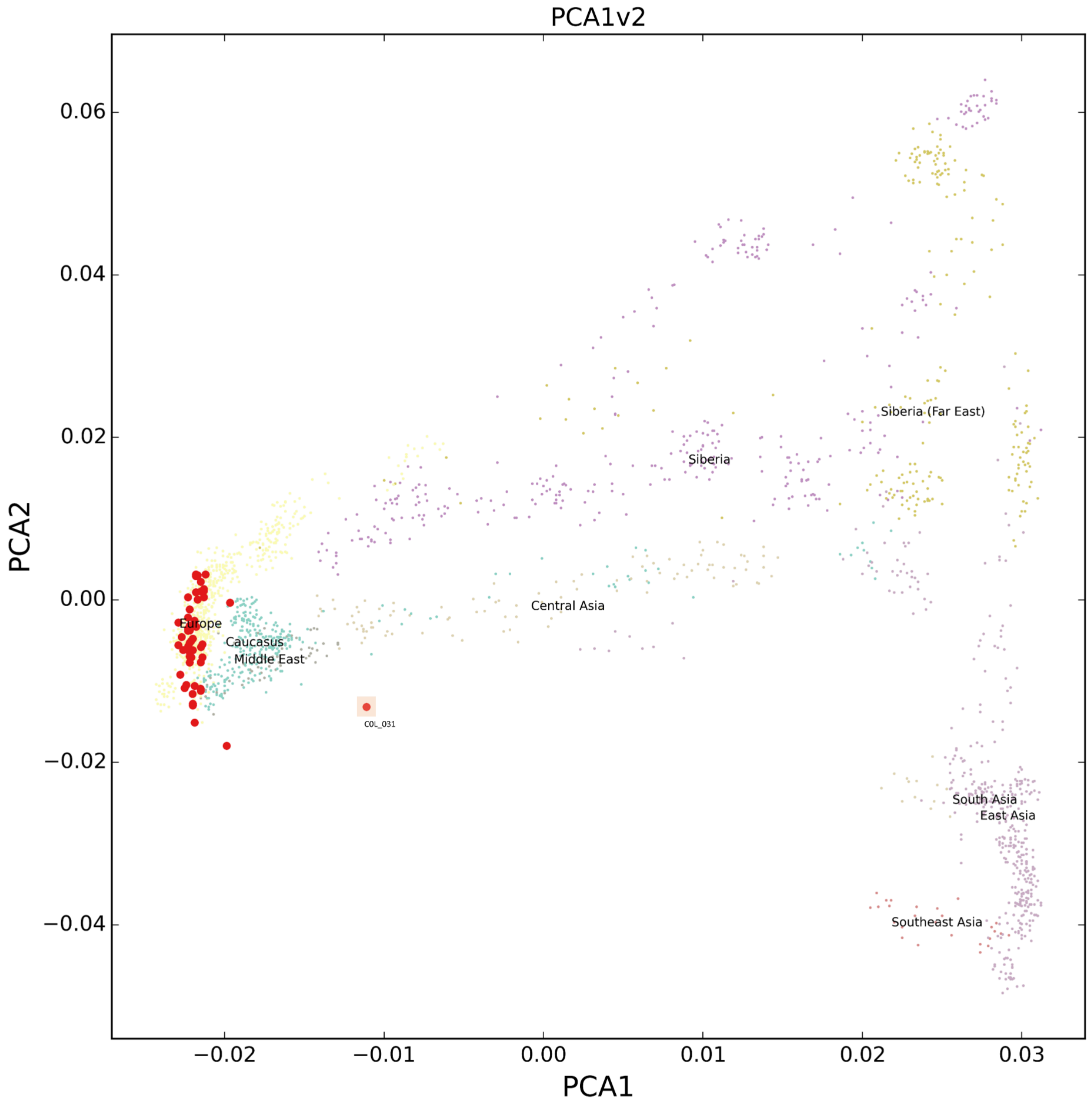

Fig. S4. Procrustes PCA of 52 individuals transformed on to a PCA with 2,209 modern Eurasia individuals from Affymetrix Human Origins array data using pseudohaploid genotype calls from 537,207 SNPs with individuals. Reference individuals are colored in pastel colors based on their region. All the 52 samples are plotted and colored with red. The highly contaminated individual COL_031 is marked with a brown background.

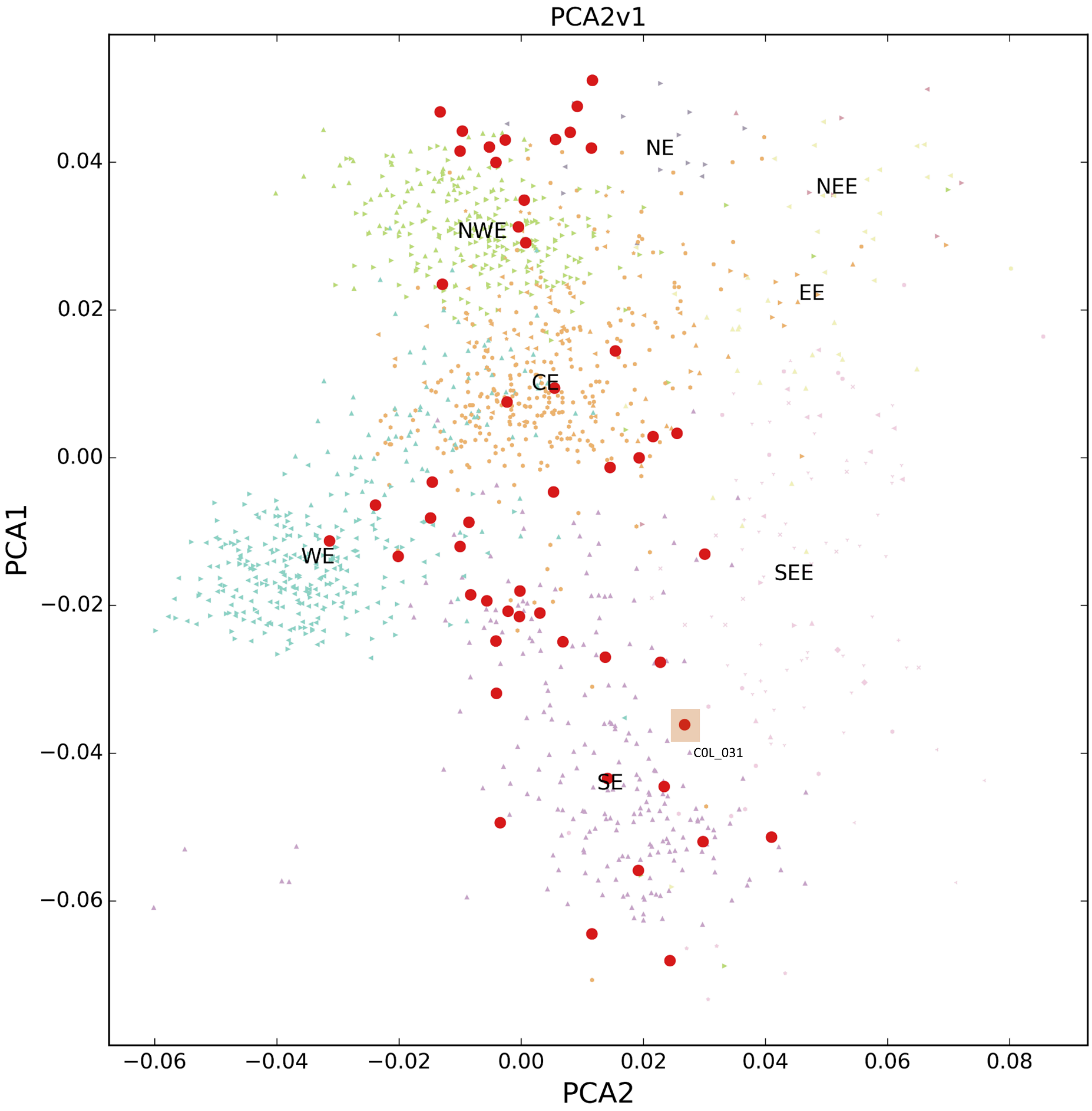

Fig. S5. Procrustes PCA of 52 individuals transformed on to a PCA with 1,385 modern European individuals from the POPRES dataset using pseudohaploid genotype calls from 328,687 SNPs with individuals. Reference individuals are colored in pastel colors based on their region. All the 52 samples excluding COL_110 are plotted and colored with red. The highly contaminated individual COL_031 is marked with a brown background. NE: North Europe, NEE: Northeast Europe, NWE: Northwest Europe, EE: East Europe, CE: central Europe, WE: West Europe, SEE: Southeast Europe, SE: South Europe.

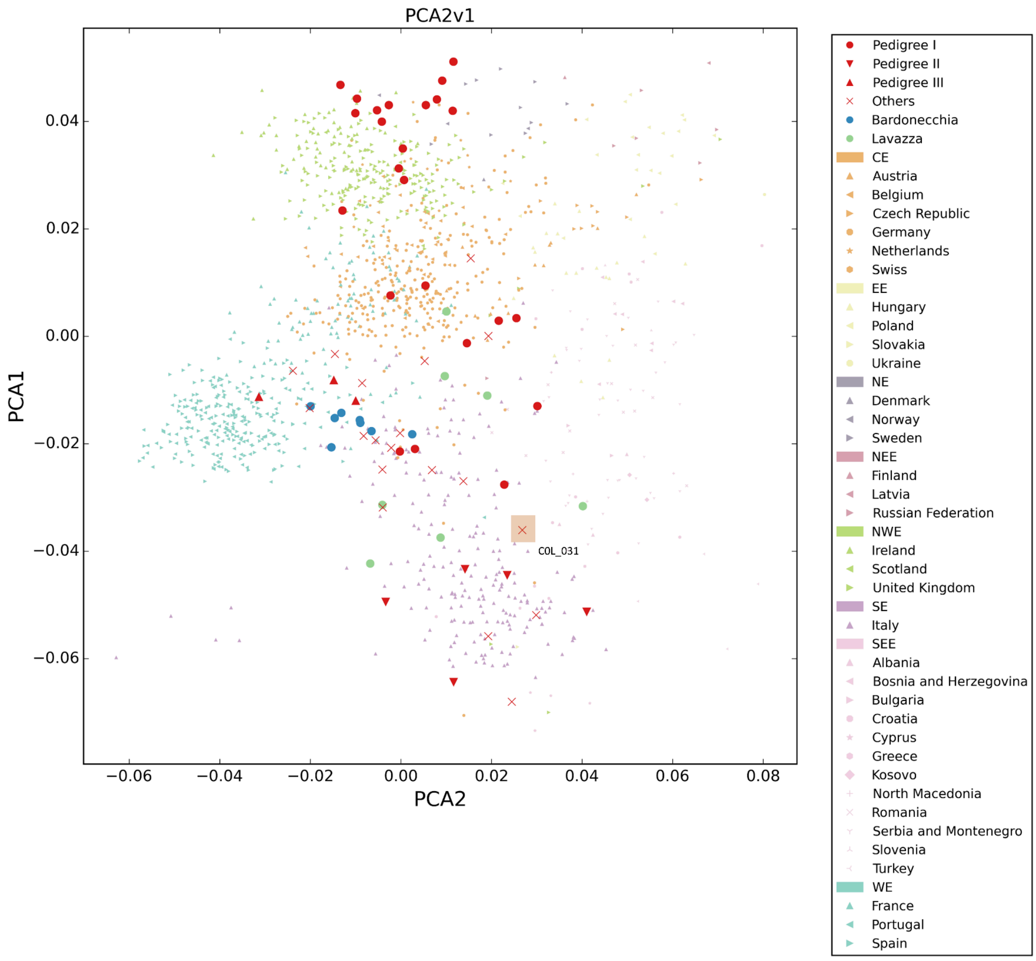

Fig. S6. Procrustes PCA of individuals from Collegno, Bardonecchia, and Torino-Lavazza transformed on to a PCA with 1,385 modern European individuals from the POPRES dataset using pseudohaploid genotype calls from 328,687 SNPs with individuals. Reference individuals are colored in pastel colors based on their region. All the 52 individuals in Collegno excluding COL_110 are plotted and colored with red. The highly contaminated individual COL_031 is marked with a brown background. NE: North Europe, NEE: Northeast Europe, NWE: Northwest Europe, EE: East Europe, CE: Central Europe, WE: West Europe, SEE: Southeast Europe, SE: South Europe.

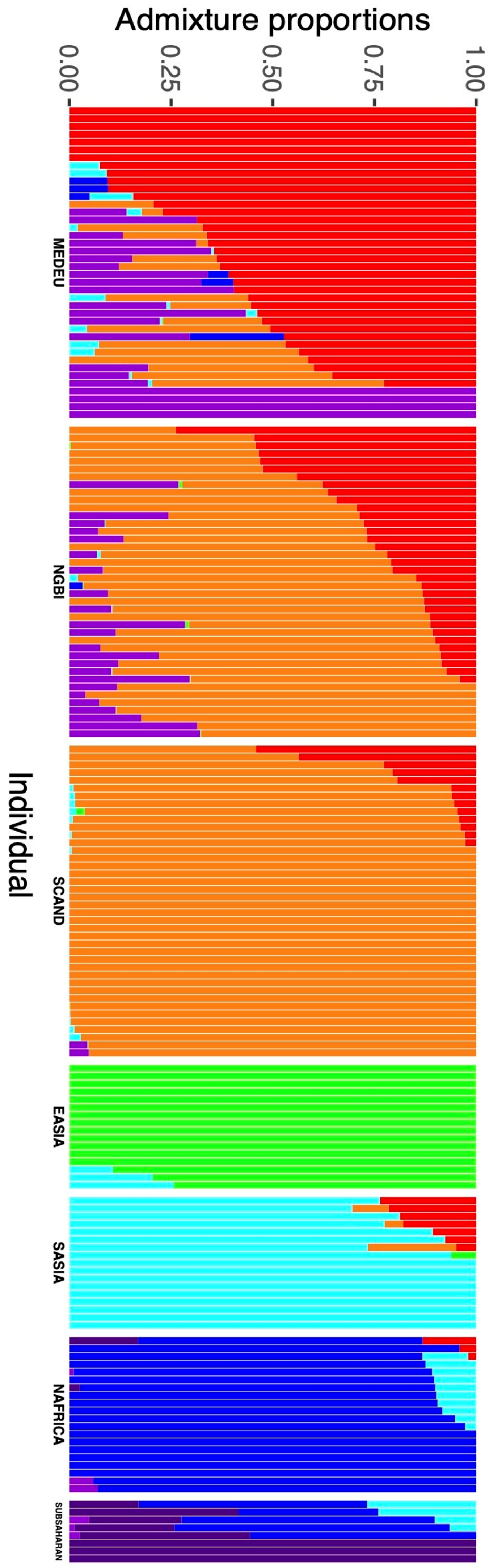

Fig. S7. Unsupervised clustering analysis for the 7 penecontemporaneous populations used in the fastNGSadmix analysis (K=7).

Fig. S8. Unsupervised clustering analysis for the 7 penecontemporaneous populations used in the fastNGSadmix analysis (K=6).

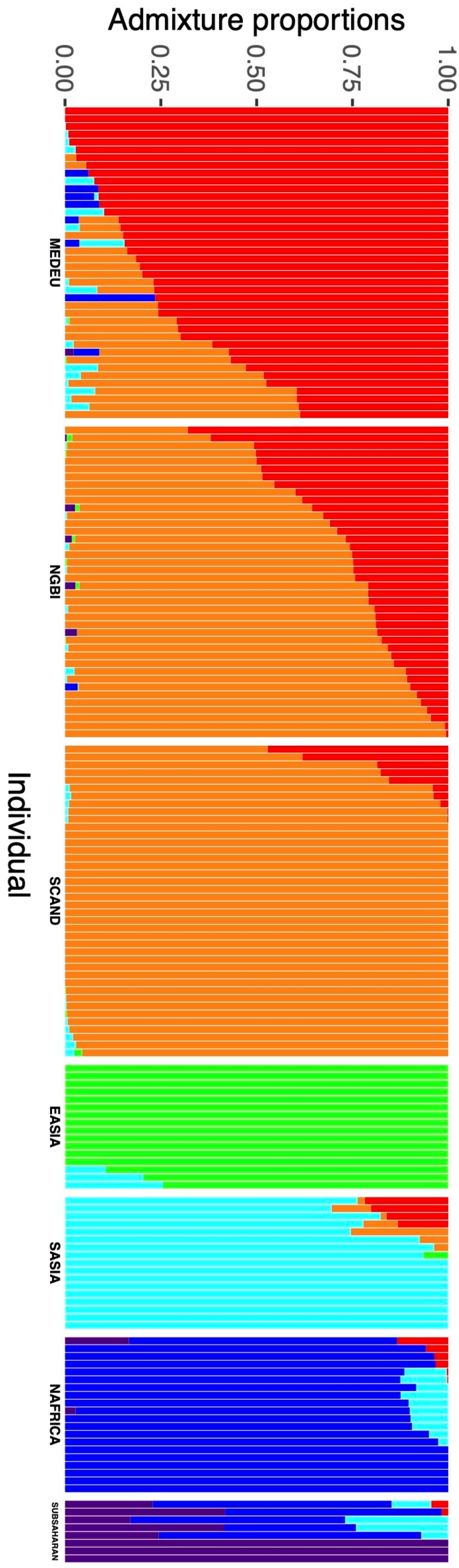

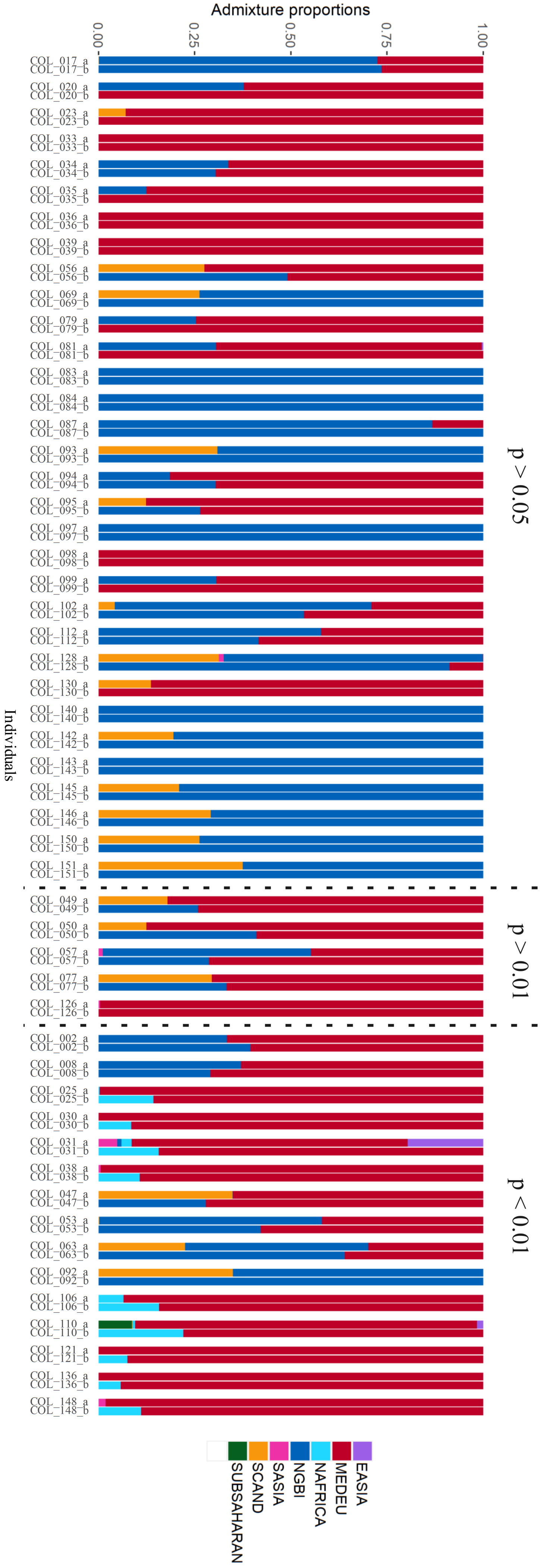

Fig. S9. Comparison between genetic ancestry given by model-based clustering analysis using the penecontemporaneous panel (The names ending with _a) and ancestry proportions given by qpAdm models (The names ending with _b).

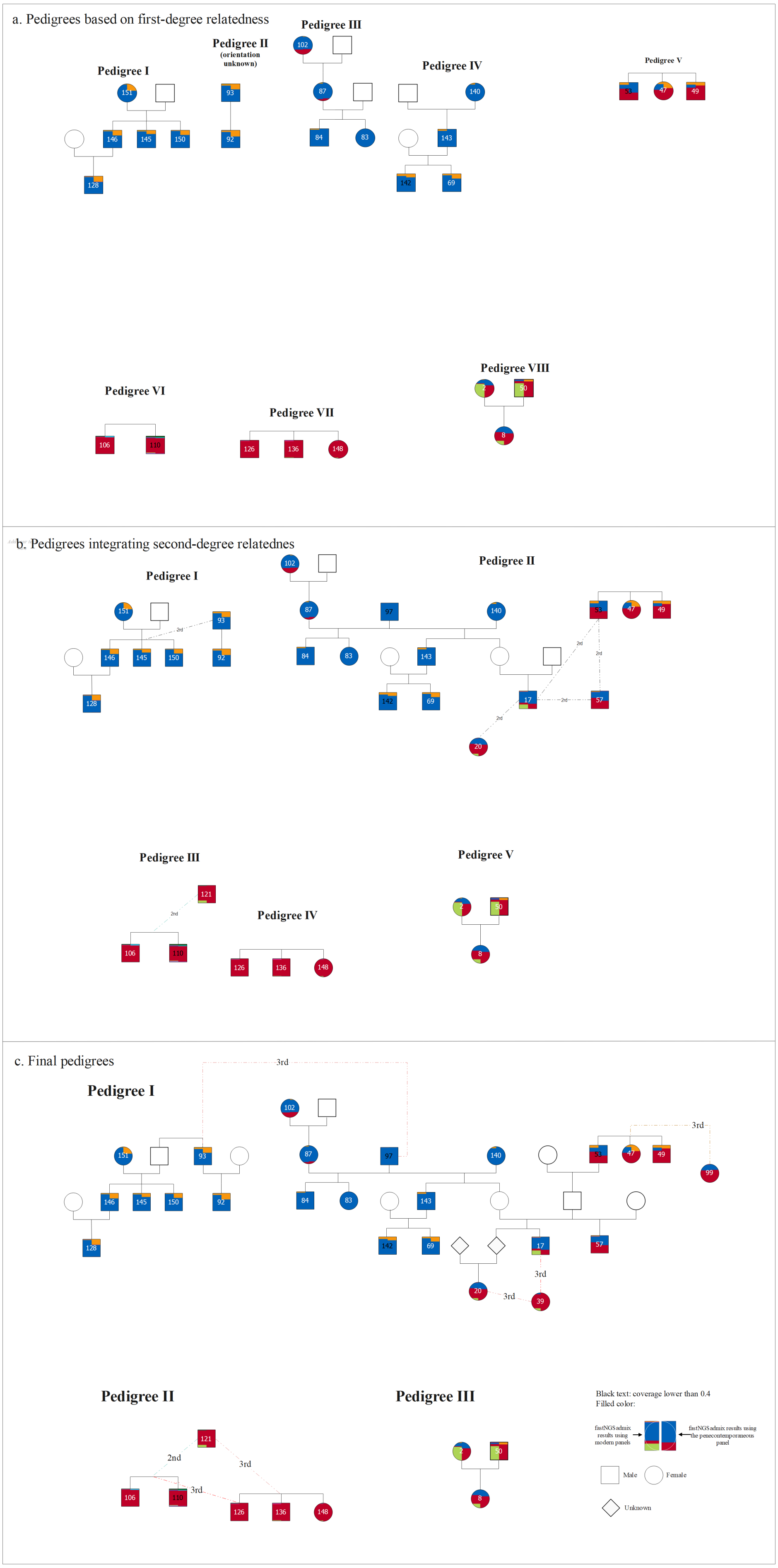

Fig. S10. The process of constructing pedigrees and the intermediate pedigrees. a. Pedigrees based on first-degree relatedness. b. Pedigrees integrating second-degree relatedness. c. The final pedigrees.

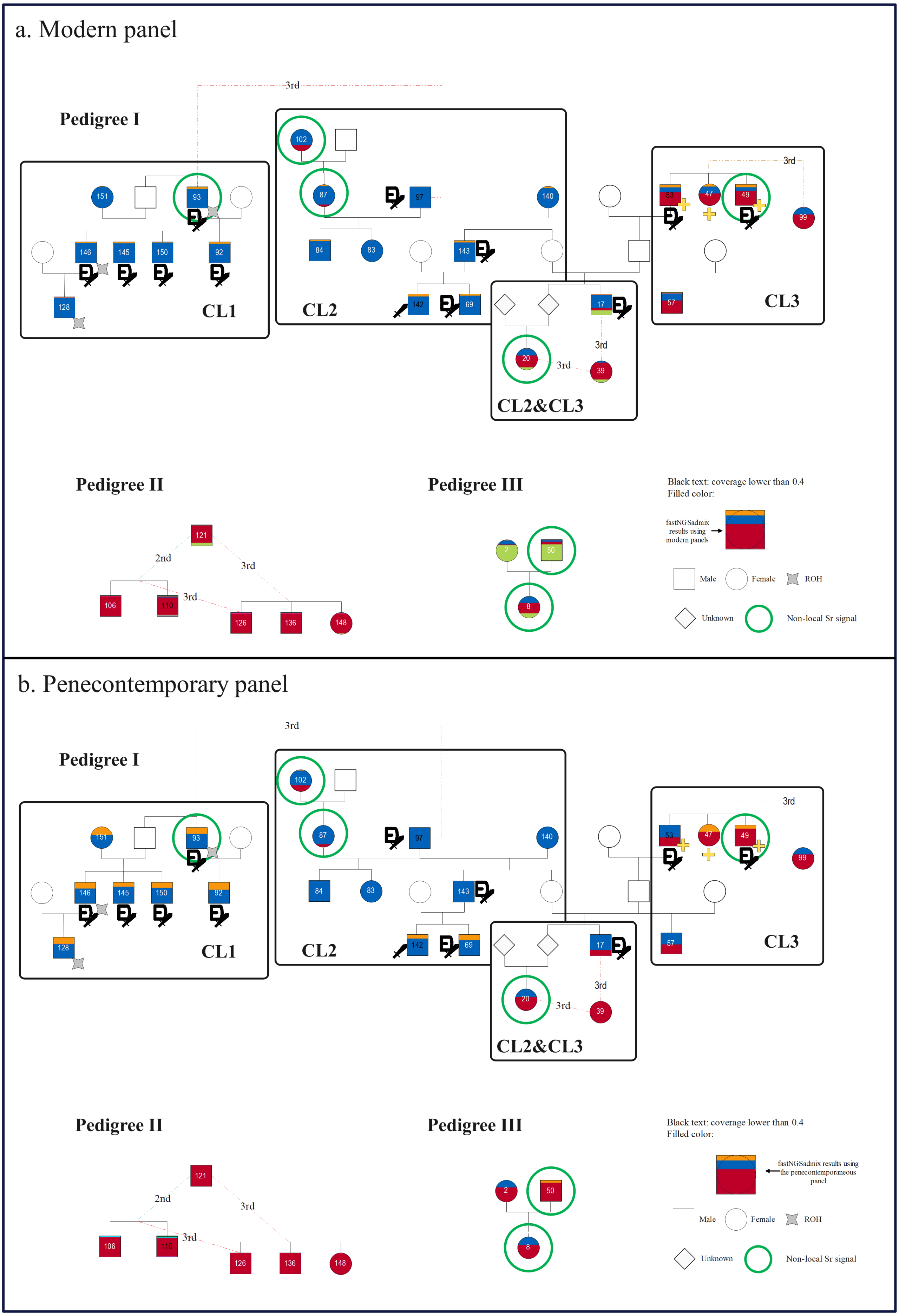

Fig. S11. Pedigrees filled with different colors based on the panel used. a. Modern panel. b. Penecontemporaneous panel.

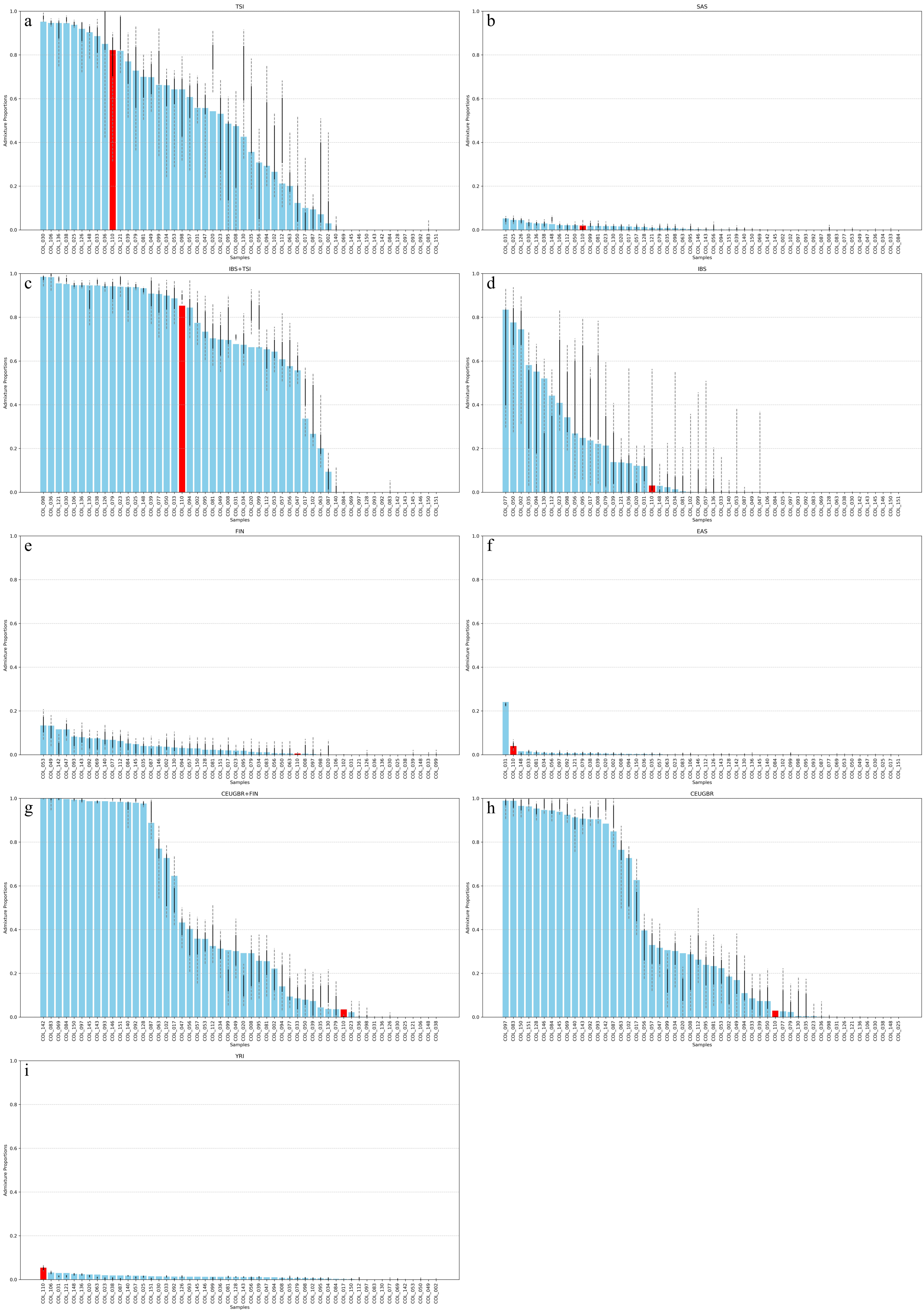

Fig. S12. Block bootstrap results for the modern panel. The 90% confidence intervals (ranging from the 5th to the 95th estimates) are marked using the dashed grey lines, and the 50% confidence intervals (ranging from the 25th to the 75th estimates) are marked with solid black lines. The red bars indicate the individual COL_110 which has the lowest coverage (0.03x).

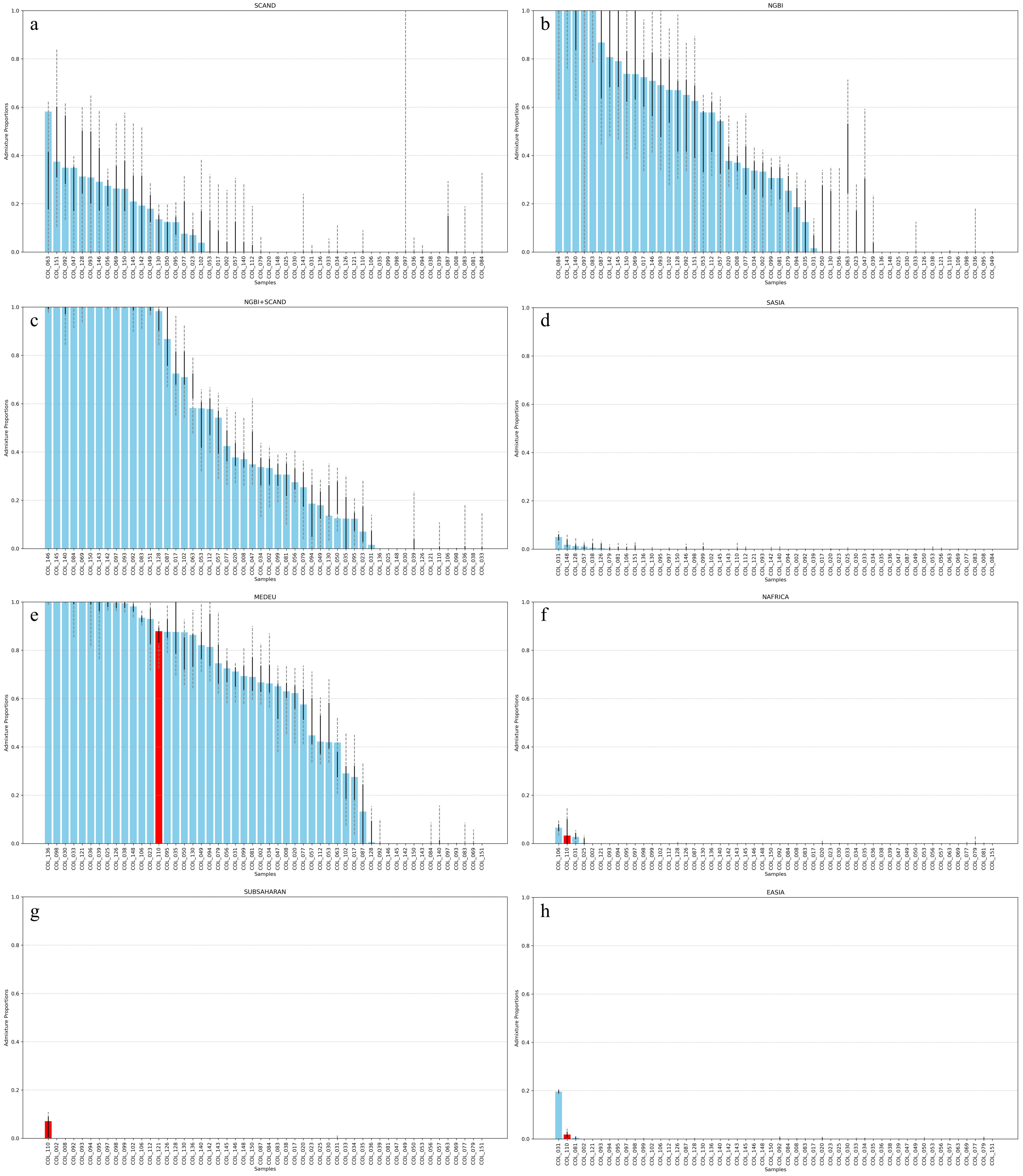

Fig. S13. Block bootstrap results for the penecontemporaneous panel. The 90% confidence intervals (ranging from the 5th to the 95th estimates) are marked using the dashed grey lines, and the 50% confidence intervals (ranging from the 25th to the 75th estimates) are marked with solid black lines. The red bars indicate the individual COL_110 which has the lowest coverage (0.03x).

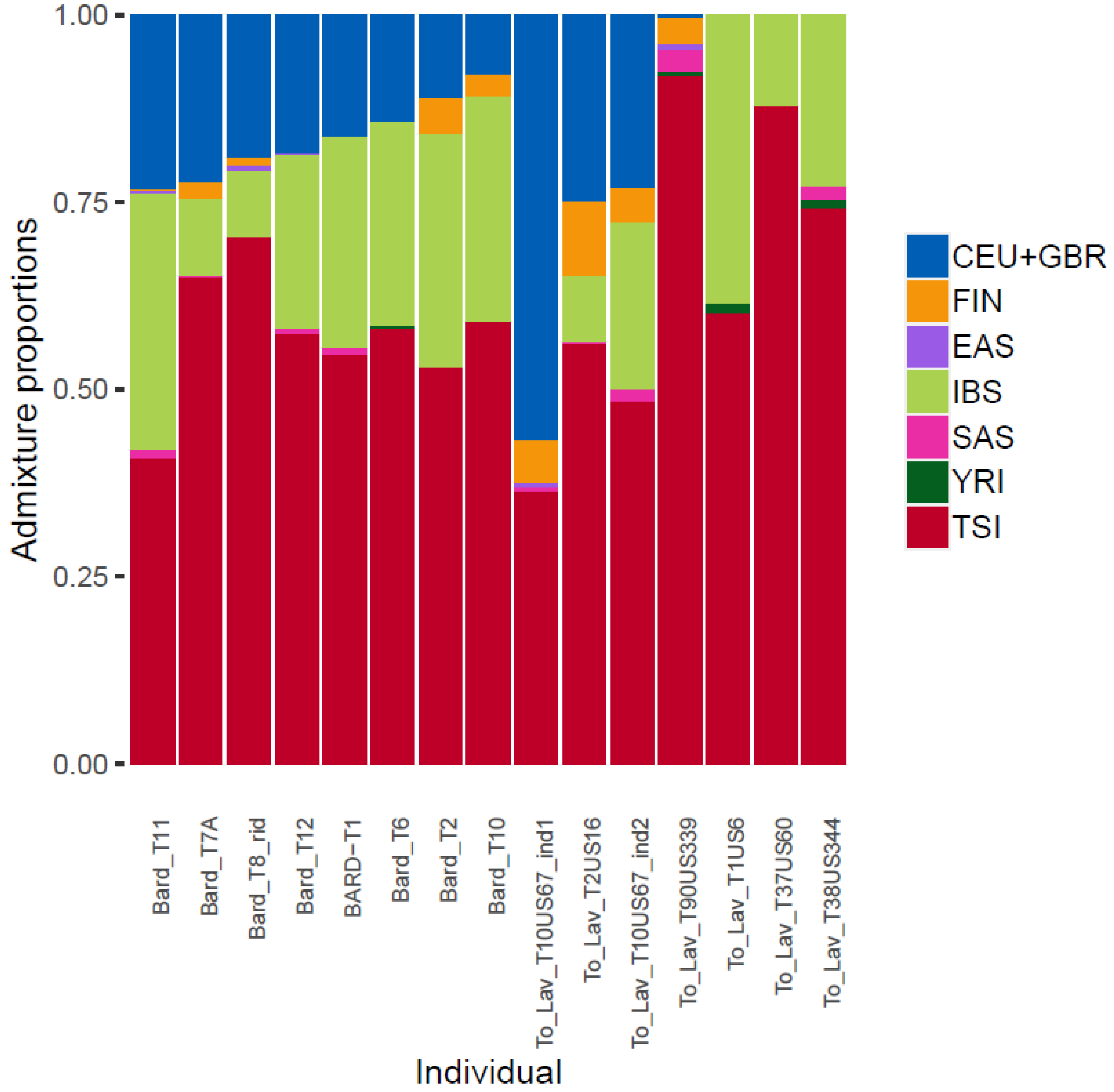

Fig. S14. Model-based clustering analysis of individuals in Bardonecchia (n=8) and Lavazza (n=7). Seven populations from the 1000 Genomes Project (1000G) (1000 Genomes Project Consortium et al. 2015) are used as references as described in ((29)).

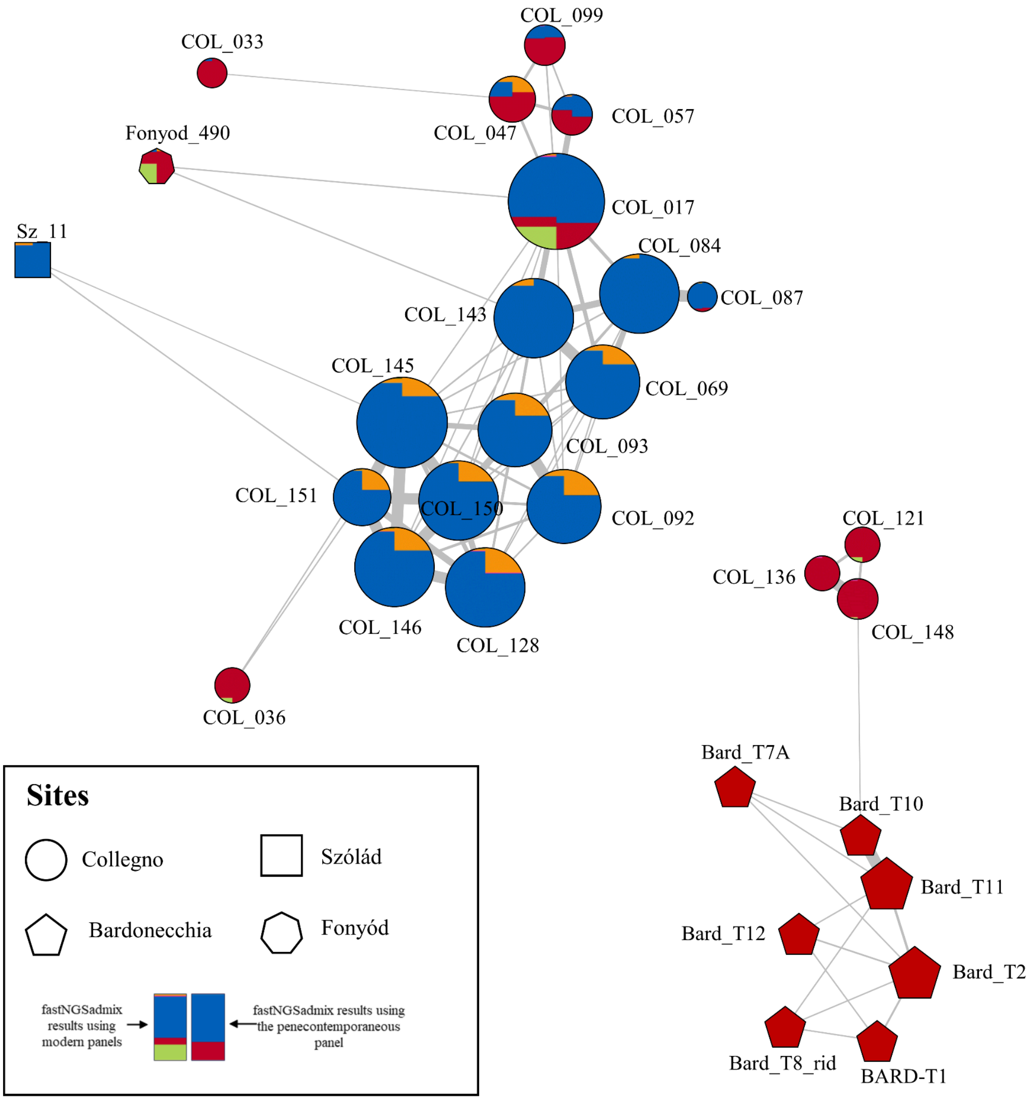

Fig. S15. IBD network based on segments longer than 20cM. Each node in the network represents an individual while each edge represents an IBD connection between two individuals. The edges were weighted by the adjusted Pi-hat value. Due to the large relative differences between the adjusted PI-hat values the edge weights were adjusted to a 0-1 scale. The size of a node represents the number of IBD connections it has. The filled colors of each node indicate the fastNGSadmix results of both the modern and penecontemporaneous panels.

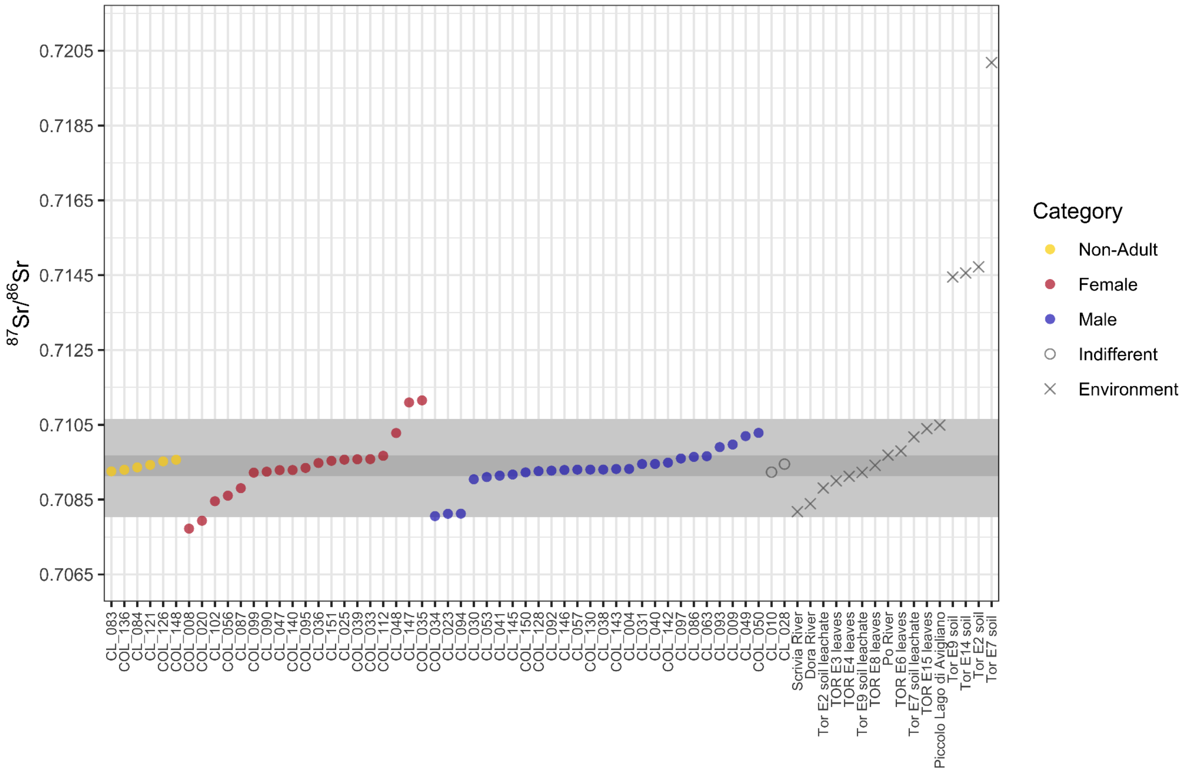

Fig. S16. Human and environmental Sr values from Collegno. The ‘wider local’ range based on the double standard deviation of all human values is marked with light gray, the ‘narrow local’ range based on the double standard deviation of non-adult values is marked with dark gray. Adults without either genetic or osteological sex estimation are marked as indifferent.

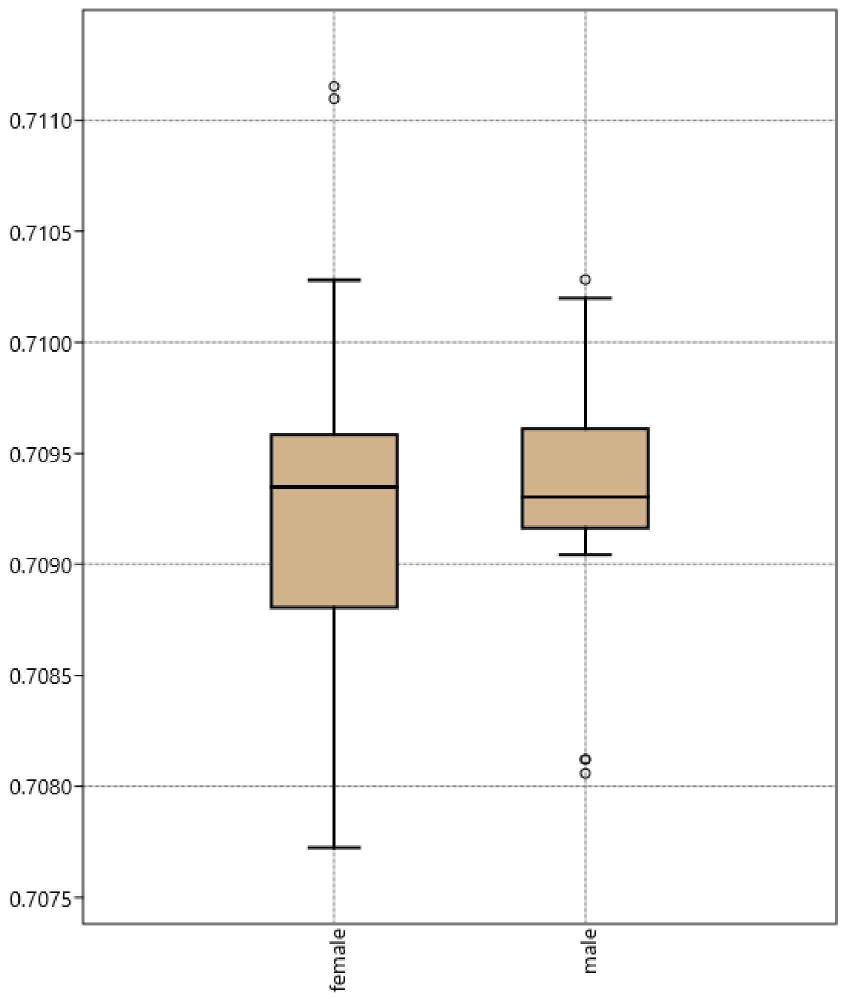

Fig. S17. Comparison of Sr values of adult females and adult males at Collegno.

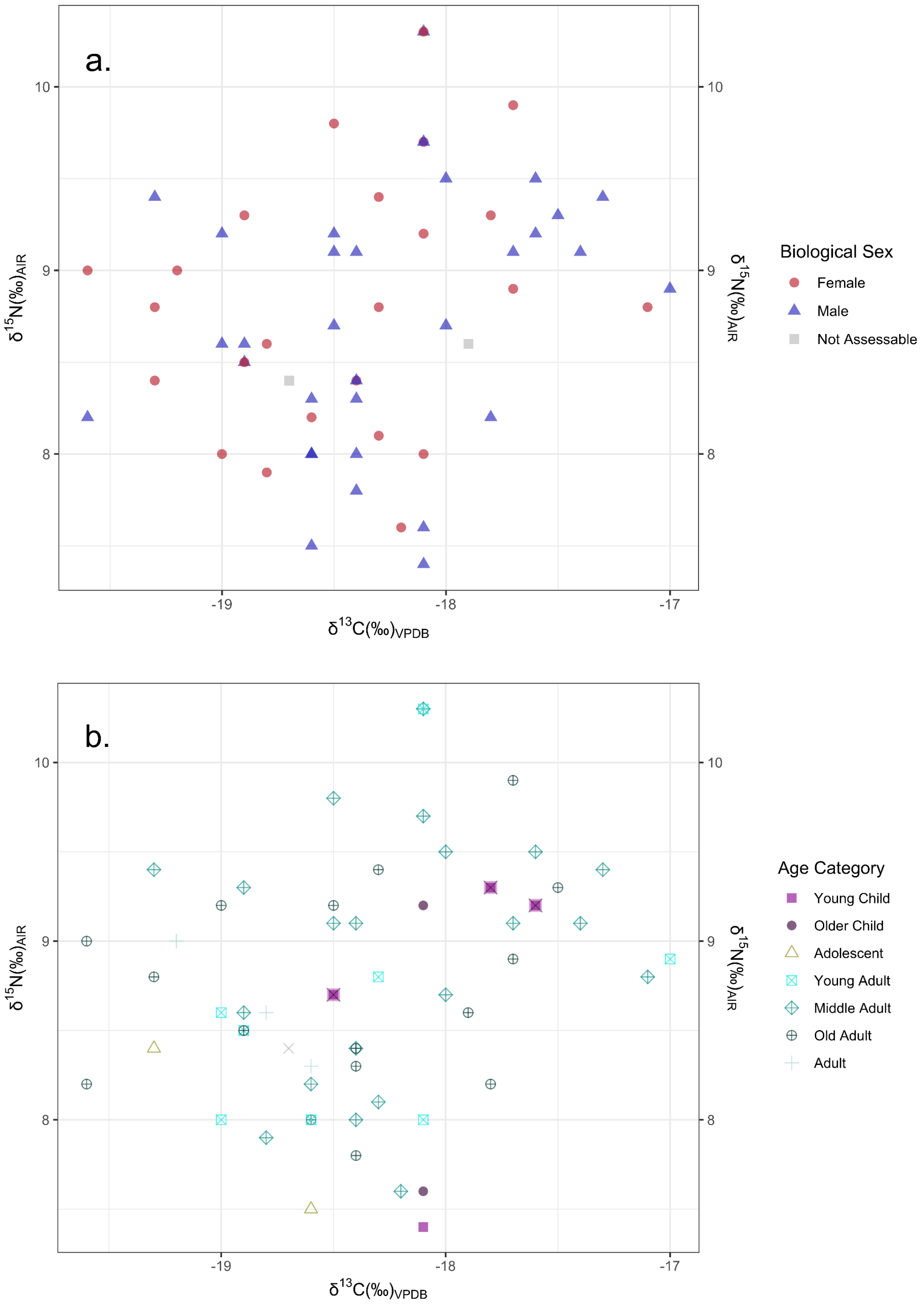

Fig. S18. Distribution of biological sexes (a) and age-at-death categories (b). Note: Young Children under the age of 3 years or without specified age are highlighted.

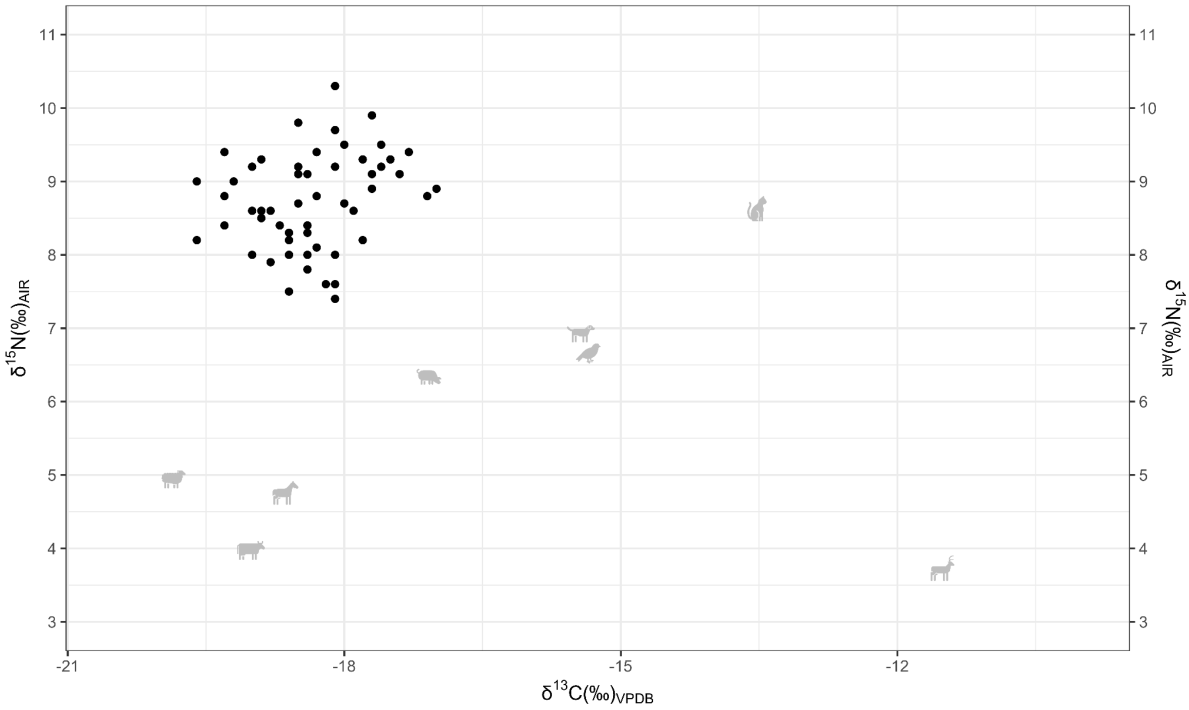

Fig. S19. Human values from Collegno with faunal data from the Bergamo region (65)

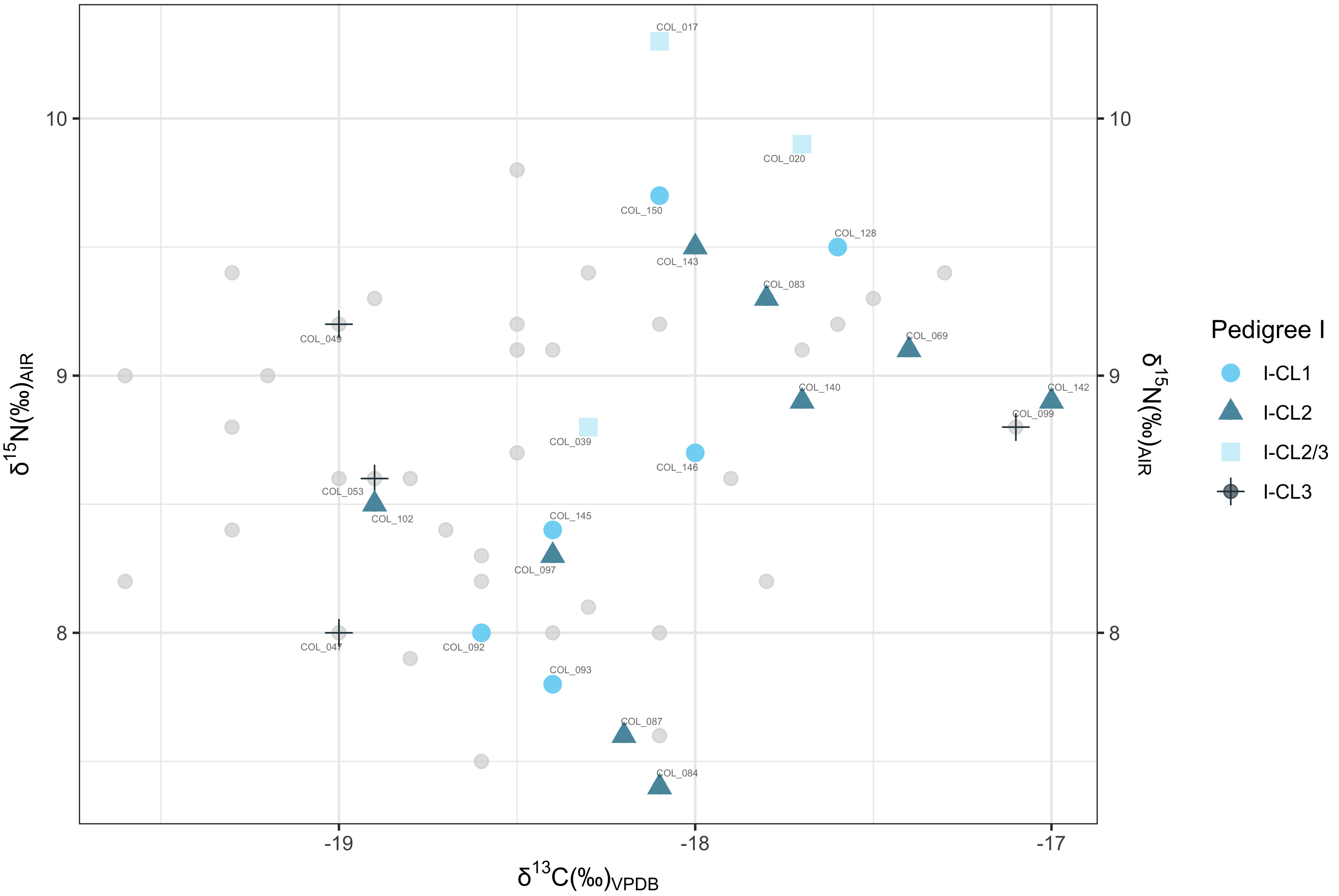

Fig. S20. Palaeodietary data of Pedigree I and its clusters.

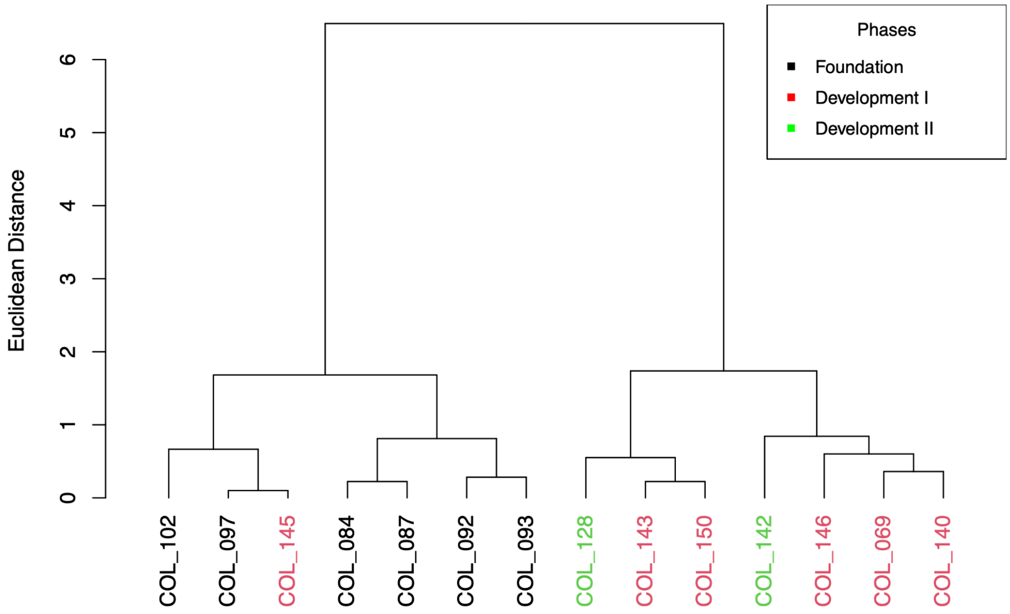

Fig. S21. Hierarchical Cluster Analysis of I-CL1 and I-CL2 based on developmental phases of the site.

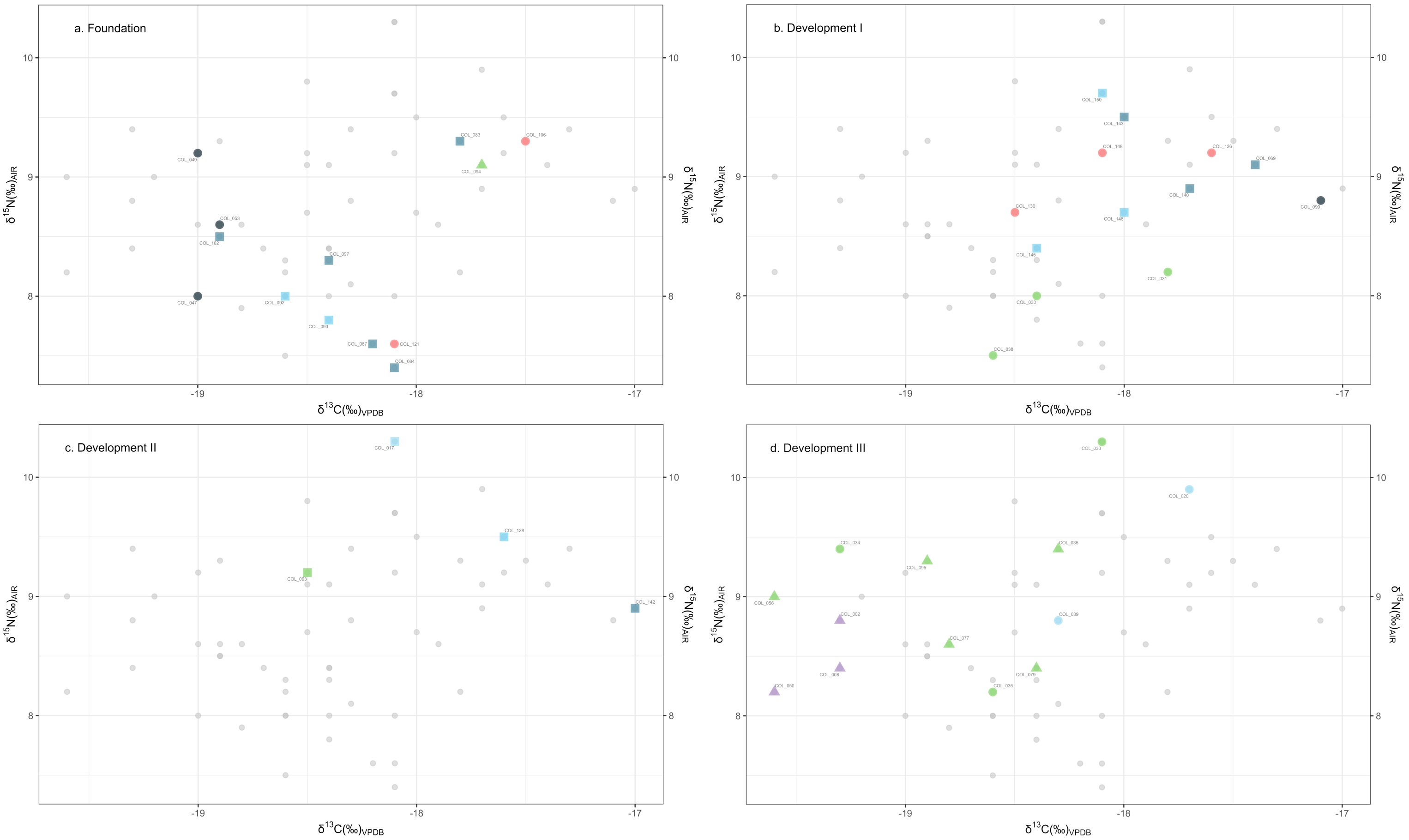

Fig. S22. a-d: Changing dietary patterns within the population of Collegno. Colors represent the different Pedigrees (shades of blue (see FigS20): Pedigree I (CL1, CL2, CL3, CL2/3), red: Pedigree II, purple: Pedigree III, green: unrelated), while shapes represent main Ancestry component (triangle = Iberian (IBS), round = Southern (e.g. TSI), square = Northern (e.g. CEU+GBR)).

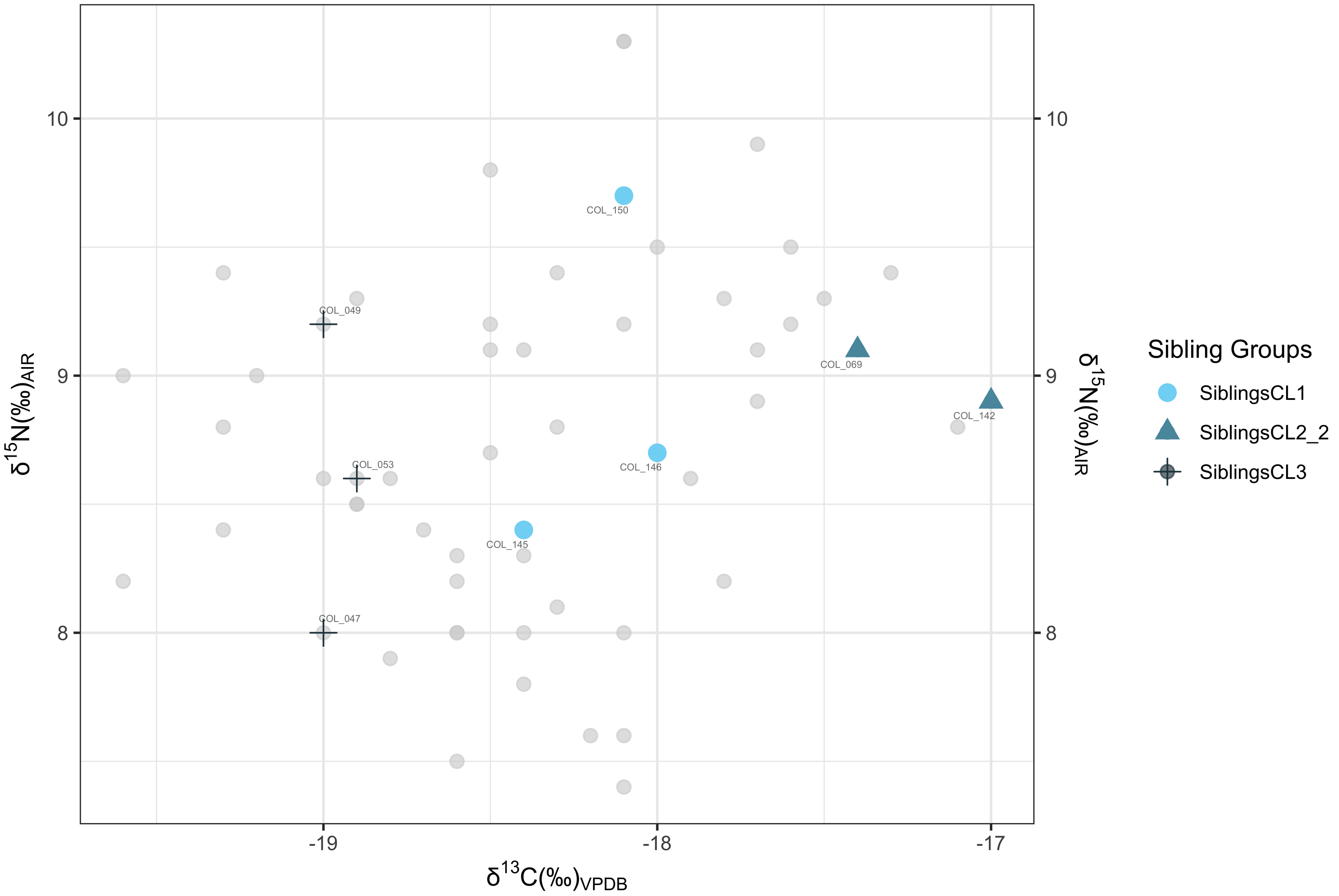

Fig. S23. Plot showing dietary values of adult sibling groups from Collegno.

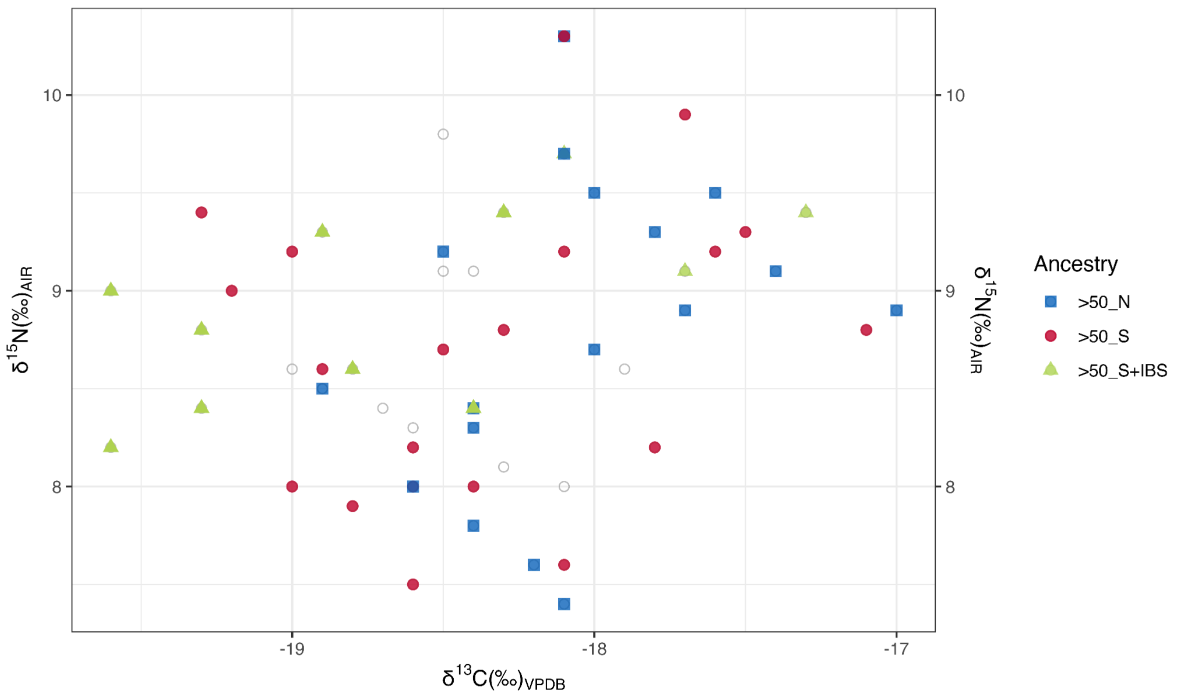

Fig. S24. Dietary values of individuals at Collegno based on their main ancestry component in the modern admixture panel.

Supplementary Tables

Table S1. Bootstrapping results for ancestry components in the model panel used in fastNGSadmix

Component	TSI(%)	YRI(%)	SAS(%)	IBS(%)	EAS(%)	FIN(%)	CEUGBR(%)	CEUGBR+FIN(%)	IBS+TSI
mean sd	9.322	0.3116	0.6753	9.766	0.2862	2.096	5.258	4.328	4.402
max sd	21.53	1.000	1.530	25.26	1.582	4.413	12.54	12.50	12.72
ind with max sd (Coverage)	COL_110 (0.03x)	COL_110 (0.03x)	COL_110 (0.03x)	COL_095 (0.75x)	COL_110 (0.03x)	COL_053 (0.38x)	COL_102 (0.50x)	COL_102 (0.50x)	COL_102 (0.50x)

Table S2. Bootstrapping results for ancestry components in the penecontemporaneous panel used in fastNGSadmix

Component	EASIA(%)	MEDEU(%)	NAFRICA(%)	NGBI(%)	SASIA(%)	SCAND(%)	SUBSAHARAN(%)	NGBI+SCAND(%)
mean sd	0.1243	6.020	0.3066	11.89	0.4651	8.106	0.1010	5.793
max sd	1.478	11.88	5.690	27.20	19.65	27.08	3.950	11.68
ind with max sd (Coverage)	COL_110 (0.03x)	COL_057 (1.67x)	COL_110 (0.03x)	COL_097 (0.15x)	COL_148 (1.26x)	COL_097(0.15x)	COL_110 (0.03x)	COL_087 (3.19x)

Table S3. Environmental Strontium isotope values (29)

Sample ID	Location	87Sr/86Sr	Lat	Long	Type	Environmental context
**TOR E5**	Lago Piccolo di Avigliana	0.710492	45.050341	7.3945391	water	lake shore
**TOR E12**	Dora (Turin)	0.708390	45.081358	7.685964	water	urban
**TOR E13**	Po (Turin)	0.709689	45.069733	7.720191	water	urban
**TOR E2**	Parco Generale dall Chiesa, Collegno	0.708809	45.079862	7.577408	soil	civic park
**TOR E3**	Parco Generale dall Chiesa, Collegno	0.709001	45.079643	7.577606	Tilia cordata leaf	civic park
**TOR E4**	Parco Generale dall Chiesa, Collegno	0.709123	45.079837	7.576593	Celtis australis leaf	civic park
**TOR E6**	Lago Piccolo di Avigliana	0.709800	45.050341	7.394539	Carpinus betulus leaf	lake shore
**TOR E7**	Lago Piccolo di Avigliana	0.710176	45.050086	7.394854	soil	meadows
**TOR E8**	Castello di Avigliana	0.709416	45.079222	7.390282	Quercus leaf	top of rocky outcrop
**TOR E9**	Castello di Avigliana	0.709227	45.079394	7.389884	soil	pasture
**TOR E14**	Collina di Superga	0.710312	45.061383	7.775918	soil	deciduous forests, no ground cover
**TOR E15**	Collina di Superga	0.710398	45.061383	7.775918	Carpinus betulus leaf	deciduous forests, no ground cover

Table S4. Summary statistics of $\delta^{13}C_{(collagen)}$ values from penecontemporaneous animals from the province of Bergamo (after (65)).

Taxon	Number of Samples	Mean $\delta^{13}C$	SD	Min	Max
**Birds**	2 galliformes, 3 unspecified	-15.35	3.53	-18.73	-11.37
**Horse**	4	-18.64	1.58	-20.66	-16.85
**Ovicaprid**	5	-19.85	0.76	-20.41	-18.52
**Cattle**	6	-19.01	1.86	-20.45	-15.39
**Pig**	4	-17.07	2.38	-19.58	-14.43
**Dog**	1*	-16.96	NA	NA	NA
**Cat**	1	-13.52	NA	NA	NA
**Deer**	1	-11.51	NA	NA	NA

Table S5. Summary statistics of $\delta^{15}N_{(collagen)}$ values from penecontemporaneous animals from the province of Bergamo (after(65)).

Taxon	Number of Samples	Mean $\delta^{15}N$	SD	Min	Max
**Birds**	2 galliformes, 3 unspecified	6.70	0.71	6.13	7.86
**Horse**	4	4.80	1.66	3.67	7.27
**Ovicaprid**	5	4.98	1.26	3.67	6.85
**Cattle**	6	4.01	1.19	2.94	6.04
**Pig**	4	6.38	0.56	5.84	7.15
**Dog**	1*	5.84	NA	NA	NA
**Cat**	1	8.67	NA	NA	NA
**Deer**	1	3.77	NA	NA	NA

Table S6. *S*ummary statistics of $\delta^{13}C$ values in adult individuals grouped by their biological sex (genetic where available, otherwise based on osteological assessment (see Dataset S1)).

Sex	N	Mean	SD	Median	Min	Max	Range
**Female**	22	-18.51	0.60	-18.45	-19.60	-17.10	2.50
**Male**	28	-18.31	0.62	-18.40	-19.60	-17.00	2.60

Table S7. Summary Statistics of $\delta^{15}N$ values in adult individuals grouped by their biological sex (genetic where available, otherwise based on osteological assessment (see Dataset 1)).

Sex	N	Mean	SD	Median	Min	Max	Range
**Female**	22	8.79	0.72	8.80	7.60	10.30	2.70
**Male**	28	8.78	0.66	8.80	7.50	10.30	2.80

Table S8. Summary statistics of $\delta^{13}C$ values of individuals grouped by their Pedigree.

Pedigree	Cluster	N	Mean	SD	Median	Min	Max	Range
**I**	-	22	-18.12	0.57	-18.10	-19.00	-17.00	2.00
	CL1	6	-18.18	0.36	-18.25	-18.60	-17.60	1.00
	CL2	9	-17.94	0.56	-18.00	-18.90	-17.00	1.90
	CL3	4	-18.50	0.93	-18.95	-19.00	-17.10	1.90
	CL2/3	3	-18.03	0.31	-18.10	-18.30	-17.70	0.60
**II**	-	5	-17.96	0.41	-18.10	-18.50	-17.50	1.00
**III**	-	3	-19.40	0.17	-19.40	-19.60	-19.30	0.30
**Unrelated**	-	18	-18.50	0.58	-18.55	-19.60	-17.30	2.30
**No aDNA**	-	10	-18.49	0.34	-18.50	-19.00	-17.90	1.10

Table S9. Summary statistics of δ^{15}N values of individuals grouped by their Pedigrees.

Pedigree	Cluster	N	Mean	SD	Median	Min	Max	Range
**I**	-	22	8.78	0.76	8.80	7.40	10.30	2.90
	CL1	6	8.68	0.78	8.55	7.80	9.70	1.90
	CL2	9	8.61	0.73	8.90	7.40	9.50	2.10
	CL3	4	8.65	0.50	8.70	8.80	9.20	1.20
	CL2/3	3	9.67	0.78	9.90	8.80	10.30	1.50
**II**	-	5	8.80	0.71	9.20	7.60	9.30	1.70
**III**	-	3	8.74	0.31	8.40	8.20	8.80	0.60
**Unrelated**	-	18	8.81	0.74	9.0	7.50	10.30	2.80
**No aDNA**	-	10	8.65	0.54	8.55	8.00	9.80	1.80

SI References

1. L. Pejrani Baricco, Ed., *Presenze longobarde: Collegno nell'alto Medioevo* (Soprintendenza per i Beni Archeologici del Piemonte, 2004).

2. E. Bedini, F. Bertoldi, "Aspetto fisico, stile di vita e stato di salute del gruppo umano" in *Presenze Longobarde: Collegno Nell'alto Medioevo*, L. Pejrani Baricco, Ed. (Soprintendenza per i Beni Archeologici del Piemonte, 2004), pp. 217–236.

3. M. Margulies, *et al.*, Genome sequencing in microfabricated high-density picolitre reactors. *Nature* **437**, 376–380 (2005).

4. Q. Fu, *et al.*, An early modern human from Romania with a recent Neanderthal ancestor. *Nature* **524**, 216–219 (2015).

5. I. Lazaridis, *et al.*, Genomic insights into the origin of farming in the ancient Near East. *Nature* **536**, 419–424 (2016).

6. I. Lazaridis, *et al.*, Ancient human genomes suggest three ancestral populations for present-day Europeans. *Nature* **513**, 409–413 (2014).

7. S. A. Biagini, *et al.*, People from Ibiza: an unexpected isolate in the Western Mediterranean. *Eur. J. Hum. Genet.* **27**, 941–951 (2019).

8. C.-C. Wang, *et al.*, Genomic insights into the formation of human populations in East Asia. *Nature* **591**, 413–419 (2021).

9. N. Patterson, *et al.*, Ancient admixture in human history. *Genetics* **192**, 1065–1093 (2012).

10. C. Jeong, *et al.*, The genetic history of admixture across inner Eurasia. *Nature Ecology & Evolution* **3**, 966–976 (2019).

11. P. Flegontov, *et al.*, Palaeo-Eskimo genetic ancestry and the peopling of Chukotka and North America. *Nature* **570**, 236–240 (2019).

12. ,Allen Ancient DNA Resource v50.0 (2021).

13. M. R. Nelson, *et al.*, The Population Reference Sample, POPRES: a resource for population, disease, and pharmacological genetics research. *Am. J. Hum. Genet.* **83**, 347–358 (2008).

14. K. R. Veeramah, *et al.*, Population genomic analysis of elongated skulls reveals extensive female-biased immigration in Early Medieval Bavaria. *Proc. Natl. Acad. Sci. U. S. A.* **115**, 3494–3499 (2018).

15. A. L. Price, *et al.*, Principal components analysis corrects for stratification in genome-wide association studies. *Nat. Genet.* **38**, 904–909 (2006).

16. N. Patterson, A. L. Price, D. Reich, Population structure and eigenanalysis. *PLoS Genet.* **2**, e190 (2006).

17. D. H. Alexander, J. Novembre, K. Lange, Fast model-based estimation of ancestry in unrelated individuals. *Genome Res.* **19**, 1655–1664 (2009).

18. D. N. Vyas, *et al.*, Fine-scale sampling uncovers the complexity of migrations in 5th-6th century Pannonia. *Curr. Biol.* (2023) https:/doi.org/10.1016/j.cub.2023.07.063.

19. W. Haak, *et al.*, Massive migration from the steppe was a source for Indo-European languages in Europe. *Nature* **522**, 207–211 (2015).

20. I. Mathieson, *et al.*, Genome-wide patterns of selection in 230 ancient Eurasians. *Nature* **528**, 499–503 (2015).

21. M. E. Allentoft, *et al.*, Population genomics of Bronze Age Eurasia. *Nature* **522**, 167–172 (2015).

22. F. Broushaki, *et al.*, Early Neolithic genomes from the eastern Fertile Crescent. *Science* **353**, 499–503 (2016).

23. C. Gamba, *et al.*, Genome flux and stasis in a five millennium transect of European prehistory. Nat Commun 5: 5257 (2014).

24. Z. Hofmanová, *et al.*, Early farmers from across Europe directly descended from Neolithic Aegeans. *Proc. Natl. Acad. Sci. U. S. A.* **113**, 6886–6891 (2016).

25. I. Mathieson, *et al.*, The genomic history of southeastern Europe. *Nature* **555**, 197–203 (2018).

26. I. Olalde, *et al.*, Derived immune and ancestral pigmentation alleles in a 7,000-year-old Mesolithic European. *Nature* **507**, 225–228 (2014).

27. M. van de Loosdrecht, *et al.*, Pleistocene North African genomes link Near Eastern and sub-Saharan African human populations. *Science* **360**, 548–552 (2018).

28. Q. Fu, *et al.*, The genetic history of Ice Age Europe. *Nature* **534**, 200–205 (2016).

29. C. E. G. Amorim, *et al.*, Understanding 6th-century barbarian social organization and migration through paleogenomics. *Nat. Commun.* **9**, 3547 (2018).

30. M. L. Antonio, *et al.*, Ancient Rome: A genetic crossroads of Europe and the Mediterranean. *Science* **366**, 708–714 (2019).

31. M. Lipatov, K. Sanjeev, R. Patro, K. R. Veeramah, Maximum Likelihood Estimation of Biological Relatedness from Low Coverage Sequencing Data. *bioRxiv*, 023374 (2015).

32. D. Popli, S. Peyrégne, B. M. Peter, KIN: a method to infer relatedness from low-coverage ancient DNA. *Genome Biol.* **24**, 10 (2023).

33. J. M. Monroy Kuhn, M. Jakobsson, T. Günther, Estimating genetic kin relationships in prehistoric populations. *PLoS One* **13**, e0195491 (2018).

34. H. Ringbauer, *et al.*, Accurate detection of identity-by-descent segments in human ancient DNA. *Nat. Genet.* **56**, 143–151 (2024).

35. J. Staples, *et al.*, PRIMUS: rapid reconstruction of pedigrees from genome-wide estimates of identity by descent. *Am. J. Hum. Genet.* **95**, 553–564 (2014).

36. H. R. Kunsch, The Jackknife and the Bootstrap for General Stationary Observations. *Ann. Stat.* **17**, 1217–1241 (1989).

37. L. Pejrani Baricco, “Bardonecchia (Torino), necropoli di ambito merovingio, in Longobardi” in *Longobardi. Un Popolo Che Cambia La Storia. Exhibition Catalog*, G. P. Brogiolo, F. Marazzi, C. Giostra, Eds. (SKIRA, 2017), pp. 76–77.

38. C. Giostra, E. Bedini, D. Caramelli, F. Mallegni, L. Pejrani Baricco, “Per una conoscenza dei Longobardi in Italia: primi risultati delle analisi genetiche su individui provenienti da necropoli del Piemonte” in *VI Congresso Nazionale Di Archeologia Medievale*, A. F. Fabio Redi, Ed. (All’Insegna del Giglio, 2012), pp. 448–453.

39. S. Vai, *et al.*, Genealogical relationships between early medieval and modern inhabitants of Piedmont. *PLoS One* **10**, e0116801 (2015).

40. L. Pejrani Baricco, “Un inedito complesso cimiteriale suburbano della Torino paleocristiana” in *Isole E Terraferma Nel Primo Cristianesimo: Identità Locale Ed Interscambi Culturali, Religiosi E Produttivi: Atti XI Congresso Nazionale Di Archeologia Cristiana*, R. Martorelli, A. Piras, P. G. Spanu, Eds. (2015), pp. 657–666.

41. L. Pejrani Baricco, S. Ratto, Torino, corso Palermo (centro direzionale Lavazza). Chiesa funeraria paleocristiana. *Quaderni della Soprintendenza Archeologica del Piemonte* **30**, 377–380 (2015).

42. G. Eriksson, *“Stable isotope analysis of humans”, The Oxford Handbook of the Archaeology of Death and Burial*, S. Tarlow, Ed. (Oxford University Press, 2013).

43. S. H. Ambrose, Preparation and characterization of bone and tooth collagen for isotopic analysis. *J. Archaeol. Sci.* **17**, 431–451 (1990).

44. J. A. Lee-Thorp, On isotopes and old bones. *Archaeometry* **50**, 925–950 (2008).

45. G. Müldner, M. P. Richards, Fast or feast: reconstructing diet in later medieval England by stable isotope analysis. *J. Archaeol. Sci.* **32**, 39–48 (2005).

46. F. D. Pate, Bone Chemistry and Paleodiet. *Journal of Archaeological Method and Theory* **1**, 161–209 (1994).

47. T. Douglas Price, An Introduction to the Isotopic Studies of Ancient Human Remains. *Journal of the North Atlantic, 2014(sp7)* **2014**, 71–87 (2014).

48. M. P. Richards, K. Britton, *Archaeological Science: An Introduction* (Cambridge University Press, 2020).

49. G. D. Farquhar, J. R. Ehleringer, K. T. Hubick, Carbon Isotope Discrimination and Photosynthesis. *Annu. Rev. Plant Physiol. Plant Mol. Biol.* **40**, 503–537 (1989).

50. M. P. Richards, “Isotope Analysis for Diet Studies” in *Archaeological Science: An Introduction*, (Cambridge University Press, 2020), pp. 125–144.

51. B. N. Smith, S. Epstein, Two categories of c/c ratios for higher plants. *Plant Physiol.* **47**, 380–384 (1971).

52. S. H. Ambrose, L. Norr, “Experimental Evidence for the Relationship of the Carbon Isotope Ratios of Whole Diet and Dietary Protein to Those of Bone Collagen and Carbonate” in *Prehistoric Human Bone: Archaeology at the Molecular Level*, J. B. Lambert, G. Grupe, Eds. (Springer Berlin Heidelberg, 1993), pp. 1–37.

53. C. M. Kellner, M. J. Schoeninger, A simple carbon isotope model for reconstructing prehistoric human diet. *Am. J. Phys. Anthropol.* **133**, 1112–1127 (2007).

54. K. A. Hemer, A. L. Lamb, C. A. Chenery, J. A. Evans, A multi-isotope investigation of diet and subsistence amongst island and mainland populations from early medieval western Britain. *Am. J. Phys. Anthropol.* **162**, 423–440 (2017).

55. M. H. O'Leary, Carbon Isotopes in Photosynthesis. *Bioscience* **38**, 328–336 (1988).

56. P. Szpak, Complexities of nitrogen isotope biogeochemistry in plant-soil systems: implications for the study of ancient agricultural and animal management practices. *Front. Plant Sci.* **5**, 288 (2014).

57. M. J. DeNiro, S. Epstein, Influence of diet on the distribution of carbon isotopes in animals. *Geochim. Cosmochim. Acta* **42**, 495–506 (1978).

58. B. Fry, Stable Isotope Diagrams of Freshwater Food Webs. *Ecology* **72**, 2293–2297 (1991).

59. N. J. P. Owens, "Natural Variations in 15N in the Marine Environment" in *Advances in Marine Biology*, J. H. S. Blaxter, A. J. Southward, Eds. (Academic Press, 1988), pp. 389–451.

60. M. J. Schoeninger, M. J. DeNiro, Nitrogen and carbon isotopic composition of bone collagen from marine and terrestrial animals. *Geochim. Cosmochim. Acta* **48**, 625–639 (1984).

61. M. L. Fogel, Nitrogen isotope tracers of human lactation in modern and archaeological populations. *In: Annual Report of the Director of the Geophysical Laboratory, Washington: Carnegie Institution, volume* **88**, 111 (1989).

62. B. T. Fuller, J. L. Fuller, D. A. Harris, R. E. M. Hedges, Detection of breastfeeding and weaning in modern human infants with carbon and nitrogen stable isotope ratios. *Am. J. Phys. Anthropol.* **129**, 279–293 (2006).

63. M. Jay, Breastfeeding and Weaning Behaviour in Archaeological Populations: Evidence from the Isotopic Analysis of Skeletal Materials. *Childhood in the Past* **2**, 163–178 (2009).

64. S. El Meknassi, *et al.*, Seawater 87Sr/86Sr ratios along continental margins: Patterns and processes in open and restricted shelf domains. *Chem. Geol.* **558**, 119874 (2020).

65. M. Marinato, Analisi isotopiche e bioarcheologia come fonti per lo studio del popolamento tra tardo antico e alto medioevo in Italia settentrionale. *Dati a confronto per le province di Bergamo, Modena e Verona* (2016).

66. G. Cox, J. Sealy, Investigating identity and life histories: isotopic analysis and historical documentation of slave skeletons found on the Cape Town foreshore, South Africa. *Int. J. Hist. Archaeol.* **1**, 207–224 (1997).

67. R. Longin, New method of collagen extraction for radiocarbon dating. *Nature* **230**, 241–242 (1971).

68. T. A. Brown, D. E. Nelson, J. S. Vogel, J. R. Southon, Improved Collagen Extraction by Modified Longin Method. *Radiocarbon* **30**, 171–177 (1988).

69. M. J. DeNiro, Postmortem preservation and alteration of in vivo bone collagen isotope ratios in relation to palaeodietary reconstruction. *Nature* **317**, 806–809 (1985).

70. E. J. Guiry, P. Szpak, Improved quality control criteria for stable carbon and nitrogen isotope measurements of ancient bone collagen. *J. Archaeol. Sci.* **132**, 105416 (2021).

71. I. Barbiera, *Changing Lands in Changing Memories. Migration and Identity during the Lombard Invasion (Premio Ottone d'Assia 2002)* (All'Insegna del Giglio, 2005).

72. A. Distelberger, Awarinnen: Frauen aus Gräbern des 7.-8. Jh. n. Chr. in Österreich. *Ethnographisch-archaologische Zeitschrift* **43**, 47–59 (2002).

73. G. Halsall, *Warfare and society in the barbarian west, 450-900* (Taylor & Francis Group, 2003) https:/doi.org/10.4324/9780203930076/warfare-society-barbarian-west-450-900-guy-halsall.

74. R Core Team, *R: A language and environment for statistical computing. R Foundation for Statistical Computing* (2013).

75. A. Dinno, *Dunn.Test: Dunn's test of multiple comparisons using rank sums* (2023).

76. M. R. Castiglioni Elisabetta, Broomcorn millet, foxtail millet and sorghum in north Italian Early Medieval sites. *Post-Classical Archaeologies* **3**, 131–144 (2013).

77. M. Baldoni, *et al.*, The medieval population of Leopoli-Cencelle (Viterbo, Latium): Dietary reconstruction through stable isotope analysis from bone proteins. *Journal of Archaeological Science: Reports* **24**, 92–101 (2019).

78. E. Castiglioni, M. Cottini, Il legno, i tessuti, i cuoi e gli altri materiali organici dalla necropoli longobarda e dall'abitato. *Presenze* (2004).

79. G. Riccomi, *et al.*, Stable isotopic reconstruction of dietary changes across Late Antiquity and the Middle Ages in Tuscany. *Journal of Archaeological Science: Reports* **33**, 102546 (2020).

80. M. Rottoli, Reflections on Early Medieval resources in northern Italy: The archaeobotanical and archaeozoological data. *Quat. Int.* **346**, 20–27 (2014).

81. F. Salvadori, The transition from late antiquity to early Middle Ages in Italy. A zooarchaeological perspective. *Quat. Int.* **499**, 35–48 (2019).

Legend for other supplementary materials

Movie S1. An animation showing the development of Collegno over time. The use of the site was divided into four temporal phases using a combination of archaeological chronology, radiocarbon dating, osteological and genetic information. Newly appearing individuals are colored based on the results of genetic clustering analyses, while earlier phases are shown in gray. The different colors represent the three pedigrees on the cemetery map: Blue: Pedigree I; Red: Pedigree II; Purple: Pedigree III. The cemetery developed from multiple cores in the center and eastern sections (a). After the abandonment of its central core an additional core was established to the west and the site expanded to multiple directions (b-c). In the last phase substantial reoccupation of the center is observable with new burials on top of the earlier ones (d)
