# Supplementary figures and images for: The role of emerging elites in the formation and development of communities after the fall of the Roman Empire
Source: Proc Natl Acad Sci U S A. 2024 Aug 19;121(36):e2317868121. doi: 10.1073/pnas.2317868121 (PMC11388374; doi:10.1073/pnas.2317868121)

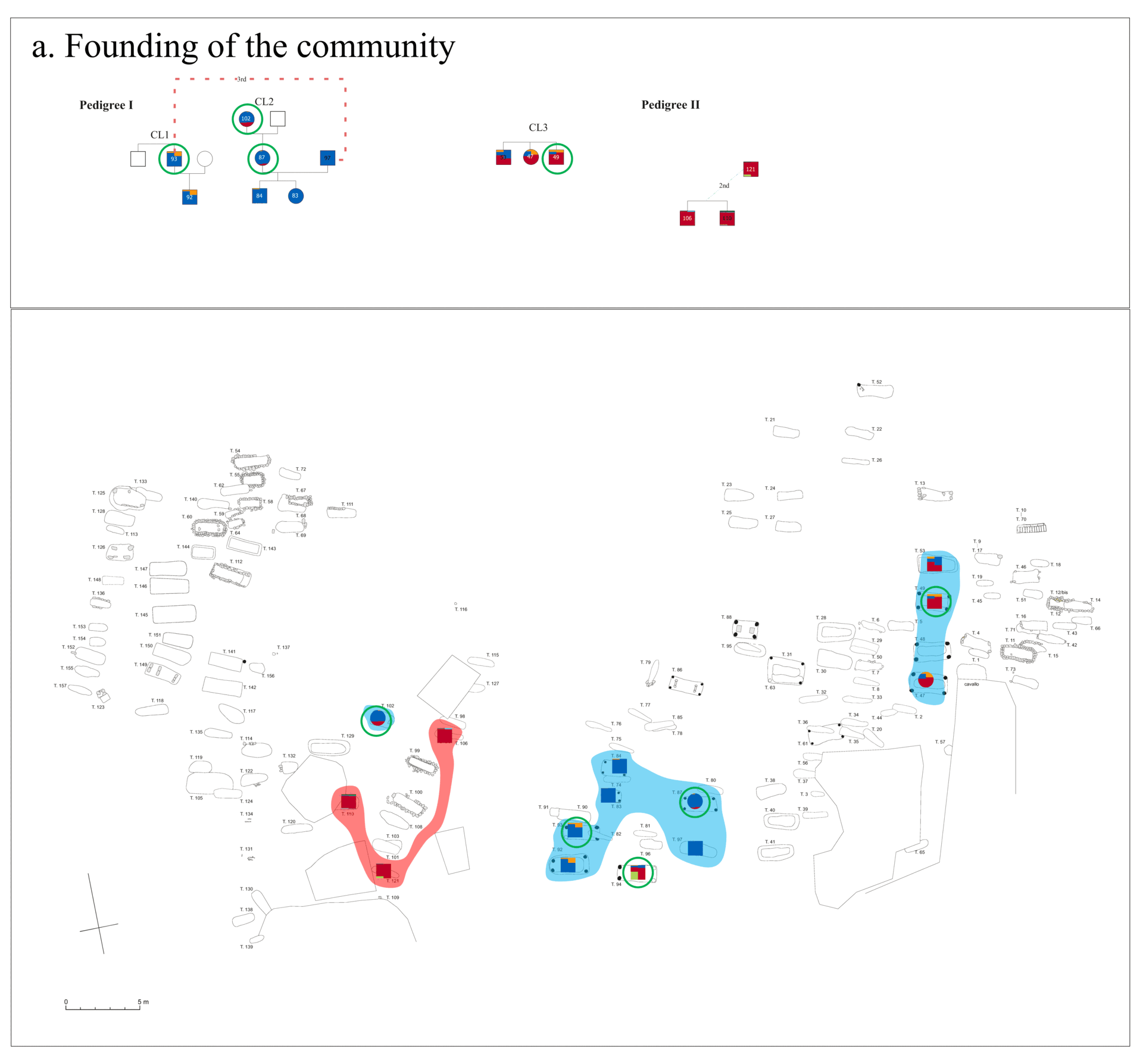

Supplement: Movie S1. — An animation showing the development of Collegno over time. The use of the site was divided into four temporal phases using a combination of archaeological chronology, radiocarbon dating, osteological and genetic information. Newly appearing individuals are colored based on the results of genetic clustering analyses, while earlier phases are shown in gray. The different colors represent the three pedigrees on the cemetery map: Blue: Pedigree I; Red: Pedigree II; Purple: Pedigree III. The cemetery developed from multiple cores in the center and eastern sections (a). After the abandonment of its central core an additional core was established to the west and the site expanded to multiple directions (b–c). In the last phase substantial reoccupation of the center is observable with new burials on top of the earlier ones (d) [file pnas.2317868121.sm01.gif]
